# A Clb/Cdk1-mediated regulation of Fkh2 synchronizes *CLB* expression in the budding yeast cell cycle

**DOI:** 10.1038/s41540-017-0008-1

**Published:** 2017-03-06

**Authors:** Christian Linke, Anastasia Chasapi, Alberto González-Novo, Istabrak Al Sawad, Silvia Tognetti, Edda Klipp, Mart Loog, Sylvia Krobitsch, Francesc Posas, Ioannis Xenarios, Matteo Barberis

**Affiliations:** 1 Synthetic Systems Biology and Nuclear Organization, Swammerdam Institute for Life Sciences, University of Amsterdam, Amsterdam, 1098 XH The Netherlands; 2 Max Planck Institute for Molecular Genetics, Berlin, 14195 Germany; 3 Vital-IT group, Swiss Institute of Bioinformatics, University of Lausanne, CH-1015 Lausanne, Switzerland; 4 Cell Signaling Unit, Departament de Ciències Experimentals i de la Salut, Universitat Pompeu Fabra, Barcelona, 08003 Spain; 5 Institute for Biology, Theoretical Biophysics, Humboldt University Berlin, Berlin, 10115 Germany; 6 Institute of Technology, University of Tartu, Tartu, 50411 Estonia

## Abstract

Precise timing of cell division is achieved by coupling waves of cyclin-dependent kinase (Cdk) activity with a transcriptional oscillator throughout cell cycle progression. Although details of transcription of cyclin genes are known, it is unclear which is the transcriptional cascade that modulates their expression in a timely fashion. Here, we demonstrate that a Clb/Cdk1-mediated regulation of the Fkh2 transcription factor synchronizes the temporal mitotic *CLB* expression in budding yeast. A simplified kinetic model of the cyclin/Cdk network predicts a linear cascade where a Clb/Cdk1-mediated regulation of an activator molecule drives *CLB3* and *CLB2* expression. Experimental validation highlights Fkh2 as modulator of *CLB3* transcript levels, besides its role in regulating *CLB2* expression. A Boolean model based on the minimal number of interactions needed to capture the information flow of the Clb/Cdk1 network supports the role of an activator molecule in the sequential activation, and oscillatory behavior, of mitotic Clb cyclins. This work illustrates how transcription and phosphorylation networks can be coupled by a Clb/Cdk1-mediated regulation that synchronizes them.

## Cell cycle control: Order in time achieved by dynamic coupling

A dynamic coupling of cyclin-dependent kinase with transcription factors in yeast offers insights into the timely cell cycle progression. An international team lead by Matteo Barberis from University of Amsterdam in The Netherlands studied the molecular mechanisms responsible for the coordination of DNA replication with cell division. The researchers have demonstrated how the sequential order of waves of mitotic cyclins activating cyclin-dependent kinase, or Cdk, is achieved by synchronizing Cdk with transcriptional activities. They have generated a mathematical model that predicts a cyclin/Cdk-mediated regulation of an activator molecule to stimulate mitotic cyclin expression. This prediction was successfully validated experimentally, identifying Forkhead transcription factors, or Fkh, as pivotal molecules. Cyclin waves are temporally synchronized by Fkh, and a mitotic Clb/Cdk1-mediated regulation of Fkh modulates cyclin expression. The findings reveal a novel principle of design, with kinase and transcription activities interlocked to guarantee a timely cell cycle.

## Introduction

In budding yeast, coordination of cell cycle transitions is achieved by periodic changes in the activity of the kinase Cdk1, which is regulated by temporal waves of expression of phase-specific cyclins.^1, 2^ Four waves of cell cycle-dependent transcription of cyclins are recognized: a wave of G1 cyclins (Cln1-3) is essential for passing START at the G1/S transition, whereas three waves of B-type cyclins (Clb5,6, Clb3,4, and Clb1,2) control DNA replication dynamics and mitotic entry/exit through S-to-M phases.^3, 4^ Successive oscillations of cyclin/Cdk1 activities ensure unidirectionality and correct timing of cell cycle transcriptional regulation. Although the yeast cell cycle has been studied extensively, it is not yet fully understood how these kinase activities interconnect precisely with the various transcriptional mechanisms driving a timely cell cycle progression. Cdk1 is not the main regulator of transcriptional oscillations,^5^ however, cyclin/Cdk1 activity contributes to the robustness of transcriptional oscillations by (i) modulating the activity of transcription factors, and (ii) acting as their effector to trigger the ordered program of cyclin expression.^6^ Moreover, transcription network and Cdk1-driven phosphorylation events are coupled by feed-forward loops to convert periodic oscillations of Cdk activity in transcriptional response.^7^ This ensures that the precise temporal sequence of cell cycle events is maintained.

A precise knowledge of the axis cyclin/Cdk1-transcriptional regulation throughout all cell cycle phases is still lacking. However, transcriptional regulation of the *CLB2* cluster that drives G2/M gene expression has been widely investigated, and the forkhead (Fkh) transcription factors Fkh1 and Fkh2 were identified as essential in this process.^8, 9^ Fkh1 and Fkh2 transcripts display a peak in S phase, and their periodic activity is dependent on cell cycle-regulated recruitment of the coactivator Ndd1 at the S/G2 transition.^8^ Although Fkh1 and Fkh2 have overlapping functions, only Fkh2 can associate with Ndd1 to regulate *CLB2* expression.^9^ Fkh2 and Ndd1 are phosphorylated by Clb5/Cdk1, thus triggering recruitment of Ndd1 to Fkh2 to *CLB2* promoter, and subsequently by Clb2/Cdk1 that further stabilizes the Fkh2-Ndd1 interaction by a positive feedback loop.^10^ Fkh2 phosphorylation is not abolished in a *clb5*Δ strain, suggesting that other Clb/Cdk1 complexes may also play a role in Fkh2 activation. Remarkably, in the absence of Clb3, Clb4, and Clb5, the *CLB2* promoter is not fully active with Clb2 being highly unstable.^11^ Thus, multiple Clb/Cdk1 complexes could phosphorylate Fkh2 and/or Ndd1 to generate a basal level of *CLB2* expression that would then rapidly increase due to the Clb2/Cdk1-mediated positive feedback loop.

Although many details of the transcription of cyclin genes are known,^6^ there is still a lack of understanding of precise transcriptional mechanisms regulating the relative timing of waves of *CLB* activation. In order to address this issue, we employed a simplified mathematical model^12^ of the cyclin/Cdk1 network to design new experiments addressing how timely waves of *CLB* expression may occur. Minimal models have been developed, which are able to account for properties of wild type cells.^13^ With a combined computational and experimental approach we unravelled that a cyclin/Cdk1-dependent regulation of the transcription factor Fkh2 is able to drive timely waves of mitotic *CLB* expression. Furthermore, a Boolean model based on the minimal number of interactions needed to capture the information flow of the Clb/Cdk1 network supports the role of an activator molecule in the sequential activation, and oscillatory behavior, of mitotic Clb cyclins. Our data reveal that Clb waves are temporally synchronized by Fkh2-mediated regulation of mitotic *CLB* genes, and that a Clb/Cdk1-mediated regulation of Fkh2 modulates the *CLB* cascade.

## Results

### A transcriptional regulation driving waves of mitotic *CLB* expression is predicted by kinetic modeling

**Fig. 1:**
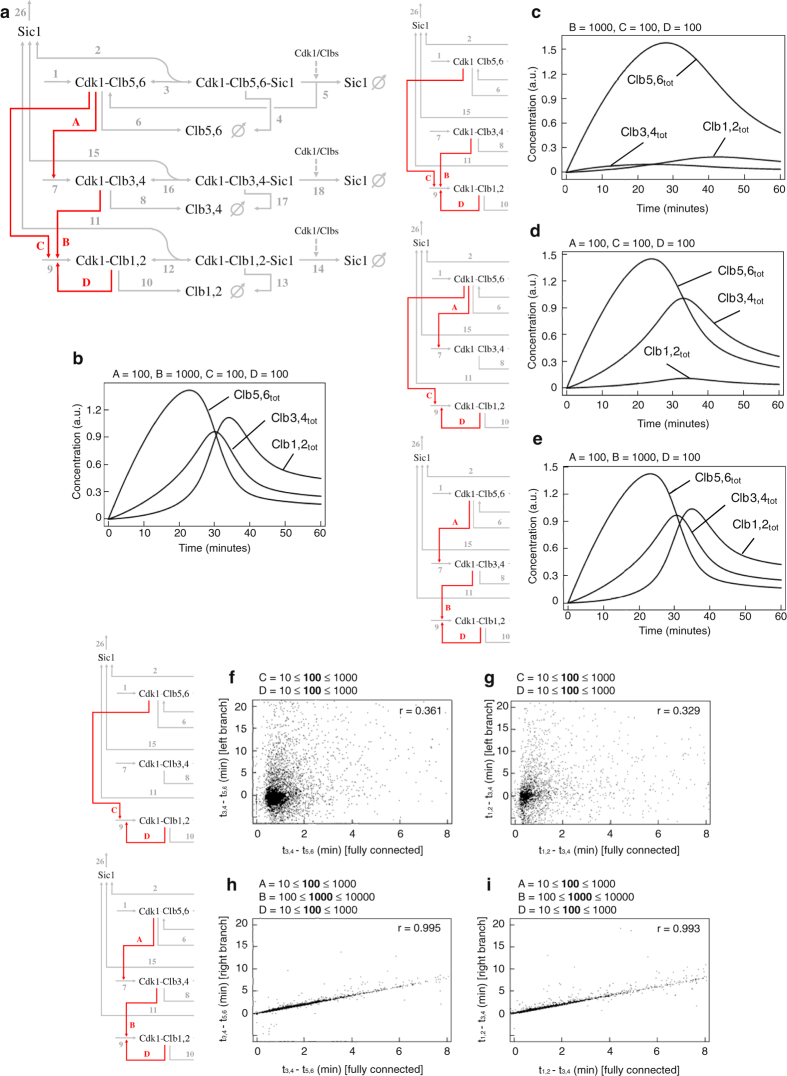
Kinetic model of Clb/Cdk1 regulation and computational time courses of total Clb cyclins levels. **a** Network highlighting in *red* the sequential transcriptional activation of Clb cylins, thereby Clb/Cdk1 activities (*k*_A_, *k*_B_, *k*_C,_ and *k*_D_). **b** Simulations of the network in Fig. 1a are carried out with standard values of parameters, as reported in Supplementary Text for details.^12^ **c**–**e** Model variants were generated starting from the network in Fig. 1a by varying values of *k*_A_ (**c**), *k*_B_ (**d**), and *k*_C_ (**e**), as indicated on each simulation panel and described in the text. The model variants were implemented by ordinary differential equations, with the parameters used for the simulations having the same value among all variants (see Supplementary Text for model description and the full set of equations).^12^ **f**–**i** Prediction and validation of the transcriptional regulation responsible for the delay between waves of Clb cyclins. The graphs report the time delay observed between maximum levels (peaks) of Clb cyclins for binary combinations between the minimal model—where Clb/Cdk1 complexes are connected via four transcriptional regulations—and two model variants, independently. **f**, **g** Time delay calculated for the left branch (only *k*_C_ active) between Clb5,6 and Clb3,4 (*t*_3,4_−*t*_5,6_) (**f**) and Clb3,4 and Clb1,2 (*t*_1,2_−*t*_3,4_) (**g**). **h**, **i** Time delay calculated for the right branch (only *k*_A_ and *k*_B_ active) between Clb5,6 and Clb3,4 (*t*_3,4_−*t*_5,6_) (**h**) and Clb3,4 and Clb1,2 (*t*_1,2_−*t*_3,4_) (**i**). Each parameter of the network may vary from its selected value to the same value multiplied for a random real value comprised between 0.1 and 10, as indicated on each simulation panel

To investigate whether a linear cascade of transcriptional activation is compatible with sequential waves of Clb cyclins, we employed a minimal kinetic model previously published by Barberis and colleagues,^12^ for which the system property “Clb wave formation”, is robust to parameter’s choice. We systematically compared networks that differ in Clb/Cdk1-mediated regulations at mitotic *CLB* promoters (indicated in red color in Fig. 1a). Specifically, we investigated the role of: (i) Clb5,6/Cdk1 on *CLB3,4* transcription (Fig. 1a, arrow A, *k*_A_), (ii) Clb3,4/Cdk1 on *CLB1,2* transcription (Fig. 1a, arrow B, *k*_B_), and (iii) Clb5,6/Cdk1 on *CLB1,2* transcription (Fig. 1a, arrow C, *k*_C_). Simulation of the minimal network showed alternate Clb waves in time, each wave deriving from the sum of all complexes in which each Clb is present (Fig. 1b; see Supplementary Text for details on model equations and kinetic parameters).^12^ We analyzed three versions of this network, where reactions *k*_A_ (Fig. 1c), *k*_B_ (Fig. 1d) and *k*_C_ (Fig. 1e) were neglected, respectively; conversely, the Clb1,2/Cdk1-mediated feedback loop on *CLB1,2* (Fig. 1a, arrow D, *k*_D_) was always present. In Fig. 1c computed time courses of total levels of Clb5,6, Clb3,4 and Clb1,2 are shown when *k*_A_ is removed. The simulation revealed no temporal coordination between the times of Clb appearance, with Clb3,4 peaking earlier than Clb5,6. Varying the value of *k*_A_ resulted in a non correct order (Supplementary Fig. S1a) or to a steady state level (Supplementary Fig. S1b) of Clb waves, respectively. This would lead in vivo to a high Clb2 level and, thus, to cells arrested before cell division. When *k*_B_ was removed from the network, no temporal coordination was observed between the peaks of Clb3,4 and Clb1,2 (Fig. 1d). Varying the value of *k*_B_ resulted in Clb3,4 and Clb1,2 peaks appearing at the same time (Supplementary Fig. S1c) or to a steady state level of Clb1,2 (Supplementary Fig. S1d). Remarkably, when *k*_C_ was removed from the network, oscillation of Clb cyclins was observed (Fig. 1e); this behavior was maintained after decreasing (Supplementary Fig. S1e) or increasing (Supplementary Fig. S1f) the value of *k*_C_. In addition, simulating the model with a wide range of parameter sets show that waves of Clb cyclins are brought about more consistently when the linear cascade is present, and that the value of *k*_C_ is lower than those of *k*_A_ and *k*_B_ (data not shown). These results suggest that a linear cascade activating Clb cyclins may be required to generate staggered waves of Clb/Cdk1 kinase activities throughout cell cycle progression. The only regulation that has been shown to promote *CLB2* transcription occurs via Clb5/Cdk1-mediated activation of the transcription factor Fkh2^10^ (Fig. 1a, regulation C). Our computational analysis shows that this regulation is *per se* not sufficient to temporally coordinate Clb waves in absence of either regulation A or B. Thus, two possible scenarios are predicted: (1) *CLB2* transcription occurs only through a Clb5/Cdk1-mediated activation of Clb3, or (2) a moderate, but not high, regulation C may contribute to the occurrence of serial Clb waves.

To investigate the transcriptional requirements for the formation of Clb cyclin waves, we tested the impact of a specific parameter choice on the delay between their peaks by performing a global sensitivity analysis (see Supplementary Materials and Methods for details).^12^ We compared the minimal network to the versions where only *k*_C_ is active (left branch) or where only *k*_A_ and *k*_B_ are active (right branch). The pairwise comparison of different network structures showed that a change of parameter values affects strongly the distance between peaks of any Clb cyclin (Fig. 1 and Supplementary Fig. S1). If only the left branch is active, time delays tended to be smaller for the distance between Clb5,6–Clb3,4, and Clb3,4–Clb1,2 (Fig. 1f, g) and, consequently, between Clb5,6–Clb1,2 (Supplementary Fig. S1g). Remarkably, for the three peak distances, a positive effect on time delays was observed when only the right branch is active (Fig. 1h, i, Supplementary Fig. S1h), being the correlation indexes close to 1. Together, these analyses predict that regulation of time delays between Clb cyclins, thereby their oscillations, is essentially triggered by a linear cascade of *CLB* activation.

### Fkh2 is responsible for the timely onset of Clb3 protein

**Fig. 2:**
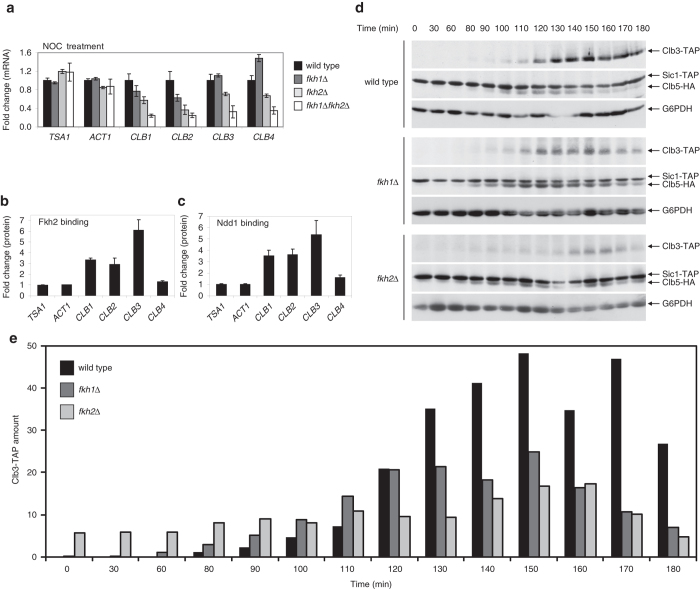
Fkh1 and Fkh2 regulate dynamics of mitotic Clb cyclins in a cell cycle-dependent manner. **a** Quantitative real-time PCR of mitotic *CLB* transcripts in yeast cells treated with nocodazole (NOC). Total mRNA was isolated from arrested wild type, *fkh1*Δ, *fkh2*Δ, and *fkh1*Δ*fkh2*Δ cells, and *CLB1*, *CLB2*, *CLB3,* and *CLB4* mRNA levels were measured. *ACT1* and *TSA1* genes were used as negative controls, as they are not affected by cell cycle dynamics. Error bars on the histograms represent SDs from the mean of three independent experiments; *p*-values are indicated on the histograms in Supplementary Fig. S2a. Nocodazole-arrested cells show that *fkh2*Δ affects both *CLB3* and *CLB4* transcript accumulation, whereas *fkh1*Δ affects *CLB4* transcript accumulation. **b**, **c** Binding of Fkh2 (**b**) and Ndd1 (**c**) to mitotic *CLB* promoter regions. Chromatin immunoprecipitation was performed by precipitating protein/DNA complexes from cells grown in exponential phase using an anti-Myc antibody. *ACT1* and *TSA1* genes were used as negative controls, whereas *CLB1* and *CLB2* genes as positive controls. Error bars on the histograms represent SDs from the mean of three independent experiments. **d**, **e** Fkh2 controls the timing of Clb3 protein expression. **d** Time course of Clb2-18Myc, Clb3-TAP, Clb5-HA and Sic1-TAP are shown for wild type (YAN49), *fkh1*Δ (YAG20), and *fkh2*Δ (YAG21) strains. Yeast cells were synchronized by centrifugal elutriation in YPD at 30 °C, released into fresh YPD at 30 °C, and sampled for western blot analysis at the indicated times. Clb3 levels were quantified in wild type, *fkh1*Δ and *fkh2*Δ strains (**e**). The experiments were performed at the same time, and the membranes processed identically, with the same antibody aliquots and then exposed to the same levels. *fkh1*Δ resulted in an decreased amount of Clb3 produced and no time delay as compared to wild type, whereas *fkh2*Δ resulted in both a decreased amount of Clb3 produced and a time delay as compared to wild type. The result is representative of three independent experiments

To investigate the linear cascade of regulation predicted by the kinetic modeling, we tested the role of the transcription factors Fkh1 and Fkh2 that are active in the temporal window where mitotic Clb cyclins are transcribed.^14, 15^ Mitotic *CLB* mRNA levels were measured in *fkh1*Δ*, fkh2*Δ, or *fkh1*Δ*fkh2*Δ strains by quantitative real-time PCR on cells arrested in G2/M phase with nocodazole (Fig. 2a) and in S phase with hydroxyurea (Supplementary Fig. S2b). These treatments revealed reduced *CLB1* and *CLB2* mRNA levels in *fkh2*Δ and *fkh1*Δ*fkh2*Δ mutants and a less prominent effect of *fkh1*Δ as compared to wild type, as previously observed.^16^ This pattern was observed also for *CLB3*, whereas *CLB4* mRNA levels were affected in *fkh2*Δ and *fkh1*Δ*fkh2*Δ mutants only in nocodazole treatment but were increased in *fkh1*Δ cells (Fig. 2a). Thus, Fkh2 may act as positive regulator of *CLB3* and *CLB4* transcription. Contrarily, Fkh1 may act as negative regulator of *CLB4* transcription, providing further support to earlier experimental and computational analyses proposing that Fkh1 binds to the *CLB4* promoter.^15, 17^ We then investigated whether Fkh1 and Fkh2 bind to *CLB* promoters by chromatin immunoprecipitation (ChIP) (see Supplementary Materials and Methods). An enrichment of Fkh2 (Fig. 2b) and Fkh1 (Supplementary Fig. S2c) was observed at *CLB1* and *CLB2* promoters, as expected. Remarkably, a significative enrichment of Fkh1 and Fkh2 was detected at the *CLB3* promoter, consistent with RNA Pol II occupancy data (Supplementary Fig. S2d), but not at the *CLB4* promoter. We conclude that Fkh1 and Fkh2 regulate *CLB3* transcription. Remarkably, a strong enrichment of Ndd1, coactivator of Fkh2, at the *CLB3* promoter was also observed (Fig. 2c), consistent with the fact that Ndd1 is required for Fkh2 periodic activity,^8^ and that the Fkh2/Ndd1 complex may regulate *CLB3* transcription.

To determine the direct role of Fkh1 and Fkh2 in Clb3 regulation, we investigated its protein levels in *fkh1*Δ or *fkh2*Δ strains in a time course experiment with G1-elutriated cells (see Supplementary Materials and Methods for details). In the same yeast strain, the levels of Sic1, Clb5, and Clb2 (the latter not visualized) were used as reference.^12^ In the *fkh1*Δ strain the temporal window of maximal Clb3 expression is similar, but its levels accumulate at a lower amount as compared to wild type (Fig. 2d, e). The *fkh2*Δ strain instead accumulates Clb3 levels earlier as compared to wild type, and the temporal window of maximal Clb3 expression is delayed, with a protein amount strongly reduced, as compared to wild type (Fig. 2d, e); the delay was also confirmed by FACS analyses (Supplementary Fig. S2e). Notably, Clb3 may be still produced in *fkh2*Δ cells, indicating that Fkh1 or other, yet unknown transcription factors, may partially overlap with Fkh2 to promote *CLB3* transcription. Together, these data confirm our model prediction, and demonstrate the role of Fkh2 in the regulation of the temporal appearance of Clb3 protein levels. Of note, we observe that a premature accumulation of Clb5 protein levels occurs upon Fkh2 deletion, already at early time points of the time course (Fig. 2d). However, our quantitative real-time PCR analyses did not show any effect of Fkh2 on *CLB5* mRNA levels (see Supplementary Text and Supplementary Fig. S2f). This suggests that other mechanisms, such as a reduced degradation of Clb5 due to a reduced *CLB2* transcription/translation and of Clb2/Cdk1 complexes that activate the APC machinery,^18^ may be responsible for the early accumulation of Clb5 protein levels.

Remarkably, since we discovered that Fkh2 promotes *CLB3* transcription, Clb3/Cdk1 may phosphorylate this transcription factor at *CLB3* promoter, through a positive feedback loop to produce additional Clb3 protein, and at *CLB2* promoter to produce Clb2. In order to explore computationally the possible contribution of these regulations, we investigated their ability to generate the characteristic behavior of the waves of Clb cyclins. Our simulations revealed that a positive feedback loop on *CLB3* transcription may provide an additional, but not *per se* sufficient mechanism to shape Clb waves (see Supplementary Text and Supplementary Fig. S3).

### Clb3, but not Clb2, co-localizes with Fkh2/Ndd1in HU-treated cells

**Fig. 3:**
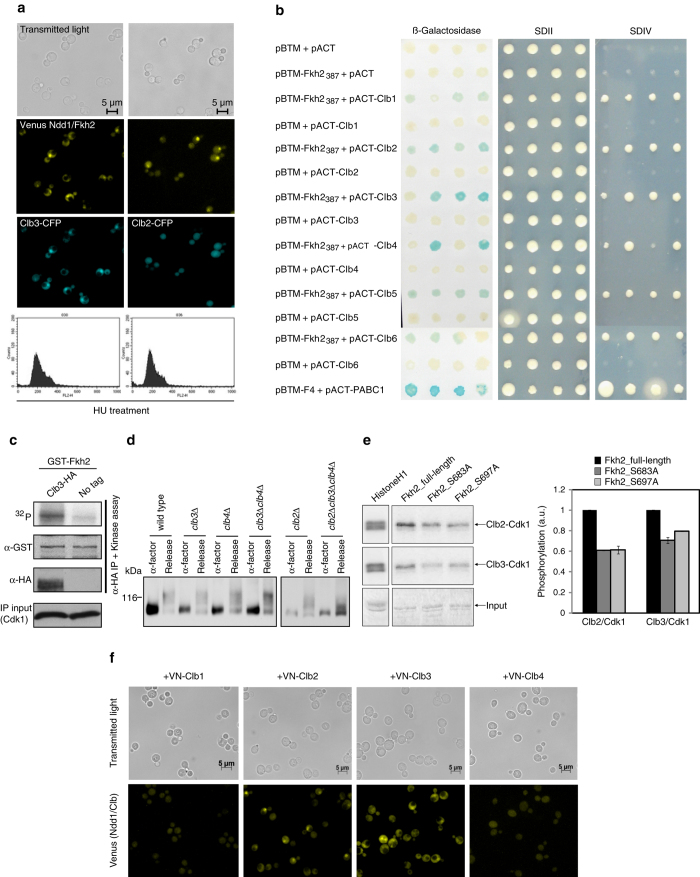
Clb3/Cdk1 co-localizes with, interacts and phosphorylates the Ndd1/Fkh2 complex. **a** Co-colocalization of Clb3 and Clb2 with the Ndd1/Fkh2 complex. Yeast cells expressing Ndd1-VC and carrying Clb3-CFP or Clb2-CFP integrated in the genome were transformed with the plasmid p426-VN-Fkh2. Selected transformants were synchronized in S phase with hydroxyurea (HU) and monitored for the fluorescent (BiFC) signal. A co-localization with the BiFC signal is shown for Clb3. Growth arrest was examined by staining with propidium iodide and FACS analysis. **b** Fkh2 C-terminal region (Fkh2_387_) associates with Clb cyclins by Yeast-two-Hybrid assay. Yeast cells expressing the fusion proteins LexA-Fkh2_387_ (pBTM-Fkh2_387_) and AD-Clb1-6 (pACT-Clb1-6) were spotted onto SDII and SDIV selective media or on a membrane for detection of the β-galactosidase activity. The interaction between ATXN2-FD and PABC was used as positive control.^12^ At least three independent transformations have been performed, and four clones were tested in each experiment. **c** Fkh2 is a substrate of Clb3/Cdk1 kinase activity in vitro. Yeast lysate expressing Clb3-HA was incubated with GST-Fkh2 expressed and purified from *E. coli*. After the kinase assay, the membrane was plotted using antibodies against GST (to detect Fkh2), HA (to detect Clb3), and PSTAIR (to detect Cdk1). The accuracy of the result was verified by monitoring that a similar amount of Cdk1 kinase in the immunoprecipitation (IP), and of GST-Fkh2 was used in the assay. **d** Fkh2 is phosphorylated in vivo by Clb3/Cdk1 kinase activity. Wild type, *clb3*Δ, *clb4*Δ, *clb3*Δ*clb4*Δ, *clb2*Δ, and *clb2*Δ*clb3*Δ*clb4*Δ were grown at 25 °C in YPD medium and then synchronized in G1 phase with α-factor, before releasing them into fresh medium for 50 min (Release). Protein extracts were analyzed in 6% polyacrylamide gel electrophoresis (PAGE) 10 μM Phos-tag gel and Fkh2-6HA was followed by western blot. **e** Fkh2 phosphosite specificity of Clb3/Cdk1. Fkh2 full-length and mutants (S684A and S697A) were isolated from bacterial protein extracts and phosphorylated by Clb2/Cdk1 and Clb3/Cdk1 isolated from yeast extracts containing overexpressed Clb2-TAP or Clb3-TAP. An excess of purified Fkh2 was added to fully saturate the cyclin/Cdk1 complexes. Kinase reactions were started by adding purified Clb2/Cdk1 or Clb3/Cdk1, and ^32^P-ATP to the mixture. Histone H1 was used as a control, and standard sodium dodecyl sulfate-PAGE was used to separate phosphorylated species. Quantification of the phosphorylation extent (arbitrary units) is realized with PhosphoImager, as average from two different time points; values relative to a full-length Fkh2 are shown. The result is representative of three independent experiments. **f** Clb3 associates with Ndd1 by BiFC. Yeast cells expressing the fusion protein Ndd1-VC were transformed with the plasmids p426-VN-Clb1, p426-VN-Clb2, p426-VN-Clb3, and p426-VN-Clb4, respectively. Detection of the fluorescent (BiFC) signal was revealed by fluorescence microscopy

The transcription factor Fkh2 acts in a DNA-bound complex with the transcription factor Mcm1 to regulate cell cycle-dependent expression of the *CLB2* cluster, and binding of Fkh2 requires prior binding by Mcm1.^9, 10^. Since Fkh2 is involved in the transcription of both *CLB3* and *CLB2* genes, Clb5/Cdk1 may phosphorylate this transcription factor at both promoters. Our kinetic model predicted an involvement of an activator molecule—which we have shown to be the transcription factor Fkh2—by Clb3/Cdk1, in order to promote *CLB2* expression. To address this aspect, we first investigated whether Clb3 co-localized with Fkh2. We followed the association of Fkh2 and its coactivator Ndd1 in time by bimolecular fluorescence complementation (BiFC) (see Supplementary Materials and Methods for details and Fig. S4a), after α-factor-mediated synchronization of cells in G1 phase. The presence of a yellow fluorescent signal, also called BiFC signal, highlighted a nuclear localization of the Fkh2/Ndd1 complex formation starting from the early S to G2/M phase (Supplementary Fig. S4b). Specifically, the BiFC signals stably appear at 30 min after α-factor synchronization, which corresponds to the time at which the *CLB2* promoter is active^8^ following the recruitment of Ndd1 to chromatin in a cell cycle-specific manner.^19^ Subsequently, Clb3-CFP was integrated in the genome of these cells under control of the endogenous promoter to follow its co-localization with the BiFC signals, and Clb2-CFP was used as control for the experiment. In hydroxyurea-treated cells, Clb3 clearly co-localizes with the BiFC signals, whereas a very low, not localized Clb2-CFP signal is detected (Fig. 3a). The co-localization of Clb2 with the Ndd1/Fkh2 complex occurs later in mitosis, as previously shown^10^ (data not shown). This finding indicates that Clb3 co-localizes with the Fkh2/Ndd1 complex before Clb2 accumulation, and suggests a functional interaction between Clb3 and this complex to activate the *CLB2* promoter.

### Clb3 interacts with Fkh2/Ndd1 and, together with Cdk1, phosphorylates Fkh2

To address the potential association between Clb3 and Fkh2, we performed a Yeast-two-Hybrid assay. Bait (pBTM117) and prey (pACT4) constructs were generated for Fkh2 and Clb cyclins (see Supplementary Materials and Methods for details),^12, 20^ with the potential interacting partners being overexpressed. Fkh2 full-length and a truncated, C-terminal region of the protein Fkh2_387_ (amino acids 387–862) (see Supplementary Fig. S5a for a schematic representation of the constructs used in this study) were tested. Both Fkh2 full-length (Supplementary Fig. S5b) and Fkh2_387_ (Fig. 3b) showed a clear interaction with Clb3 as well as with the other Clb cyclins. The interactions between Fkh2_387_ and Clb cyclins were validated independently by a GST pull-down assay (Supplementary Fig. S5e).

Subsequently, Fkh2 phosphorylation by the Clb3/Cdk1 complex was tested by immunoprecipitating Clb3/Cdk1 from a yeast lysate that expressed HA-tagged Clb3 and incubating it with bacterially expressed and purified GST-Fkh2 (see Supplementary Materials and Methods for details). We observed that Fkh2 is a substrate of the Clb3-associated kinase activity, as compared to a wild-type strain where Clb3 was not tagged (negative control) (Fig. 3c). Prompted by this result, we have tested whether Clb3/Cdk1 was able to phosphorylate Fkh2 in vivo. To this aim, Fkh2 mobility was detected in a Phos-tag gel in wild type, *clb3*Δ, *clb4*Δ, and *clb3*Δ*clb4*Δ synchronized in G1 phase with α-factor. Fkh2 was phosphorylated in a similar manner in all tested strains (Fig. 3d, left panel). Then, we hypothesized that, in absence of Clb3 and Clb4, Clb2 could replace them. Thus, we have introduced a *clb2*Δ mutation in wild type and *clb3*Δ*clb4*Δ strains, and observed a clear decrease in Fkh2 mobility in the *clb2*Δ*clb3*Δ*clb4*Δ strain as compared to the *clb2*Δ strain, indicating a role for Clb3,4 in Fkh2 phosphorylation (Fig. 3d, right panel). In order to provide further evidence of the Clb3/Cdk1-mediated phosphorylation on Fkh2, we have tested the kinase activity of Clb3/Cdk1 isolated from a wild-type yeast lysate on a bacterially expressed Fkh2, either full-length or variants carrying single point mutations in two phosphorylation sites (S683 and T697). Both phosphosites are located in the C‐terminal region of Fkh2, and have been shown to be phosphorylated by Clb2/Cdk1.^10^ Deletion of these residues leads to a reduction of the amount of Fkh2 phosphorylated.^10^ We observed that the Clb3-associated kinase activity is reduced on both mutants between 20% to 40% as compared to a full-length Fkh2 (Fig. 3e), indicating that the phosphosites S683 and T697 mediate the activation of Fkh2 by all Clb/Cdk1 kinase complexes. Altogether, our data confirm that Fkh2 interacts with Clb5, as reported previously,^10^ and with all Clb cyclins, and show that Clb3/Cdk1 interacts with, and phosphorylates, Fkh2.

Besides interacting with and phosphorylating Fkh2, Clb3,4/Cdk1 may also play a role in Ndd1 phosphorylation in vivo to prime *CLB2* transcription. Associations of Ndd1 with Clb2 and Clb3 have been detected in a high-throughput genome-wide screening for complexes,^21^ but they were not independently validated. To test the Ndd1/Clb2 and Ndd1/Clb3 potential associations, haploid yeast cells expressing the C-terminal region of the Venus protein fused to the C-terminal region of Ndd1 (Ndd1-VC) were transformed with a plasmid carrying the N-terminal region of the Venus protein fused to the C-terminal region of Clb1-4 (VN-Clb1-4). The BiFC signal was observed only for the Ndd1/Clb2 and Ndd1/Clb3 pairs (Fig. 3f). These interactions were further validated by Yeast-two-Hybrid (Fig. S5f) and GST pull-down (Supplementary Fig. S5g) assays, respectively. Our data, together with the evidence showing that Ndd1 is a substrate of the Clb3-associated kinase activity,^22^ support the hypothesis that the Fkh2/Ndd1 complex may be regulated by all Clb/Cdk1 complexes.

### Clb5/Cdk1 and Clb3/Cdk1 are responsible for a sequential mitotic *CLB* expression

**Fig. 4:**
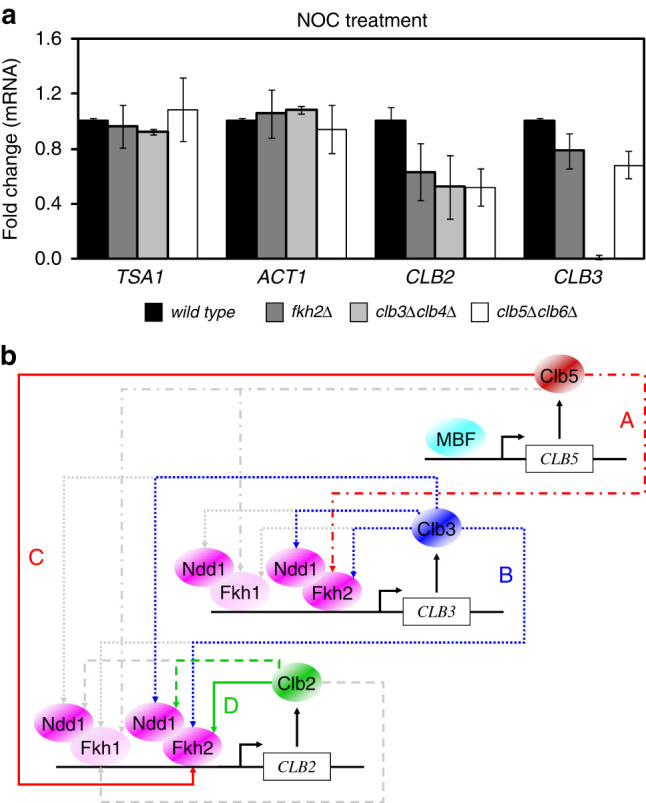
**a** Quantitative real-time PCR of mitotic *CLB* transcripts in yeast cells treated with nocodazole. Total mRNA was isolated from arrested wild type, *fkh2*Δ, *clb3*Δ*clb4*Δ, or *clb5*Δ*clb6*Δ cells, and *CLB2* and *CLB3* mRNA levels were measured. *ACT1* and *TSA1* genes were used as negative controls, as they are not affected by cell cycle dynamics. Error bars on the histograms represent SDs from the mean of three independent experiments; *p*-values are indicated on the histograms in Supplementary Fig. S5h. Nocodazole-arrested cells show that *clb3*Δ*clb4*Δ affects *CLB2* transcript accumulation, whereas *clb5*Δ*clb6*Δ affects both *CLB2* and *CLB3* transcript accumulation. **b** Schematic view of the regulatory connections involving the Fkh/Ndd1 and Clb/Cdk1 complexes. Colored *solid arrows* refer to the known interactions reported in literature; colored *dashed arrows* refer to the newly unraveled interactions between Fkh2/Ndd1 and Clb/Cdk1 complexes; *gray dashed arrows* refer to the newly unraveled interactions between Fkh1/Ndd1 and Clb/Cdk1 complexes. See text for details

The data presented provide an overall scenario in which the various Clb/Cdk1 complexes are responsible for the transcription of both *CLB3* and *CLB2* after phosphorylation, and activation, of the transcription factor Fkh2. Thus, this scenario predicts that the first Clb/Cdk1 complex activated, Clb5/Cdk1, would promote the transcription of *CLB3* and activation of the next kinase complex in the signaling cascade, Clb3/Cdk1, which ultimately—together with Clb5/Cdk1—would be responsible for the transcription of *CLB2* and activation of the last kinase complex of the cascade, Clb2/Cdk1. To investigate the role of each Clb/Cdk1 complex on *CLB* transcription suggested by both computational and experimental analyses, we tested the influence of Clb5,6 and Clb3,4 on both *CLB3* and *CLB2* transcript levels. Mitotic *CLB* mRNA levels were measured in *fkh2*Δ*, clb3*Δ*clb4*Δ, or *clb5*Δ*clb6*Δ strains after nocodazole treatment by quantitative real-time PCR. The *fkh2*Δ strain was used as a control for the experiment, leading to a reduction of both *CLB2* and *CLB3* mRNA levels as compared to wild type (Fig. 4a), as shown in Fig. 2a. Reduced *CLB2* mRNA levels were observed in *clb3*Δ*clb4*Δ mutants, whereas no *CLB3* transcripts were detected, as expected (Fig. 4a). Furthermore, reduced mRNA levels were observed for both *CLB2* and *CLB3* in *clb5*Δ*clb6*Δ mutants as compared to wild type, with the former being strongly affected as compared to the latter (Fig. 4a). This result indicates that Clb5,6 promotes *CLB3* transcription, and that both Clb5,6 and Clb3,4 impact on *CLB2* transcription (Fig. 4a). Thus, this evidence recapitulates both computational and experimental analyses, indicating that both Clb5/Cdk1 and Clb3/Cdk1 promote *CLB2* transcription, and that their progressive activation through Fkh2 guarantees timely waves of Clb cyclins throughout cell cycle progression.

In summary, our data support the hypothesis that the Fkh2/Ndd1 complex may be regulated by all Clb/Cdk1complexes for the activation of *CLB3* and *CLB2* promoters (Fig. 4b, colored dotted lines). Regulation mediated by Clb/Cdk1 complexes might occur also on Fkh1/Ndd1 (Fig. 4b, gray dotted lines), as we observed its interaction with some of the Clb cyclins (Supplementary Fig. S6). Remarkably, cooperativity between Ndd1 and Fkh1 was predicted by computational work,^23^ and we observed this specific interaction both in vitro and in vivo (Supplementary Fig. S7).

### A linear *CLB* cascade is required to generate temporal oscillations of Clb waves

**Fig. 5:**
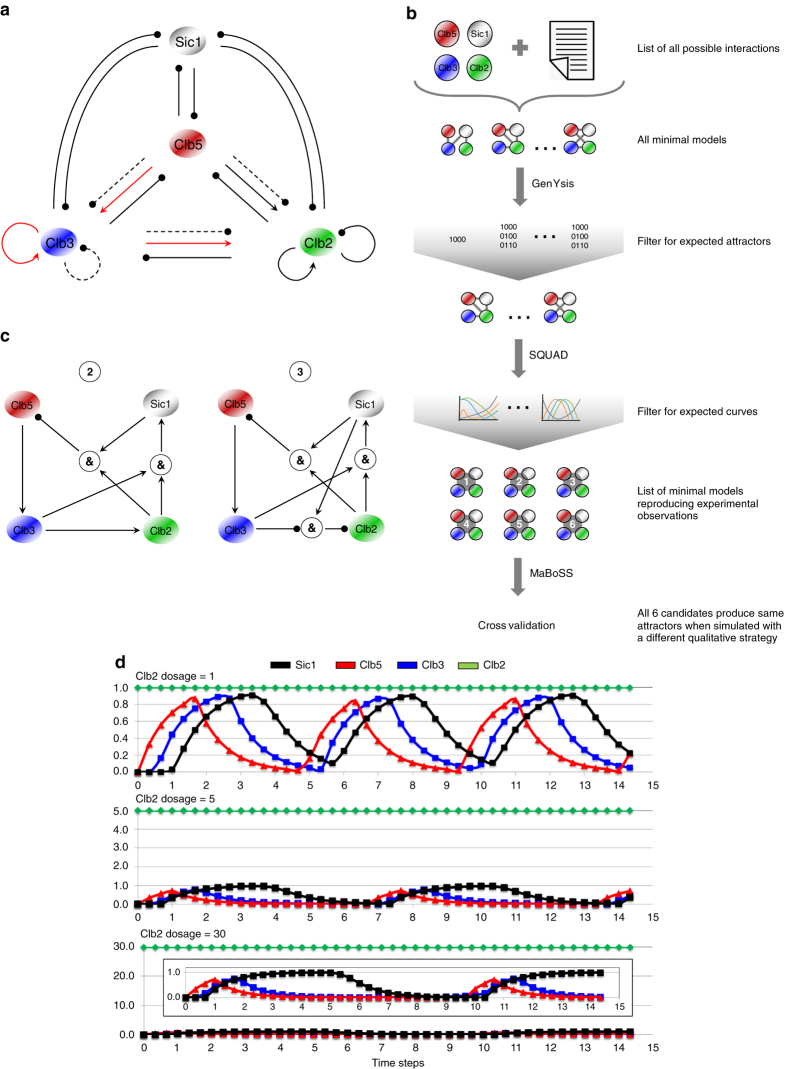
Expected oscillations in the Clb cyclin network and definition of Boolean attractor candidates. **a** Minimal prior knowledge network (PKN) encompassing the interactions among Clb5 (*red*), Clb3 (*blue*), Clb2 (*green*), and their inhibitor Sic1 (*black*). Known interactions are indicated with *solid lines*.^12^ For the newly unraveled interactions, both potential activatory and inhibitory regulations are considered: interactions mimicking positive regulations of an early Clb/Cdk1 complex on the next one through the Fkh2 transcription factor are indicated with *solid red lines*; interactions mimicking negative regulations of an early Clb/Cdk1 complex on the next one through the Fkh2 transcription factor are indicated with *dashed black lines*. **b** Boolean modeling strategy. Novel and known interactions occurring among Clbs and Sic1 were implemented in the models, which were then filtered for the expected attractors (by using GenYsis) and for the expected curves (by using SQUAD). The collection of minimal models reproducing the experimental observations was then validated by using a different qualitative strategy (MaBoSS). **c** Minimal candidate models reproducing experimental Clb and Sic1 waves and satisfying *CLB2* overexpression experiments.^27^ The minimal models 2 and 3 satisfy the known experimental conditions (wild type, *SIC1* overexpression and *sic1*Δ) by both Boolean simulations (GenYsis) and standarized ODE simulations (SQUAD). *Arrows* and *circles* denote activations and inhibitions, respectively. **d** SQUAD simulations of *CLB2* overexpression experiments for the minimal model 2. From *top* to *bottom* are plotted simulations of an increase dosage of Clb2: 1 (*upper panel*), 5 (*mid panel*), and 30 (*bottom panel*). The insert in the *bottom panel* represents a magnification of Sic1, Clb5, and Clb3 oscillations when Clb2 is set to 30. It shall be noted that by the computational time *t* = 15 the cycles are progressively reduced when increasing the Clb2 level. The results are in agreement with the experimental observations.^27^

To theoretically investigate the contribution of the known regulatory interactions of the minimal cyclin network (Fig. 1a)^12^ as well as of the newly unraveled transcriptional cascade activating *CLB* genes to the waves of Clb activation, we employed an independent, qualitative modeling approach, the Boolean modeling, to identify the possible network structure(s) able to reproduce this oscillatory behavior. A prior knowledge network (PKN) of the interactions among four nodes encompassing the mitotic cyclins Clb5, Clb3 and Clb2, and the cyclin-dependent inhibitor Sic1^24^ was modeled (Fig. 5a) following the strategy shown in Fig. 5b (for simplicity, each node was assumed to represent the four cell cycle phases: Sic1 (G1), Clb5 (S), Clb3 (G2), and Clb2 (M). This approach, which does not rely on the use of rate constants for the model reactions as compared to kinetic modeling, has been employed to implement all known regulations occurring among Clbs and Sic1 (ref. 12) as well our newly unraveled interactions. Furthermore, both activatory and inhibitory regulations were considered, to not exclude any potential direct or indirect modes that may have an impact on the formation of Clb waves. Various optimization strategies to analyze the PKN (see Supplementary Text for details) were not able to reproduce the behavior of three experimental conditions, i.e., wild type, deletion of *SIC1* and overexpression of *SIC1*, which have definite phenotypes regarding the formation of Clb waves.^12^ Possible attractors that reproduce these conditions were then generated (Supplementary Fig. S8a–S8d), by creating a set of networks with a minimum number of edges satisfying specific rules (see Supplementary Text for details on edge filtering and Table 1) corresponding to the current knowledge of the regulation among Clbs and Sic1 as well as to our novel experimental findings. Starting from the edge pool, i.e., the collection of all possible edges (640) generated previously, the possible minimal models constructed were 36. These models were simulated by using GenYsis,^25^ reproducing the Boolean attractors expected for the three experimental conditions. Subsequently, models were filtered by selecting only those that reproduced the waves of nodes during qualitative ordinary differential equation (ODE) simulations by using SQUAD.^26^ Among the 36 candidate models that were simulated, only 6 reproduced the waves observed experimentally (Supplementary Fig. S9a–S9c).

**Table 1:** Minimal model edge pool

Clb2	→	Sic1
Clb2 & Clb3	→	Sic1
^Clb2	⟞	Sic1
Sic1 & Clb2	⟞	Clb5
Sic1 & Clb3 & Clb2	⟞	Clb5
Clb5	→	Clb3
^Clb5 & Sic1	⟞	Clb3
Clb3	→	Clb2
^Clb3	⟞	Clb2
Sic1 & ^Clb3	⟞	Clb2

List of regulatory rules not contradicting any of the transitions for attractors (wild type and *SIC1* overexpression) and steady states (*sic1*Δ). This edge pool is the result of filtering the attractor candidate B (see Supplementary Fig. S8b). Three logical rules can specify the regulation of Sic1 and Clb2, and two logical rules can specify the regulation of Clb5 and Clb3. The symbol used are: ^, NOT; &, AND; →, activation; ⟞, inhibition.

In order to restrict further the plausible model candidates, the effect *CLB* overexpression was simulated and compared to the known experimental phenotypes. Clb2 can be overexpressed up to ten-fold as compared to its endogenous level without significantly reducing cell viability.^27^ When the 6 model candidates were tested, two of them (models 2 and 3) were able to reproduce the expected result; the minimal model 2 is shown in Fig. 5c and its simulations are reported in Supplementary Fig. S9d (see Supplementary Fig. S9e for simulations of the minimal model 3). A further increase in Clb2 level reaches a threshold that leads to the inhibition of mitotic exit;^27^ this is confirmed by our simulations, showing that in these two models the systems collapses when the level of Clb2 is increased upon a certain level (Fig. 5d). The same behavior was observed for the minimal model 3 (see Supplementary Fig. S9f). The structure of the minimal models 2 and 3 is compatible with a sequential, direct activation of mitotic Clb cyclins (Clb5 → Clb3 → Clb2) to produce periodic Clb oscillations that alternate to Sic1. These findings were also independently validated by applying a probabilistic Monte-Carlo approach (see Supplementary Text for details and Supplementary Fig. S10a–S10c).^28^ With this strategy, we verify that the results obtained with SQUAD are not software-specific, but can be extended to different qualitative methodologies.

Together, our analyses support the experimental findings by showing that a linear transcriptional cascade of *CLB* activation controls the sequential appearance of waves of Clb cyclins.

## Discussion

Precise order of cell cycle events is dependent on gradual changes in substrate specificity of cyclin-dependent kinases, which is mediated by phase-specific cyclins.^29–31^ Sequential activation of cyclins occurs with a characteristic staggered behavior known as waves of cyclins,^1, 2^ and oscillations in their level ensure a robust timing of cell cycle progression. For this timing to be achieved, an interplay between cyclins, cyclin/Cdk1 activities, and the transcription network has been recently deciphered.^5, 7, 32, 33^ It has been shown that the mechanism by which transcription of mitotic *CLB* cyclin genes is controlled involves Cdk activities and forkhead transcription factors (Fkhs).^4, 34^

**Fig. 6:**
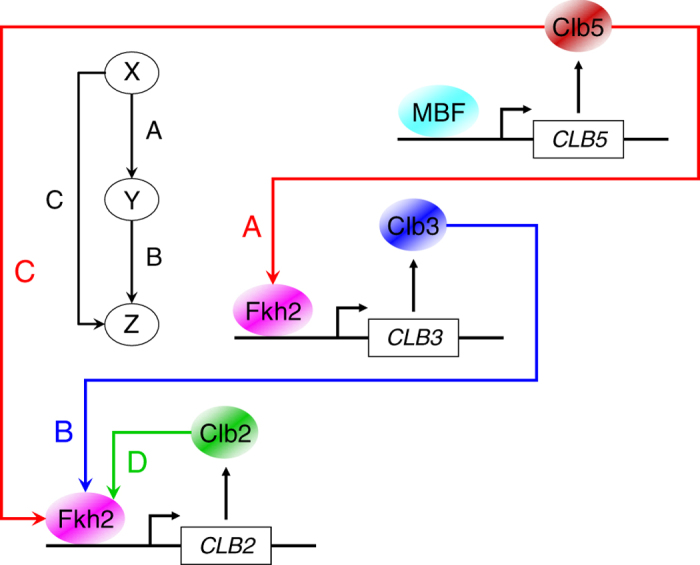
Model for the transcriptional regulation of the mitotic Clb cascade. A coherent type 1 feed-forward loop may activate *CLB* genes. Our data indicate a linear activation of Clb cyclins via the Fkh2 transcription factor: Clb5/Cdk1 promotes *CLB3* transcription (*arrow A*), Clb3/Cdk1 promotes *CLB2* transcription (*arrow B*) together with Clb5/Cdk1 (*arrow C*), and Clb2/Cdk1 promotes *CLB2* transcription by a positive feedback loop (*arrow D*). For the sake of clarity, Cdk1 subunit has been omitted. *Arrows* represent activating interactions

We shed new light on the molecular regulation at the basis of the staggered timing of mitotic Clb cyclin waves, by predicting computationally, and validating experimentally, a network motif able to drive the transcriptional activation of *CLB* genes (Fig. 6). We propose that Fkh2 is activated by sequential accumulation of phase-specific Clb/Cdk1complexes, thus providing a rationale explanation for the sequential appearance of Clb cyclins. Although Fkh2 and Fkh1 are homologous with overlapping functions, Fkh1 showed a stable interaction with at least one cyclin for each Clb subtype, i.e., Clb2, Clb3, and Clb5 (Supplementary Fig. S6a), which represent the more abundant fraction of Clb-associated Cdk1 activities.^35^ Accumulation of *CLB3* and *CLB4* transcripts is decreased in *fkh2*Δ mutants; conversely, Fkh1 deletion did not affect accumulation of *CLB3* transcript levels but led to an increase of *CLB4* transcripts in both hydroxyurea- and nocodazole-arrested cells (Supplementary Fig. S2b and Fig. 2a, respectively), suggesting a potential involvement of Fkh1 in *CLB4* gene repression during cell cycle progression. Although both Fkh1 and Fkh2 regulate *CLB3* transcription and are significantly recruited to the *CLB3* promoter, the timely onset of Clb3 protein is affected only in *fkh2*Δ but not in *fkh1*Δ cells (Fig. 2d), indicating that Fkh2 controls directly *CLB3* expression. Interestingly, the slight inhibitory effect of Fkh1 observed on *CLB3* transcription seems to be reflected on the amount of Clb3 protein accumulated but not on its temporal appearance, thus suggesting a yet unknown mechanism by which Fkh1 may modulate Clb3 protein levels. The topology that emerges from these data, in which (i) sequential waves of *CLB* cyclins transcription are synchronized by Fkh2 and (ii) mitotic Clb/Cdk1 activities modulate Fkh2 (Fig. 4b), was further validated by computer-based Boolean modeling approaches. This qualitative modeling strategy has been employed to simulate cell cycle dynamics.^36^ We identified minimal models of Clb/Cdk1 regulation that produced robust, cyclic oscillations of the mitotic Clb states, compatible with the involvement of an activator molecule—which we have shown to be the Fkh transcription factor Fkh2—in a linear Clb activation. Thus, our experimental and computational analyses pinpoint that Fkh2, major regulator of cell division, controls the temporal expression of mitotic *CLB* waves, and its activity is modulated by Clb/Cdk1 complexes throughout the cell cycle. In support of this scenario, both Clb5/Cdk1 and Clb3/Cdk1 may promote *CLB2* transcription (Fig. 4a)—either by a linear cascade (Clb5 → Clb3 → Clb2) or by a feed-forward loop in which the linear cascade has been shown here to cover a major role—by phosphorylating Fkh2^10^ (Fig. 3c, d), and their progressive activation through Fkh2 guarantees timely waves of Clb cyclins throughout cell cycle progression. Interestingly, the phosphosites S683 and T697, both located in the C‐terminal region of Fkh2, are recognized by all Clb/Cdk1 kinase activities, and their deletion leads to a reduction on the amount of Fkh2 phosphorylated^10^ (Fig. 3e).

The model of a linear activation of Clb cyclins via Fkh2 may be part of the classical coherent type 1 feed-forward loop often found in biochemical networks,^37^ and it is highly favored during the evolution of transcriptional networks in budding yeast.^14^ This regulatory mode can sustain temporal Clb oscillations by creating a delay that depends on the logical function between two inputs, Clb5/Cdk1 (X) and Clb3/Cdk1 (Y). X positively regulates Y, and both jointly regulate the common target *CLB2* (Z); strikingly, each connection (X→Y, Y→Z and X→Z) is in place by the activation of Fkh2 (Fig. 6). The coherent feed-forward loop may serve as a delay element: it responds rapidly to stimuli in one direction (from Clb2 ON to Clb5 OFF for cell cycle re-entry), and at a delay to steps in the opposite direction (from Clb2 OFF to Clb5 ON for cell cycle progression). This may allow for a rapid response and sustained oscillations.^38^ Feed-forward loops may be seen as a multistep ultrasensitivity,^39^ where small changes in the level or activity of X can be amplified at the target gene Z because of the combined action of X and Y. Of note, within this regulatory motif, a Clb3/Cdk1-mediated positive feedback loop on *CLB3* transcription (Y→Y) may contribute to timely shape certain Clb waves, without being sufficient *per se* to generate their oscillatory pattern, for which presence of the direct regulation of Clb3/Cdk1 on *CLB2* transcription (Y→Z) is required. However, it is at present not known whether *CLB2* expression is dependent on the accumulation of adequate levels or activities of both Clb5/Cdk1 and Clb3/Cdk1 in a threshold-like fashion.

In conclusion, our findings suggest a potential involvement of differential phosphorylation mechanisms^40^ mediated by various Clb/Cdk1 activities for the Fkh1/Fkh2-dependent regulation of phase-specific *CLB* genes. These details have been not yet elucidated, and are currently under investigation in our laboratory. Remarkably, although the C-terminal domain of Fkh2 (amino acids 458–862)—which is missing in Fkh1—contains the majority of Clb/Cdk1 target sites,^10^ it does interact weakly with Clb cyclins (Supplementary Fig. S5c). Contrarily, a longer fragment (amino acids 387–862) including a part of the forkhead DNA-binding domain (FKH) revealed a strong interaction with all Clb cyclins (Fig. 3b), thus suggesting that recruitment of Fkh2 is potentially mediated by this region. We speculate that a cooperativity of Clb/Cdk1-dependent phosphorylations could promote the activation of Fkh2 in order to drive timely waves of *CLB* expression. This regulatory mode coupling Clb/Cdk1 activity and transcription may fine tunes the *precise* cell cycle timing, pinpointing a design principle in cell division.

## Materials and methods

### Yeast strains and plasmids

Yeast strains, plasmids and growth conditions used in this study are described in Supplementary Materials and Methods.

### Cell synchronization, cytometry analysis, and western blot

Cell synchronization, cytometry analysis, and western blot were performed as previously described^12, 20^ with modifications as described in Supplementary Materials and Methods.

### In vitro kinase assays

For the assay in Fig. 3c, GST-Fkh1 and GST-Fkh2 were expressed in *E. coli* and purified using glutathione-Sepharose beads. To obtain the Clb3/Cdk1 complex, the W303 strain and a strain expressing Clb3-HA from its chromosomal locus were used. For the assay in Fig. 3e, Clb2/Cdk1 and Clb3/Cdk1 complexes were isolated from the W303 strain. Yeast cells were transformed with plasmids expressing C-terminal TAP-tagged Clb2 and Clb3 under galactose (GAL1) promoter (pRSAB1234GAL-CLB2-TAP and pRSAB1234GAL-CLB3-TAP). The kinase assays were performed as described in Supplementary Materials and Methods.

### In vivo phosphorylation assay

For the assay in Fig. 3d, yeast cells were grown at 25 °C in yeast extract peptone dextrose (YPD) and synchronized in G1 phase with α-factor. Samples were collected every 10 min for 90 min after synchronous release, and extracted proteins of the most relevant time points were applied to a 6% polyacrylamide gel electrophoresis (PAGE) gel added with Phos-tag (Wako) and MnCl_2_ for 2 h at 100 V in the cold. Proteins were transferred to a polyvinylidene difluoride membrane, and Fkh2-6HA was analyzed by western blot using an anti-HA antibody. The kinase assay was performed as described in Supplementary Materials and Methods.

### Protein–protein interaction assays

Yeast-two-Hybrid screen, GST pull-down and BiFC assays were performed as previously described^12, 20^ with modifications as described in Supplementary Materials and Methods.

### ChIP and real-time PCR

ChIP and quantitative, real-time PCR were performed as previously described^20^ with modifications as described in Supplementary Materials and Methods.

### Kinetic and Boolean modeling

Kinetic modeling and global sensitivity analysis were performed as previously described^12^ with modifications as described in Supplementary Materials and Methods. Boolean simulations were performed with GenYsis,^25^ which uses efficient, reduced ordered binary decision diagrams based algorithms to efficiently compute cyclic attractors. All simulations were performed using the synchronous mode. Continuous simulations were performed with SQUAD,^26^ which converts a given network into a discrete dynamical (standardized ODEs) system, and it uses a binary decision diagram algorithm to identify all the steady states of the system. Independent validation of the logical models was performed with MaBoSS,^28^ which is based on continuous time Markov processes applied on Boolean state spaces, and translates a set of ODEs on probability distributions. Boolean simulations and analysis of the networks were performed as described in Supplementary Materials and Methods.

## Electronic supplementary material

Supplementary Information

Supplementary Figures
