# Supplementary material for: A Clb/Cdk1-mediated regulation of Fkh2 synchronizes CLB expression in the budding yeast cell cycle
Source: NPJ Syst Biol Appl. 2017 Mar 6;3:7. doi: 10.1038/s41540-017-0008-1 (PMC5460246; doi:10.1038/s41540-017-0008-1)
Supplement: Supplementary file 1 — Supplementary Information [file 41540_2017_8_MOESM1_ESM.docx]

**Supplementary Information**

**A Clb/Cdk1-mediated regulation of Fkh2 synchronizes *CLB* expression in the budding yeast cell cycle**

Christian Linke, Anastasia Chasapi, Alberto González-Novo, Istabrak Al Sawad,

Silvia Tognetti, Edda Klipp, Mart Loog, Sylvia Krobitsch, Francesc Posas, Ioannis Xenarios,

Matteo Barberis

Correspondence: M Barberis ([M.Barberis@uva.nl](mailto:M.Barberis@uva.nl))

**Supplementary Text**

**Ordinary differential equations (ODEs) describing the dynamics of Clb/Cdk1 activation**

The kinetic model presented is based on a previously published model by Barberis and colleagues.^1^ The biochemical reactions of the network shown in **Figure 1a** are implemented by mass action kinetics, which describe the condensed and highly simplified transcriptional regulation processes considered. The equations are listed in the below:

**Kinetic parameters describing the dynamics of Clb/Cdk1 activation**

Kinetic parameters of the network shown in **Figure 1a**^1^ are listed in the following. Sic1 initial concentration is set to 5 in all simulations, whereas initial concentration of other components is set to zero. Concentrations are given in dimensionless units, and rate constants are given in time^-1^.

| **Parameter** | **Description of process** | **Parameter** | **Description of process** |
| --- | --- | --- | --- |
| k1 = 0.1 | Cdk1-Clb5,6 production | kA = 100 | *CLB3,4* transcription regulated by Cdk1-Clb5,6 |
| k2 = 5 | Sic1 binding to Cdk1-Clb5,6 |  |  |
| k3 = 0.5 | Cdk1-Clb5,6-Sic1 dissociation | kB = 1000 | *CLB1,2* transcription regulated by Cdk1-Clb3,4 |
| k4 = 0.01 | Clb5,6 degradation (ternary) |  |  |
| k5 = 0.05 | Sic1 degradation (Clb-regulated) | kC = 100 | *CLB1,2* transcription regulated by Cdk1-Clb5,6 |
| k6 = 0.7 | Clb5,6 degradation (binary) |  |  |
| k7 = 0.01 | Cdk1-Clb3,4 production | kD = 100 | *CLB1,2* transcription regulated by Cdk1-Clb1,2 |
| k8 = 0.7 | Clb3,4 degradation (binary) |  |  |
| k9 = 0.001 | Cdk1-Clb1,2 production |  |  |
| k10 = 0.7 | Clb1,2 degradation (binary) |  |  |
| k11 = 5 | Sic1 binding to Cdk1-Clb1,2 |  |  |
| k12 = 0.5 | Cdk1-Clb1,2-Sic1 dissociation |  |  |
| k13 = 0.01 | Clb1,2 degradation (ternary) |  |  |
| k14 = 0.05 | Sic1 degradation (Clb-regulated) |  |  |
| k15 = 5 | Sic1 binding to Cdk1-Clb3,4 |  |  |
| k16 = 0.5 | Cdk1-Clb3,4-Sic1 dissociation |  |  |
| k17 = 0.01 | Clb3,4 degradation (ternary) |  |  |
| k18 = 0.05 | Sic1 degradation (Clb-regulated) |  |  |
| k26 = 0.001 | Sic1 degradation (basal) |  |  |

**Fkh2 is not responsible of the premature Clb5 protein accumulation**

To investigate the potential role of Fkh2 removal on *CLB5* transcription, *CLB5* mRNA levels were measured in *fkh1*Δ*, fkh2*Δ or *fkh1*Δ*fkh2*Δ strains after α-factor treatment by quantitative Real-Time PCR. The *fkh2*Δ strain did not show any change in *CLB5* mRNA levels as compared to wild type (**Supplementary Figure S2f**). Contrarily, a slight increase *CLB5* mRNA levels was observed in *fkh1*Δ as well as in *fkh1*Δ*fkh2*Δ mutants, as compared to wild type (**Supplementary Figure S2f**). This result indicates that Fkh2 is not involved in *CLB5* transcription. The premature accumulation of Clb5 protein observed in *fkh2*Δ cells may be explained as follows. In absence of Fkh2, *CLB2* transcription as well translation are reduced and less Clb2/Cdk1 kinase complex is available to activate the APC machinery^2^, thus leading to a reduced degradation of Clb5 that can accumulate earlier as compared to wild type. Fkh1 may be involved in *CLB5* transcription; however, further analyses are required to clarify this process.

**A Clb3/Cdk1-mediated positive feedback loop may be involved in *CLB3* transcription, but is not sufficient to drive mitotic Clb waves**

We employed the kinetic model presented in **Figure 1a** and implemented the Clb3,4/Cdk1-mediated positive feedback loop on *CLB3,4* transcription (indicated as FL in **Supplementary Figure S3**). Subsequently, we systematically compared network structures that differ for FL presence or absence, as well as for the presence/absence of the direct regulation of Clb3/Cdk1 on *CLB2* transcription (**Figure 1a**, arrow B, *k*_B_). Our analyses reveal that the presence of FL is compatible with the occurrence of a number of waves, although not for all Clb/Cdk1 complexes. Specifically, FL may be involved in shaping the Clb5,6/Cdk1 wave (**Supplementary Figures S3o** and **S3p**), supporting the fact that Clb3/Cdk1, together with the other mitotic Clb1-4/Cdk1 complexes may contribute to abolish the Clb5/Cdk1 activity by activating APC. However, FL is per se not sufficient to explain the wave-like behaviour of Clb3,4/Cdk1, and an increase of its extent in presence of variable *k*_B_ values (**Supplementary Figures S3a** and **S3b**, **Supplementary** **Figures S3e** and **S3f**, **Supplementary** **Figures S3i** and **S3j**) or in absence of *k*_B_ (**Supplementary** **Figures S3m** and **S3n**) abolishes the oscillations of all Clb/Cdk1 complexes. In an *in vivo* scenario, increasing levels of Clb3 due to a continuous FL may be counteracted by APC rates, in order to preserve the proper temporal window in which the waves of Clb cyclins are active. The simulations revealed that, when the waves are observed, Clb1,2 is not synchronized if *k*_B_ is low, peaking at the same time than Clb3,4 (**Supplementary Figures S3p** and **S3q**, **Supplementary Figures S3k** and **S3l**). When the extent of *k*_B_ increases, waves of cyclins begin to be successive in their maximum peaks, showing the characteristic oscillatory behaviour (**Supplementary** F**igures S3g** and **S3h**, **Supplementary** **Figures** **S3c** and **S3d**). Altogether, our computational analyses do not exclude that the Clb3/Cdk1-mediated positive feedback loop on Fkh2 may be involved on *CLB3* transcription. However, in order to achieve the characteristic oscillatory pattern as well as separation of Clb maximum peaks, presence of the direct regulation of Clb3/Cdk1 on *CLB2* transcription (*k*_B_) is required. This result supports the structure of the model presented in **Figure 1a**, where the positive feedback loop on *CLB3* transcription may provide an additional, but not per se sufficient, mechanism to shape Clb waves.

**Fkh2 full-length interacts with Clb cyclins**

Fkh2 full-length and a truncated, C-terminal region of the protein (aa. 387–862, Fkh2_387_) that lacks the highly conserved FHA domain as well as of the majority of the FKH domain (**Supplementary** **Figure S5a**) were tested. The full-length protein showed a basal, strong auto-activation (**Supplementary Figure S5b**). However, treatment with 3-amino-1,2,4-triazole (3-AT), used to measure the relative strength of interactions, strongly reduced the auto-activation and allowed to detect the interaction of Fkh2 with a number of Clb cyclins among which Clb3 (**Supplementary Figure S5b**). Testing Fkh2 and Clb1-6 cyclins in the reverse combination did not lead to a conclusive result (**Supplementary Figure S5d**). Strikingly, Fkh2_387_ prevented the auto-activation, showing a clear interaction with Clb3 as well as with the other Clb cyclins (**Figure 3b**). The interactions were validated independently by a GST pull-down assay (**Supplementary Figure S5e**).

**Fkh1/Ndd1 may be substrate of Clb/Cdk1 kinase activities**

A regulation mediated by Clb/Cdk1 complexes might occur also on Fkh1/Ndd1 (**Figure 3f**, gray dotted lines), as we observed its interaction with some of the Clb cyclins in both Yeast-two-Hybrid (**Supplementary Figures S6a–S6c**) and GST pull-down (**Supplementary Figure S6d**) assays. Remarkably, cooperativity between Ndd1 and Fkh1 was predicted by computational work^3^, and we observed this specific interaction *in vitro* by Yeast-two-Hybrid (**Supplementary Figure S7a**), GST pull-down (**Supplementary Figure S7b**) assays, and *in vivo* by the BiFC method (**Supplementary Figure S7c**). Furthermore, our data indicate that the interaction between Ndd1 and Fkh1 occurs starting from the late S to G2/M phase (**Supplementary Figure S7d**). Further analyses are needed to investigate (i) whether a feedback mediated by Clb/Cdk1 complexes can occur in Fkh1 regulation, and (ii) how Fkh1/Ndd1 oscillation may correlate temporally with the transcriptional modulation of mitotic *CLB* genes.

**Prior knowledge network (PKN)-based optimization of Clb cyclin regulation**

A prior knowledge network (PKN) of the interactions among four nodes encompassing the mitotic cyclins Clb5, Clb3 and Clb2, and the cyclin-dependent inhibitor Sic1was modelled, with each node assumed to represent the four cell cycle phases: Sic1 (G1), Clb5 (S), Clb3 (G2) and Clb2 (M). For the analysis of the PKN following optimization strategies were used. Initially, different versions of the PKN of Clb cyclin regulation shown in **Figure 4a** were simulated by using GenYsis^4^, by filtering the network interactions based on their level of confidence. However, none of the simulated models was able to reproduce the behaviour of three experimental conditions: wild type, deletion of *SIC1* and overexpression of *SIC1*, which have been shown experimentally to have definite phenotypes regarding the formation of Clb waves.^1^ Specifically, wild type and *SIC1* overexpressing strains show a sequential appearance of Clb5, Clb3 and Clb2 after decrease of Sic1, and their subsequent removal from the cell when Sic1 levels increase at the end of the cell cycle. Of note, the wave-like pattern is delayed in *SIC1* overexpression. The alternating appearance/removal of activators (Clb cyclins) and inhibitor (Sic1) guarantees the overall cyclic behavior. Conversely, cyclin waves are abolished in a *sic1*Δ strain, with Clb levels reaching different plateau; in Boolean terms, this condition would be represented by the steady state, with Sic1 equal to 0, and all Clb cyclins equal to 1. In all simulations, Sic1 was constantly activated, suppressing the appearance of all Clb cyclins. For this reason, the optimization was subsequently conducted systematically, by assessing whether a network – similar to the PKN – reproducing the expected phenotypes of the three aforementioned experimental conditions can be found. For this, two parallel strategies were followed: (a) reducing the network complexity, and (b) adding interactions potentially missing to the PKN. In the first case, i.e., the network reduction, a genetic algorithm was employed starting from the initial PKN network, in order to test the existence of a reduced model that satisfies the phenotypes of the three aforementioned experimental conditions. The genetic algorithm^5^ requires a set of interactions that is used as a pool from which the model can be constructed. Additionally, a set of parameters is provided in order to optimize the network; this set corresponds to parameters values that generate (i) an attractor with all nodes oscillating in wild type and *SIC1* overexpression, and (ii) a steady state with all Clb cyclins ON in a *SIC1* deletion. After several iterations of the algorithm, no model solution was found, based on the given interactions, that fully satisfies the outcome of the three experimental conditions. In the second case, i.e., the addition of regulations potentially missing to the PKN, the inverse approach was employed. First, the collection of all possible edges of the system was identified, referred to as edge pool. The edge pool included all possible interactions involving one, two, three or four nodes acting as co-regulators (for example, A→B, A&C→B, A&C&D→B, A&B&C&D→B). The interactions include activations (→), inhibitions (⟞), and NOT (^) (for example ^A→B). For the network of four nodes (Sic1, Clb5, Clb3 and Clb2), the edge pool contains a total of 640 edges. Subsequently, all PKN+1 networks were constructed by adding one edge from the edge pool (a list of all possible interactions involving Clb5, Clb3, Clb2 and Sic1 was created, as well as an equal number of models; each model consists of the initial PKN plus one interaction of the aforementioned list, i.e., PKN+1), in order to investigate whether the phenotypes observed experimentally would derive from missing interaction(s). After the analyses, none of the simulated networks satisfies the outcome of the three experimental conditions.

**Edge filtering analysis for selection of minimal model(s) of Clb cyclin regulation**

Considering that all nodes of the network are expected to oscillate, a rule was specified that each node should have an incoming edge that leads to its activation; therefore, a minimal model should have at least four edges, one for the regulation of each node. Furthermore, auto-regulations were not allowed, as they would isolate the auto-regulated node from the system being the only incoming edge for that node. Thus, the models generated should have four edges, with one input per node, and no auto-regulations. We employed a reconstruction approach, where we initially defined the collection of possible transitions; subsequently, for a given transition, the regulatory rules that would not allow a transition to happen were removed from the edge pool, by filtering the edges that are able to reproduce the attractors (wild type and *SIC1* overexpression) and steady states (*sic1*Δ). Only one of the three candidate attractors resulted in an edge collection that was used to construct the minimal model candidates (attractor B in **Supplementary Figure S8b**). The edge filtering analysis to reduce the possible edge pool by including only the regulatory rules that do not contradict any of the node transitions is explained in the following. A simple example is used by considering the transition 0100→0110, which is present in one of the candidate wild type attractors (see attractor B in **Supplementary Figure S8b**). In this vector, the nodes are represented in the following order: Sic1–Clb5–Clb3–Clb2. The transition considered indicates the activation of Clb3, whereas the other nodes maintain their previous activation state. In order to investigate the modes of Clb3 activation, we will demonstrate the edge filtering procedure by testing the plausibility of four regulatory rules: Sic1→Clb3, ^Sic1→Clb3, Sic1&Clb5→Clb3, ^Sic1&Clb5→Clb3.

***Sic1→Clb3 and ^Sic1→Clb3 logical rules.*** The state of Clb3 at time *t*+1 is defined by the state of its regulators at time *t*. If Sic1 is the only regulator of Clb3 (Sic1→Clb3), then Clb3 will be active (1) at time *t*+1 if and only if Sic1 is active (1) at time *t*. However, this is not true, as Sic1 state at time *t* is 0; therefore, Sic1→Clb3 can not be the logical rule regulating Clb3 and it will be removed from the edge pool. ^Sic1→Clb3 can interpreted as if the absence of Sic1 (NOT Sic1) triggers Clb3 activation, meaning that Clb3 will be active (1) at time *t*+1 if and only if Sic1 is inactive (0) at time *t*. This is true, therefore the logical rule ^Sic1→Clb3 is not removed from the edge pool.

***Sic1&Clb5→Clb3 and ^Sic1&Clb5→Clb3 logical rules.*** When examining the cases of AND (&) regulatory rules, the state of all nodes included in the regulatory rule have to be taken into account. The regulation is effective if the state of all nodes included satisfies the logical rule. At time *t*, Sic1 is inactive (0) and Clb5 is active (1); in this scenario, Clb3 switches from 0 to 1 at time *t*+1. If both Sic1 and Clb5 participate in the regulation of Clb3, the only AND rule configuration that would trigger the activation of Clb3 is ^Sic1&Clb5→Clb3, with inactive Sic1 (0) and active Clb5 (1) at time *t*. This logical rule will be mainained in the edge pool. Conversely, the logical rule Sic1&Clb5→Clb3 is not true, and it will be removed from the edge pool.

Out of all possible 640 edges, only 10 did not contradict the state transitions of the candidate attractor B, whereas the other candidate attractors could not be reached with a minimal model setting (see **Table 1** for the regulatory rules specifying the minimal model edge pool), and the possible minimal models constructed were reduced to 36. In the other two cases (attractor A and attractor C), the regulatory rules that did not contradict any transition were very few and, more importantly, not all nodes were regulated being all possible incoming edges filtered out. Without all nodes being regulated, it is not possible to obtain a cyclic attractor during the simulation, thus, the attractors A and C were rejected. Subsequently, the 36 models were filtered by selecting only those that reproduced the waves of nodes during qualitative ODE simulations by using SQUAD.^6^

## Boolean model validation using continuous time Markov process

In order to independently validate our findings, the model candidates were simulated by applying Kinetic Monte-Carlo (or Gillespie algorithm) on the Boolean state space. MaBoSS describes the behavior of heterogeneous cell population by applying continuous and discrete Markov processes on Boolean networks.^7^ This software associates transition rates to each node, computes the temporal evolution of probability distributions and estimates stationary distributions. Given some initial conditions, MaBoSS produces time trajectories, and time evolution of probabilities for each network state are estimated.^7^ Since MaBoSS describes cell population rather than single cells, the probabilities of all states converge to a constant when time tends to infinity. The Boolean feedback loops depends only on the topology of the regulations and not on transition rates or time; however, such cycles cannot be linked perfectly to periodic behavior of instantaneous probabilities that MaBoSS uses, because the set of these probabilities cannot be perfectly periodic. They can display damped oscillations, or no oscillation at all. When simulating our models, damped oscillations were observed, with the probability distributions of all network states tending to a stationary distribution (**Supplementary Figure S10a**). The entropy and transition entropy measurements that MaBoSS provides can be used to characterize cyclic stationary distributions. These measurements are defined in Stoll et al., 2012: “*The* ***Entropy (H)*** *measures the disorder of the system. Maximum entropy means that all states have the same probability; H=0 means that one of the states has a probability of one*” (i.e., steady state) and “*The* ***Transition Entropy (TH)****characterizes the system at the level of a single trajectory. For each state S, there exists a set of possible transitions. TH(S)=0 if there is no transition from S to any other state.*”.^7^ To identify a cyclic stationary distribution, i.e., a stationary distribution that contains a cyclic attractor, the entropy H has to be non-zero and the transition entropy TH has to be 0. This is indeed observed when simulating our networks (**Supplementary Figure S10b**). To maintain the cyclic behavior of the probabilities, MaBoSS can simulate discrete time. **Supplementary Figure S10c** shows that the cyclic behavior of the models is clearly maintained, with a much slower decrease through time due to the stochastic events. Altogether, both continuous and discrete MaBoSS simulations validate the results obtained with GenYsis^4^ and SQUAD^6^. In the case of continuous time simulation, an attractor was retrieved with a state probability graph similar to the state transition graph obtained with GenYsis/SQUAD. In the case of discrete time simulation, probability-based that describe cell populations (instead of single cell SQUAD simulations), damped oscillation were observed where each state has similar probabilities. However, looking at the asymptotic behavior of entropy (not converging to 0) and transition entropy (converging to 0), the stationary distribution can be characterized as cyclic stationary distribution.

**References for Supplementary Text**

1. Barberis, M. *et al.* Sic1 plays a role in timing and oscillatory behaviour of B-type cyclins. *Biotechnol. Adv.* **30**, 108–130 (2012).
2. Wäsch, R. & Cross, F. R. APC-dependent proteolysis of the mitotic cyclin Clb2 is essential for mitotic exit. *Nature* **418**, 556–562 (2002).
3. Tsai, H. K., Lu, H. H. & Li, W. H. Statistical methods for identifying yeast cell cycle transcription factors. *Proc. Natl. Acad. Sci. USA* **102**, 13532–13537 (2005).
4. Garg, A., Di Cara, A., Xenarios, I., Mendoza, L. & De Micheli, G. Synchronous versus asynchronous modeling of gene regulatory networks. *Bioinformatics* **24**, 1917–1925 (2008).
5. Dorier, J., Crespo, I., Niknejad, A., Liechti, R., Ebeling, M. & Xenarios, I. Boolean regulatory network reconstruction using literature based knowledge with a genetic algorithm optimization method. *BMC Bioinformatics* **17**, 410.
6. Di Cara, A., Garg, A., De Micheli, G., Xenarios, I. & Mendoza, L. Dynamic simulation of regulatory networks using SQUAD. *BMC Bioinformatics* **8**, 462 (2007).
7. Stoll, G., Viara, E., Barillot, E. & Calzone, L. Continuous time Boolean modeling for biological signaling: application of Gillespie algorithm. *BMC Syst. Biol.* **6**, 116 (2012).

**Supplementary Figure Legends**

## Figure S1. Kinetic model variants of Clb/Cdk1 regulation and computational time courses of total Clb cyclins levels. (a–f) Simulations of the model variants were generated starting from the network in Figure 1a by varying values of *k*_A_ (reducing or increasing of 10-fold its initial level, from 100 to 10 or to 1000) (a and b), *k*_B_ (reducing or increasing of 10-fold its initial value, from 1000 to 100 or to 10000) (c and d) and *k*_C_ (reducing or increasing of 10-fold its initial value, from 100 to 10 or to 1000) (e and f). The model variants were implemented by ordinary differential equations, with the parameters used for the simulations having the same value among all variants (see Supplemental Text for the full set of equations and parameter values^1^). (g and h) Impact of transcriptional regulations on the delay between peaks of Clb cyclins. The graphs report the time delay observed between maximum levels of Clb cyclins for binary combinations between the minimal model – where Clb/Cdk1 complexes are connected via four transcriptional regulations – and two model variants, independently. (g) Time delay calculated for the left branch (only *k*_C_ active) between Clb5,6 and Clb1,2 (t_1,2_−t_5,6_). (h) Time delay calculated for the right branch (only *k*_A_ and *k*_B_ active) between Clb5,6 and Clb1,2 (t_1,2_−t_5,6_). Each parameter of the network may vary from its selected value to the same value multiplied for a random real value comprised between 0.1 and 10, as indicated on each simulation panel.

**Figure S2.** Fkh1 and Fkh2 regulate dynamics of mitotic Clb cyclins in a cell cycle- dependent manner. (**a**) Quantitative Real-Time PCR of mitotic *CLB* transcripts in yeast cells treated with nocodazole (NOC). Total mRNA was isolated from arrested wild type, *fkh1*Δ, *fkh2*Δ and *fkh1*Δ*fkh2*Δ cells, and *CLB1*, *CLB2*, *CLB3* and *CLB4* mRNA levels were measured. *ACT1* and *TSA1* genes were used as negative controls, as they are not affected by cell cycle dynamics. Error bars on the histograms represent SDs from the mean of three independent experiments; p-values are indicated on the histograms. Nocodazole-arrested cells show that *fkh2*Δ affects both *CLB3* and *CLB4* transcript accumulation, whereas *fkh1*Δ affects *CLB4* transcript accumulation. (**b**) Quantitative Real-Time PCR of mitotic *CLB* transcripts in yeast cells treated with hydroxyurea (HU). Total mRNA was isolated from arrested wild type, *fkh1*Δ, *fkh2*Δ and *fkh1*Δ*fkh2*Δ cells, and *CLB1*, *CLB2*, *CLB3* and *CLB4* mRNA levels were measured. *ACT1* and *TSA1* genes were used as negative controls, as they are not affected by cell cycle dynamics. Error bars on the histograms represent SDs from the mean of three independent experiments; p-values are indicated on the histograms. Hydroxyurea-arrested cells show that *fkh2*Δ affects *CLB3* but not *CLB4* transcript accumulation, whereas *fkh1*Δ affects *CLB4* transcript accumulation. (**c**) Binding of Fkh1 to mitotic *CLB* promoter regions. Chromatin immunoprecipitation was performed by precipitating protein/DNA complexes from cells grown in exponential phase using an anti-Myc antibody. *ACT1* and *TSA1* genes were used as negative controls, whereas *CLB1* and *CLB2* genes as positive controls. Error bars on the histograms represent SDs from the mean of three independent experiments. (**d**) RNA polymerase II occupancy at mitotic *CLB* promoters. Association of RNA polymerase II to *CLB1*, *CLB2*, *CLB3* and *CLB4* promoters was tested using an anti-RNA polymerase antibody. *ACT1* and *TSA1* genes were used as negative controls, whereas *CLB1* and *CLB2* genes as positive controls. Error bars on the histograms represent SDs from the mean of two independent experiments. (**e**) Cell synchrony of wild type, *fkh1Δ* and *fkh2Δ* strains. Yeast cells were sampled to measure DNA content by flow cytometry at the same time points analysed for the Western blot analysis. Fkh2, but not Fkh1, deletion results in a delayed cell division, as it can be observed when comparing DNA profiles of deletion and wild type strains. (**f**) Quantitative Real-Time PCR of *CLB5* transcripts in yeast cells treated with α-factor. Total mRNA was isolated from arrested wild type, *fkh1*Δ, *fkh2*Δ or *fkh1*Δ*fkh2*Δ cells, and *CLB5* mRNA levels were measured. *ACT1* and *TSA1* genes were used as negative controls. Error bars on the histograms represent SDs from the mean of three independent experiments; p-values are not indicated on the histograms, as they are not statistically significant. α-factor-arrested cells show that Fkh1, but not Fkh2, affects *CLB5* transcript accumulation.

**Figure S3.** Contribution of the Clb3,4-mediated positive feedback loop on the formation of Clb waves. (**a–q**) Simulations of the model variants were generated starting from the network in **Figure 1a** by varying values of *k*_B_ and FL between 0 and 1000, as shown on each simulation panel. The effect of an increase of FL was tested in presence of variable *k*_B_ values – high (*k*_B_ = 1000, **a–d**), medium (*k*_B_ = 100, **e–h**) or low (*k*_B_ = 10, **i–l**) – or in absence of *k*_B_ (*k*_B_ = 0, **m–q**). Computational time courses of total Clb cyclins levels are shown. The model variants were implemented by ordinary differential equations, as described in **Supplementary Figure S1**.

**Figure S4.** Time course analysis of the association between Ndd1 and Fkh2. (**a**) Haploid yeast cells expressing the C-terminal region of the Venus protein fused to the C-terminal region of Ndd1 (Ndd1-VC) were transformed with the p426 plasmid carrying the N-terminal region of the Venus protein fused to the C-terminal region of Fkh2 (VN-Fkh2) under the control of the constitutive GPD promoter. Detection of the fluorescent signal by Bimolecular Fluorescence Complementation (BiFC) as well as DAPI stained nuclei was revealed by fluorescence microscopy. (**b**) Cells co-expressing Ndd1-VC and VN-Fkh2 were synchronized in G1 phase with α-factor, and the presence of a fluorescent signal, also called BiFC signal (identified as yellow bright spots, clearly distinguishable from the fluorescence background of yeast cells) was monitored. Arrested cells were released into fresh media and samples were collected every 10 min. DNA content of samples was determined by staining with propidium iodide and FACS analysis. Absence of the BiFC signal is observed in early G1 phase (0 – 10 min). The BiFC signal indicating an interaction between Fkh2 and Ndd1 is observed starting from late G1 phase (10 – 20 min), in agreement with Fkh2 phosphorylation being detectable 10 to 20 min after α-factor release^2^, until G2/M phase (70 min), supporting the view that Fkh2 is active in this temporal window.^3^ The BiFC signals decrease progressively in late mitosis (80 – 90 min), when the cells prepare to start a new round of cell division.

**Figure S5.** Association of Fkh2 and Ndd1 with Clb cyclins. (**a**) Schematic representation of full-length Fkh1 and Fkh2 and a series of deletion constructs used in this study. The locations of the Forkhead domain (FKH) and Forkhead associated domain (FHA) are indicated. (**b–d** and **f**) Fkh2 and Ndd1 associate with Clb cyclins by GST pull-down assay. Yeast cells expressing the fusion proteins LexA-Fkh2 (pBTM-Fkh2) and AD-Clb1-6 (pACT-Clb1-6) (**b**), LexA-Fkh2_458_ (pBTM-Fkh2_458_) and AD-Clb1-6 (pACT-Clb1-6) (**c**), LexA-Clb1-6 (pBTM-Clb1-6) and AD-Fkh2 (pACT-Fkh2) (**d**), and LexA-Clb1-6 (pBTM-Clb1-6) and AD-Ndd1 (pACT-Ndd1) (**f**) were spotted onto SDII and SDIV selective media or on a membrane for detection of the β-galactosidase activity. 3-amino-1,2,4-triazole (3-AT) was used to measure the relative strength of interactions, and its concentration is indicated for each assay. (**b**) Yeast cells expressing fusion proteins LexA-Fkh2 (pBTM-Fkh2) and AD-Clb1-6 (pACT-Clb1-6) were able to growth on selective SDIV medium. As expected, no growth of yeast expressing control proteins LexA (pBTM) and AD (pACT), or LexA (pBTM) and AD-Clb1-6 (pACT-Clb1-6), negative controls in the assay, was observed on SDIV. Although growth of yeast cells on this medium was observed for a potential interaction of Fkh2 with all mitotic Clb cyclins, this is also the case for the expression of the LexA-Fkh2 negative control per se, indicating auto-activation of reporter genes, as previously observed.^4^ To decrease the non-specific growth observed for LexA-Fkh2, as well as to investigate the relative strength of interactions, 3-amino-1,2,4-triazole (3’AT) was added to the media. Yeast cells expressing fusion proteins LexA-Fkh2 and all AD-Clb cyclins are able to grow on selective 3’AT medium, however, LexA-Fkh2 still shows a basal auto-activation, leaving an uncertainty about the veridity of Fkh2/Clb interactions. (**c**) Yeast cells expressing fusion proteins LexA-Fkh2_458_ (pBTM-Fkh2_458_) and AD-Clb1-6 (pACT-Clb1-6) were able to growth on selective SDIV medium. As expected, no growth of yeast expressing the negative controls was observed on SDIV. Growth of yeast cells on this medium was observed in correspondence of the potential interaction of Fkh2 with all mitotic Clb cyclins. (**d**) Testing Fkh2 and Clb1-6 in the reverse combination, by using LexA-Clb1-6 (pBTM-Clb1-6) and AD-Fkh2 (pACT-Fkh2) also did not lead to a conclusive result, as some of the negative control show auto-activation of reporter genes and the use of 3’AT abolishes the majority of the potential Fkh2/Clb interactions. (**f**) Only yeast cells co-expressing fusion proteins LexA-Clb2 (pBTM-Clb2) or LexA-Clb3 (pBTM-Clb3) and AD-Ndd1 (pACT-Ndd1) were able to grow on selective SDIV media. No growth of yeast expressing negative control proteins (pBTM and pACT, or pBTM-Clb1,5,6 – not conclusive for pBTM-Clb4 – and pACT, or pBTM and pACT-Ndd1) was observed on SDIV medium after 3’AT was added, with the exception of the co-expression of pBTM-Clb4 and pACT. The interaction between the C-terminal domain of human ATXN2, ATXN2-FD, and the C-terminal domain of human PABP, PABC, was used as positive control. At least three independent transformations have been performed and four clones were tested in each experiment. (**e** and **g**) Fkh2 and Ndd1 associate with Clb cyclins by GST pull-down assay. Bacterial expressed proteins GST and GST-Clb1-6 were immobilized on Glutathione Sepharose beads and incubated with lysate from yeast cells expressing Myc-tagged Fkh2 (**e**) or Myc-tagged Ndd1 (**g**) from their endogenous promoters. Fkh2-Myc or Ndd1-Myc lysates served as a loading controls (lanes 1). Sepharose beads (lanes 2) and immobilized GST (lanes 3) were used as negative controls. Precipitation of Fkh2-Myc or Ndd1-Myc bound on GST-Clb1-6 (lanes 4-9) was detected with rabbit α-Myc antibody. The loading controls for GST-Clb1-6 are shown at the bottom of **Supplementary Figure S5e**. All assays have been performed three times, and one representative is shown. (**h**) Quantitative Real-Time PCR of mitotic *CLB* transcripts in yeast cells treated with nocodazole. Total mRNA was isolated from arrested wild type, *fkh2*Δ, *clb3*Δ*clb4*Δ or *clb5*Δ*clb6*Δ cells, and *CLB2* and *CLB3* mRNA levels were measured. *ACT1* and *TSA1* genes were used as negative controls, as they are not affected by cell cycle dynamics. Error bars on the histograms represent SDs from the mean of three independent experiments; p-values are indicated on the histograms. Nocodazole-arrested cells show that *clb3*Δ*clb4*Δ affects *CLB2* transcript accumulation, whereas *clb5*Δ*clb6*Δ affects both *CLB2* and *CLB3* transcript accumulation.

**Figure S6.** Association of Fkh1 with Clb cyclins. (**a–c**) For the Yeast-two-Hybrid assay, yeast cells expressing the fusion proteins LexA-Fkh1 (pBTM-Fkh1) and AD-Clb1-6 (pACT-Clb1-6) (**a**), LexA-Clb1-6 (pBTM-Clb1-6) and AD-Fkh1 (pACT-Fkh1) (**b**), and LexA-Fkh1_360_ (pBTM- Fkh1_360_) and AD-Clb1-6 (pACT-Clb1-6) (**c**) were spotted onto SDII and SDIV selective media or on a membrane for detection of the β-galactosidase activity. 3-amino-1,2,4-triazole (3-AT) was used to measure the relative strength of interactions, and its concentration is indicated for each assay. (**c**) A truncated, C-terminal region of Fkh1 (aa. 360–484, Fkh1_360_) that lacks the Forkhead associated domain (FHA, aa. 76–162) as well as the majority of the Forkhead domain (FKH, aa. 301–391), is able to interact specifically with Clb2. The interaction between the C-terminal domain of human ATXN2, ATXN2-FD, and the C-terminal domain of human PABP, PABC, was used as positive control. At least three independent transformations have been performed and four clones were tested in each experiment. (**d**) For the GST pull-down assay, bacterial expressed proteins GST and GST-Clb1-6 were immobilized on Glutathine Sepharose beads and incubated with lysate from yeast cells expressing Myc-tagged Fkh1 from its endogenous promoter. Fkh1-Myc lysate served as a loading control (lane 1). Sepharose beads (lane 2) and immobilized GST (lane 3) were used as negative controls. Precipitation of Fkh1-Myc bound on GST-Clb1-6 (lanes 4-9) was detected with rabbit α-Myc antibody. The loading controls for GST-Clb1-6 are shown at the bottom of **Supplementary Figure S5e**. All assays have been performed three times, and one representative is shown.

**Figure S7.** Ndd1 interacts with both Fkh1 and Fkh2 *in vitro* and *in vivo*. (**a**) Ndd1 associates with Fkh1 and Fkh2 by Yeast-two-Hybrid assay. Yeast cells expressing the fusion proteins LexA-Fkh1 and LexA-Fkh2 (pBTM-Fkh1 and pBTM-Fkh2) and AD-Ndd1 (pACT-Ndd1) were spotted onto SDII and SDIV selective media or on a membrane for detection of the β-galactosidase activity. 10 mM 3-amino-1,2,4-triazole (3-AT) was used to measure the relative strength of interactions. The interaction between ATXN2-FD and PABC was used as positive control. At least three independent transformations have been performed and four clones were tested in each experiment. (**b**) Ndd1 associates with Fkh1 and Fkh2 by GST pull-down assay. Bacterial expressed proteins GST, GST-Fkh1 and GST-Fkh2 were immobilized on Glutathione Sepharose beads and incubated with lysate from yeast cells expressing Myc-tagged Ndd1 from its endogenous promoter. Ndd1-Myc lysate served as a loading control (lane 1). Sepharose beads (lane 2) and immobilized GST (lane 3) were used as negative controls. Precipitation of Ndd1-Myc bound on GST-Fkh1 and on GST-Fkh2 (lanes 4-5) was detected with rabbit α-Myc antibody. The loading controls for GST-Fkh1-2 are shown at the bottom of the panel. All assays have been performed three times, and one representative is shown. (**c** and **d**) Ndd1 associates with Fkh1 and Fkh2 by Bimolecular Fluorescent Complementation (BiFC). (**c**) Haploid yeast cells expressing the C-terminal region of the Venus protein fused to the C-terminal region of Ndd1 (Ndd1-VC) were transformed with the p426 plasmid carrying the N-terminal region of the Venus protein fused to the C-terminal region of Fkh2 (VN-Fkh2) or Fkh1 (VN-Fkh1), respectively. Cells transformed with the plasmid p426-VN were used as control (top panel). Detection of the fluorescent (BiFC) signal as well as DAPI stained nuclei was revealed by fluorescence microscopy. The BiFC signal was observed for both Ndd1/Fkh2 (mid panel) and Ndd1/Fkh1 (bottom panel) pairs, highlighting a nuclear localization of the fluorescent signal. (**d**) Time course analysis of the association between Ndd1 and Fkh1. Yeast cells co-expressing Venus fusion proteins Ndd1-VC and VN-Fkh1 were synchronized in G1 phase with α-factor, and the presence of the BiFC signal was monitored. Arrested cells were released into fresh media and samples were collected every 10 min. DNA content of samples was determined by staining with propidium iodide and FACS analysis. Absence of the BiFC signal was observed from G1 to mid-S phase (0 – 30 min), whereas its presence indicating an interaction between Fkh1 and Ndd1 was observed starting from late-S to G2/M phase (40 –70 min), supporting the view of Fkh1 being present in this temporal window.^3^ The BiFC signals decrease progressively in late mitosis (80 – 90 min), when the cells prepare to start a new round of cell division.

**Figure S8.** Expected oscillations in the Clb cyclin network and Boolean attractor candidates. (**a**) Qualitative representation of expression waves for Sic1 (black), Clb5 (red), Clb3 (blue) and Clb2 (green) in wild type cells (upper panel). Clb cyclins appear and disappear sequentially, one after the other one, alternating their presence with Sic1 oscillations. The “Booleanized” version of the expression waves (bottom panel) allows to retrieve the Boolean attractor by setting activity thresholds. Each state between any activity transition is translated into a Boolean vector. (**b**) Attractor candidates (A, B, C) expected for wild type, with Sic1, Clb5, Clb3 and Clb2 displaying alternating oscillatory states. (**c**) Attractor candidates (A, B, C) expected for *SIC1* overexpression, with Clb5, Clb3 and Clb2 displaying alternating oscillatory states. As compared to wild type, the state of Sic1 is constantly set to 1 to mimic its overexpression. (**d**) Attractor candidate (A) expected for *sic1*Δ, with Clb5, Clb3 and Clb2 displaying steady states (Boolean value equal to 1). As compared to wild type, the state of Sic1 is constantly set to 0 to mimic its deletion.

**Figure S9.** Minimal candidate models reproducing experimental Clb and Sic1 waves. (**a**) Six minimal models that satisfy the known experimental conditions (wild type, *SIC1* overexpression and *sic1*Δ) by both Boolean simulations (GenYsis) and standardized ordinary differential equation (ODE) simulations (SQUAD). Arrows and circles denote activations and inhibitions, respectively. A number of interactions appear in more than one model, with Sic1&Clb2⟞Clb5 being the only interaction in common among all models. (**b** and **d–f**) SQUAD simulations of the simplest minimal model candidate (model 1) (**b**), the minimal model 2 (**d**) and the minimal model 3 (**e**). From top to bottom are plotted simulations of wild type (upper panel), *SIC1* overexpression (mid panel) and *sic1*Δ (bottom panel). It shall be noted that by the computational time t=15 wild type cells have entered the fourth cycle, whereas *SIC1* overexpressed cells show a delay as compared to wild type, having just completed the third cycle. The results are in agreement with the experimental observations^1^. (**c**) Boolean wild type attractor (B) for the simplest minimal model candidate (model 1). (**f**) SQUAD simulations of *CLB2* overexpression experiments for the minimal model 3. From top to bottom are plotted simulations of an increase dosage of Clb2: 1 (upper panel), 5 (mid panel) and 30 (bottom panel). The insert in the bottom panel represents a magnification of Sic1, Clb5 and Clb3 oscillations when Clb2 is set to 30. It shall be noted that by the computational time t=15 the cycles are progressively reduced when increasing the Clb2 level. The results are in agreement with the experimental observations.^5^

**Figure S10.** Validation of Boolean models output by continuous time Markov process. (**a**) Standard continuous Markov process simulation applying a Kinetic Monte-Carlo (or Gillespie algorithm) approach by using MaBoSS. The probability distributions for network states tend to stationary distribution; however, the sequence of states retrieved during the first cell cycle corresponds to the wild type attractor obtained with GenYsis and SQUAD. (**b**) Entropy (H) and transition entropy (TH) distributions. The network simulation results in H being non-zero and TH being roughly 0, and indicates that the stationary distribution shown in panel A contains a cyclic attractor. (**c**) Discrete time simulation using MaBoSS. The cyclic behavior of the network can be clearly observed, despite the state probabilities reduce with time. The colour legend corresponds to the one used in panel A.

**References for Supplementary Figure Legends**

1. Barberis, M. *et al.* Sic1 plays a role in timing and oscillatory behaviour of B-type cyclins. *Biotechnol. Adv.* **30**, 108–130 (2012).
2. Pic-Taylor, A., Darieva, Z., Morgan, B. A. & Sharrocks, A. D. Regulation of cell cycle-specific gene expression through cyclin-dependent kinase-mediated phosphorylation of the forkhead transcription factor Fkh2p. *Mol. Cell. Biol.* **24**, 10036–10046 (2004).
3. Simon, I. *et al.* Serial regulation of transcriptional regulators in the yeast cell cycle. *Cell* **106**, 697–708 (2001).
4. Linke, C., Klipp, E., Lehrach, H., Barberis, M. & Krobitsch, S. Fkh1 and Fkh2 associate with Sir2 to control CLB2 transcription under normal and oxidative stress conditions. *Front. Physiol.* **4**, 173 (2013).
5. Cross, F. R., Schroeder, L., Kruse, M. & Chen, K. C. Quantitative characterization of a mitotic cyclin threshold regulating exit from mitosis. *Mol. Biol. Cell.* **16**, 2129–2138 (2005).

**Supplementary Materials and Methods**

**Yeast strains**

Yeast strains BY4741 (*MAT*a *his3Δ1 leu2Δ0 met15Δ0 ura3Δ0*) and L40ccua (*MAT*a *his3-200 trp1-901 leu2-3,112 LYS2::(lexAop)4-HIS3 ura3::(lexAop)8-lacZ ADE2::(lexAop)8-URA3 gal80 canR cyh2R*) were used to generate the variant strains used in this study (see **Supplementary Table S1**). Generally, a one-step PCR-mediated gene targeting procedure was carried out for genetic manipulations.^1^ To generate the respective gene deletion strains, the plasmid pUG6 (accession number P30114, Euroscarf) was used as template to amplify a gene-specific loxP-flanked G418 cassette. Then, the amplified DNA cassette was used for transformation.^1^ After selection of transformants and verification of the correct chromosomal integration of the loxP-flanked cassette, a yeast clone was transformed with the plasmid pSH47 (accession number P30119, Euroscarf) to express Cre-recombinase for excision of the integrated gene-specific loxP-flanked cassette. Subsequently, transformants were incubated on selective medium containing 1 mg/ml 5-fluoroorotic acid (Zymo Research), and grown yeast clones were analysed for uracil auxotrophy. To generate double gene deletions, a second integration cassette was amplified using plasmid pUG6 as template and used to transform the corresponding deletion strains, and processed as described above. To express Myc-tagged Fkh1, Fkh2 and Ndd1 in yeast, the plasmid pYM18 containing a 9-*MYC* sequence (accession number P30304, Euroscarf) was used as template for the amplification of the respective gene-specific integration cassettes. To perform time course experiments three isogenic BY4741 derivative strains were used, YAN49 (*CLB3-TAP:HIS3*, *SIC1-TAP:KanMX::Nat*, *CLB2-18MycKl:KanMx::URA3*, *CLB5-6HA:KanMx*), generated by tagging one of each of Clb pairs along with Sic1, and two variants, YAG20 (*CLB3-TAP:HIS3*, *SIC1-TAP:KanMX::Nat*, *CLB2-18MycKl:KanMx::URA3*, *CLB5-6HA:KanMx*, *fkh1::HPH*) and YAG21 (*CLB3-TAP:HIS3*, *SIC1-TAP:KanMX::Nat*, *CLB2-18MycKl:KanMx::URA3*, *CLB5-6HA:KanMx*, *fkh2::HPH*), obtained by inserting a cassette containing the Hygromycin B kinase gene (*HPH*) to disrupt *FKH1* and *FKH2* genes, respectively (see **Supplementary Table S1**). To perform *in vivo* phosphorylation assays six isogenic BY4741 derivative strains were used, YAG135 (*FKH2-6HA-hphNTI*, *bar1::LEU2*), YST44 (*FKH2-6HA-hph*, *bar1::LEU2*, *clb3::TRP1*), YST45 (*FKH2-6H-hph*, *bar1::LEU2*, *clb4::HIS3*), YST46 (*FKH2-6HA-hph*, *bar1::LEU2*, *clb3::TRP1*, *clb4::HIS3*), YST47 (*FKH2-6HA-hph*, *bar1::LEU2*, *clb2::URA3*) and YST50 (*FKH2-6HA-hph*, *bar1::LEU2*, *clb2::URA3, clb3::TRP1*, *clb4::HIS3*), generated by tagging Fkh2 (see **Supplementary Table S1**). To perform the Bimolecular Fluorescence Complementation analyses (see below) the plasmid pFA6a-Venus-C (accession number EF210810)^2^ was used to generate the Ndd1-Venus-C fusion cassette, and the plasmid pYM30 (accession number P30242, Euroscarf) was used to generate Clb1-CFP, Clb2-CFP, Clb3-CFP and Clb4-CFP integration cassettes. Genetic manipulations of all strains generated in this study were validated by PCR analysis. To generate *clb3*Δ*clb4*Δ (*MATa his3Δ1 leu2Δ0 met15Δ0 ura3Δ0 clb3::LEU2 clb4::KanMX6*) and *clb5*Δ*clb6*Δ (*MATa his3Δ1 leu2Δ0 met15Δ0 ura3Δ0 clb5::KanMX6 clb6::LEU2*) double mutants, a standard PCR-mediated gene targeting procedure was carried out as described previously.^3^ For single deletion of *CLB4* and *CLB5*, the plasmid pUG6 (accession number P30114, Euroscarf) was used as a template and the oligonucleotides Fwd/Rev-clb4Δ or Fwd/Rev-clb5Δ to amplify the gene-specific *KanMX6* integration cassettes. Then, the amplified DNA cassettes were used to transform wild type cells.^1^ After selection of transformants and verification of the correct chromosomal integration of the selection marker cassette, a respective yeast clone was used to generate double gene deletions. For the amplification of *CLB3*- and *CLB6*-specific integration cassettes, plasmid pACT4-1b^4^ and the oligonucleotide pair Fwd/Rev-clb3Δ or Fwd/Rev-clb6Δ was used to generate the respective *LEU2* integration cassette. The corresponding deletion strains were transformed and selected clones verified for positive gene disruption. Yeast strains were grown in yeast peptone dextrose (YPD) or synthetic complete (SC) media containing 2% glucose as carbon source with respective antibiotics and auxotrophic additives at 30 °C.

**Plasmids**

Plasmids pACT4-1b and pBTM117c^4^ were a kind gift from Erich Wanker (Max Delbrück Center for Molecular Medicine, Berlin, Germany). Plasmids encoding the full length fusion proteins LexA-Clb1-6 and LexA-Fkh1-2 were generated as described previously.^3,5^ The open reading frame of the respective genes was amplified using genomic DNA isolated from BY4741 strain as DNA template and oligonucleotides Fwd-clb1s, Rev-clb1n; Fwd-clb2s, Rev-clb2n; Fwd-clb3s, Rev-clb3n; Fwd-clb4s, Rev-clb4n; Fwd-clb5s, Rev-clb5n; Fwd-clb6s, Rev-clb6n and Fwd-fkh1s, Rev-fkh1n; Fwd-fkh2s, Rev-fkh2n (see **Supplementary Table S2**). For the generation of LexA-Ndd1, oligonucleotides Fwd-ndd1s and Rev-ndd1n were used. To amplify fragments of *fkh1* and *fkh2* encoding the C-terminal part of the protein Fwd-fkh1_360s, Rev-fkh1n and Fwd-fkh2_387s, Rev-fkh2n were used. The amplified DNA fragments were purified and cloned into the cloning plasmid pJET1.2/blunt (CloneJET PCR Cloning Kit, Fermentas). Subsequently, the sequence of the constructs obtained was validated by sequencing, and the plasmid DNA verified was then treated with the restriction endonucleases *Sal*I and *Not*I, purified and subcloned into the *Sal*I/*Not*I sites of pBTM117c and pACT41b. For the construction of the plasmid p426GPD-VN encoding the N-terminal region of the Venus protein, a PCR was performed using plasmid pFA6a-Venus-N (accession number EF210809)^2^ as DNA template and primer pair Fwd-venus-Nb and Rev-venus-Ne. Subsequently, the resultant DNA fragment was subcloned into the plasmid pJET1.2/blunt to generate the plasmid pJET1.2-VN. After sequence validation, the plasmid pJET1.2/VN was treated with the restriction endonucleases *Bam*HI and *Eco*RI, and the resultant DNA fragment was ligated into the *Bam*HI/*Eco*RI sites of the plasmid p426GPD.^6^ The plasmids p426GPD-VN-Fkh1, p426GPD-VN-Fkh2, p426GPD-VN-Clb1, p426GPD-VN-Clb2, p426GPD-VN-Clb3 and p426GPD-VN-Clb4 were generated to express N-terminal Venus-N-tagged Fkh1, Fkh2, Clb1, Clb2, Clb3 and Clb4. To this purpose, the open reading frame of *FKH1, FKH2,* and *CLB2* was amplified using genomic DNA as template and primer pairs Fwd-F1e, Rev-F1x; Fwd-F2e, Rev-F2x and Fwd-clb2VNe/s, Rev-clb2VNn/x (see **Supplementary Table S2**). After PCR, resultant DNA fragments were subloned into the plasmid pJET1.2/blunt. Subsequently, sequences were validated, and the respective plasmid DNA was treated with the restriction endonucleases *Eco*RI and *Xho*I. After purification, the DNA fragments were subcloned into the *Eco*RI/*Xho*I sites of the plasmid p426GPD-VN. To generate p426GPD-VN-Clb1, p426GPD-VN-Clb3 and p426GPD-VN-Clb4, the plasmid p426GPD-VN-Clb2 was treated with the restriction endonucleases *Sal*I/*Not*I and the digested DNA fragments of *CLB1, CLB3* and *CLB4* were purified and subcloned. For the GST pull-down experiments, the plasmids pGEX6p2-Clb1, pGEX6p2-Clb2, pGEX6p2-Clb3, pGEX6p2-Clb4, pGEX6p2-Clb5, pGEX6p2-Clb6 and pGEX6p2-Ndd1 encoding the open reading frame of the respective genes fused to *GST* were generated subcloning the respective DNA fragments treated with the restriction endonucleases *Sal*I and *Not*I into the *Sal*I/*Not*I sites of the plasmid pGEX6p2 (Phamarcia Biotech).

**Cell synchronization**

For the time course experiments where Clb2-18Myc, Clb3-TAP, Clb5-HA and Sic1-TAP were detected over time, yeast cultures synchronized in G1 phase were obtained by centrifugal elutriation as described previously^5^. For other synchronization experiments, overnight cultures of yeast strains were diluted to an OD_600_ ~ 0.1 – 0.2 and cultures further incubated to an OD_600_ ~ 0.6. To induce cell cycle arrest in G1 phase, cells were treated with α-factor (15 µg/ml, Universitat Pompeu Fabra, Barcelona, Spain) and further incubated for 2.5 h at 30 °C. Arrest of cells in S phase or metaphase was achieved by adding 75 mM hydroxyurea (Sigma-Aldrich) or 5 µg/ml nocodazole (AppliChem), respectively. Subsequently, cultures were incubated for additional 2 h at 30°C. In the time course experiments performed employing the Bimolecular Fluorescence Complementation analysis (see below), α-factor was added to the cultures as described above and arrested cells were harvested after incubation for 2 h at 30 °C. Cell pellets were then washed twice with the respective medium, fresh medium was added and cultures were further incubated. Samples were taken every 10 min for a total time of 120 min, and analysed by fluorescence microscopy and flow cytometry (FACS).

**Cytometry analysis**

For FACS analysis, cells were fixed in 70% ethanol and treated overnight with RNAse A (0.25 mg/ml final concentration, Sigma-Aldrich) and Proteinase K (0.5 mg/ml final concentration, Sigma-Aldrich) in 50 mM sodium citrate. DNA was stained with propidium iodide, and a total of 10,000 cells were analysed in a flow cytometer (FACSCalibur, Becton Dickinson Immunocytometry Systems, USA).

**Western blot**

For protein quantification of the time course experiments where Clb2, Clb3, Clb5 and Sic1 were detected over time, TCA protein extracts and protein detection followed the protocol described previously.^5^ For the other analyses, protein samples were loaded and separated using 10% SDS gels, transferred onto a nitrocellulose Protran membrane (PerkinElmer), and membranes were treated with mouse α-Myc tag antibody (1:10.000, Millipore) as well as the corresponding peroxidase (POD)-coupled secondary antibody (1:10.000, α-mouse IgG POD conjugate, Sigma Aldrich). Membranes were treated with Western Lighting luminol reagent (PerkinElmer) and exposed to a high performance chemiluminescence film (GE Healthcare) to visualize proteins. In addition, polyacrylamide gels were incubated in staining solution (40% Methanol, 7% Acetic acid, 0.1% Coomassie Brilliant Blue R250) to verify an equal loading of samples. De-staining of gels was performed in 40% Methanol and 10% Acetic acid.

***In vitro* kinase assays**

For the assay in **Figure 3c**, GST-Fkh2 was expressed in *E. coli* and purified using glutathione-Sepharose beads. To obtain the Clb3/Cdk1 complex, the W303 strain and a strain expressing Clb3-HA from its chromosomal locus were used. 100 ml cultures at OD_660_ ~ 0.8 were collected and total amount of protein was subjected to immunoprecipitation with anti-HA antibody and protein A-Sepharose beads. For the kinase assay, immunoprecipitated Clb3/Cdk1 was incubated with bacterially expressed GST-Fkh2 for 30 min at 30 ºC. The entire kinase assay was loaded on a gel, transferred to a membrane and exposed to detect phosphorylation. After developing the kinase assay, the membrane was subsequently plotted using antibodies against GST (substrate levels) and HA (to detect cyclin). Input levels were determined loading 5% of the total amount of protein used in the immunoprecipitation in a gel and Cdk1 was detected using anti-PSTAIR antibodies (Millipore). For the assay in **Figure 3e**, Clb2/Cdk1 and Clb3/Cdk1 complexes were isolated from the W303 strain (*MATa ade2-1 ura3-1 his3-11,15 trp1-1 leu2-3,112 can1-100*). Yeast cells were transformed with plasmids expressing C-terminal TAP-tagged Clb2 and Clb3 under galactose (GAL1) promoter (pRSAB1234GAL-CLB2-TAP and pRSAB1234Gal-CLB3-TAP). Transformants were grown in selective medium supplemented with 2% galactose to log phase (OD ~ 0.7), harvested by centrifugation and TAP-purification of cyclin/Cdk1 complexes was performed with IgG beads (Roche) as described previously.^7,8^ Cks1 was purified as described previously.^9^ For isolation of GST-tagged full-length and mutated variants of Fkh2 (pGEX-Fkh2, pGEX-Fkh2_S683A and pGEX-Fkh2_S697A), the respective bacterial protein lysates were incubated with Glutathione Sepharose 4B beads (GE Healthcare) for 8 h at 4 °C as described in the GST Pull-Down Assay section. Then, the beads were washed twice with 1 × PBS and incubated with 300 µl of elution buffer (10 Mm reduced gluthathione, 50 mM TrisHCl, pH 7,4) for 10 min. Samples of eluted proteins were separated by 10% SDS-PAGE and gels stained with Coomassie brilliant blue G-250 (Sigma). The respective protein band intensities were quantified by ImageQuant TL software (GE Healthcare) and protein concentrations determined using variable amounts of bovine serum albumine (BSA) as standard. The kinase reactions were performed according to the standard phosphorylation assay protocol.^10^ About 10 nM of the purified kinase complex was used. The assay mixture contained 50 mM HEPES pH7.4, 5 mM MgCl2, 150 mM NaCl, 0.1% NP-40, 20 mM imidazole, 2% glycerol, 2 mM EGTA, 0.2 mg/ml BSA, 500 nM Cks1 and 500 mM ATP (with added [c-32P]ATP (Perkin Elmer)). Substrate-cyclin/Cdk1 complex formation was initiated by adding pre-incubation mixture (50 mM HEPES pH7.4, 5 mM MgCl2, 150 mM NaCl, 0.2 mg/ml BSA and 500 mM ATP) and [c-32P]ATP to an excess amount of purified substrate (200 – 400 nM). Aliquots were taken after 8 and 16 min of incubation and reaction was stopped by adding SDS sample buffer (200 mM TrisHCl pH 6.8, 400 mM DTT, 8% SDS, 0,4% bromophenol blue, 40% glycerol). In a similar assay composition, the Histone H1 was used as a positive control. Phosphorylated substrate species were detected by a PhosphorImager (GE Healthcare). Quantification was performed with ImageQuant TL software (GE Healthcare).

***In vivo* phosphorylation assay**

Yeast cells were grown at 25 °C in YPD to OD_660_ ~ 0.4 and synchronized in G1 phase with α-factor (2 µg/ml) for 3 h, before releasing into fresh medium. Samples were collected every 10 min for 90 min after synchronous release, and extracted proteins of the most relevant time points were applied to a 6% PAGE gel added with 10 µM Phos-tag (Wako) and 20 µM MnCl_2_ for 2 h at 100 V in the cold. Proteins were transferred to a PVDF membrane for 2 h at 200 mA on ice. Fkh2-6HA was analysed by Western blot using an anti-HA antibody.

**Yeast-Two-Hybrid screen**

L40ccua cells were transformed with the respective pBTM117c and pACT4-1b plasmids as indicated. Transformants were selected on synthetic complete SDII medium lacking tryptophan and leucine. Subsequently, overnight cultures of single colonies (96 well format) were spotted on SDII and SDIV selection media, the latter lacking tryptophan, leucine, histidine and uracil. Reporter gene activity was reduced by adding 3-amino-1,2,4-triazole (Sigma-Aldrich) to SDIV medium in the indicated concentrations. For detection of ß-galactosidase activity, cells were spotted on SDII media covered with a nylon membrane (Magna Charge Nylon Transfer membrane, Micro Separation Inc.) and staining was performed as described previously.^11^ Plates were incubated for 5 days at 30 °C, and cell viability was monitored.

**GST pull-down assay**

Pull-down assays were performed as described previously.^5^ *E. coli* cells XL1blue (*endA1 gyrA96(nal^R^) thi-1 recA1 relA1 lac glnV44 F'[ ::Tn10 proAB^+^ lacI^q^ Δ(lacZ)M15] hsdR17(r_K_^-^ m_K_^+^)*; Stratagene) were transformed with the plasmid pGEX6p2 carrying the indicated genes and incubated in LB media. At OD_600_ of ~ 0.5 – 0.7, expression of proteins was induced by adding 1 mM isopropylbeta-D-thiogalactopyranoside (IPTG, Fermentas), and cultures were incubated for additional 3 h at 37°C. Subsequently, cells were harvested and lysed in GST-binding buffer (20 mM TrisHCl pH 7.9, 125 mM NaCl, 5 mM MgCl2, 0.5 mM DTT, 10 mg/ml Lysozym; Sigma Aldrich). Then, cell lysates were sonicated 10 times for 10 sec (Branson Sonifier W250), and 10% Glycerol and 0.1% NP-40 were added. After centrifugation (25 min, 20.000 rcf, 4 °C), Glutathione Sepharose 4B beads (GE Healthcare) were added to the supernatants containing the expressed GST-tagged proteins and incubated for 8 h at 4 °C. Then, beads were washed with GST-binding buffer, added to 1 ml yeast protein lysates (5 μg/μl total protein) that were prepared from yeast cells expressing Myc-tagged Fkh1, Fkh2 or Ndd1, and incubated overnight at 4 °C. To this purpose, 200 ml of yeast cultures (OD_600_ ~ 0.7) were harvested by centrifugation, cell pellets were washed with phosphate-buffered saline solution (PBS; 137 mM NaCl, 2.7 mM KCl, 10 mM Na2HPO4, 2 mM KH2PO4, pH 7.4), frozen in liquid nitrogen and lysed with glass beads (Sigma Aldrich, acid washed) by vigorous shaking. Finally, pull-down samples were washed twice with ice-cold GST-binding buffer and proteins bound were eluted with SDS sample buffer.

**Bimolecular Fluorescence Complementation (BiFC) assay** **and fluorescence microscopy**

Haploid yeast cells expressing the C-terminal region of the Venus protein fused to the C-terminal region of Ndd1 (Ndd1-VC) were transformed either with plasmids p426GPD-VN-Fkh1, p426GPD-VN-Fkh2, p426GPD-VN-Clb1, p426GPD-VN-Clb2, p426GPD-VN-Clb3 or p426GPD-VN-Clb4 encoding fusion proteins between the N-terminal region of Venus and the N-terminal region of the selected proteins. Subsequently, yeast clones were isolated and cultured in liquid SC media (OD_600_ ~ 0.6). Staining of the nucleus was performed by adding 2.5 µg/ml 4',6-diamidino-2-phenylindole (DAPI, Sigma-Aldrich) to the media. After 20 min, cells were harvested by centrifugation, washed once with 1 × PBS and monitored for a Venus-dependent fluorescent (BiFC) signal using a Zeiss AxioImager Z1 microscope (Carl Zeiss AG, Germany) with a Plan-NeoFluar 60 × / 1.3 NA oil immersion objective. Fluorescence images were taken using a standard fluorescein isothiocyanate filter set (excitation band pass filter, 450-490 nm; beam splitter, 510 nm; emission band pass filter, 515-565 nm), and recorded on a Zeiss Axiocam Mrm (Carl Zeiss AG) with 2 × 2 binning.

**Chromatin immunoprecipitation (ChIP)**

ChIP assays were performed essentially as described previously.^3^ Cultures of yeast cells expressing Myc-tagged Fkh1, Fkh2 or Ndd1 as well as wild type and deletion strains of *fkh1*Δ and *fkh2*Δ were grown to mid-exponential phase and cross-linked by adding formaldehyde (16% stock solution in methanol-free water, Ultra Pure EM Grade, Polysciences Inc.) to a final concentration of 1%. Cells were then harvested by centrifugation and cell pellets resuspended in pre-cooled lysis buffer (50 mM HEPES/KOH, pH 7.5, 500 mM NaCl, 1 mM EDTA, 1% Triton X-100, 0.1% DOC, 0.1% SDS, complete protease inhibitor cocktail, Roche Diagnostics GmbH). Glass beads (acid-washed, 425-600 µm in diameter, Sigma-Aldrich) were added to the samples, which were then vortexed 3 times for 90 sec. Subsequently, the soluble protein-DNA fraction was sonicated 3 times for 10 sec (Branson Sonifier W250). For the immunoprecipitation, 5 µg of mouse α-Myc tag antibody (Millipore) were added to cell lysates, which were then incubated on a rotation wheel for additional for 2 h at 4°C. To immobilize the immune complex, 50 µl of pre-cooled Protein A/G agarose mix in 1 × PBS (50% mix of Protein A/G agarose, immobilized protein, Roche) were added to the lysates, which were further incubated for 4 h at 4°C. Beads were then washed twice with lysis buffer, once with DOC buffer (10 mM Tris-Cl, pH 8, 250 mM LiCl, 0.5% NP-40, 0.5% DOC, 1 mM EDTA, pH 8) and twice with 1 × TE (Tris-Cl 10 mM, EDTA 1 mM, pH 8). Finally, immunoprecipitated complexes were eluted by adding TES buffer (Tris-Cl 50 mM, EDTA 10 mM, 1% SDS pH 8). Reverse cross-linking was performed by incubating samples overnight at 65 °C. Then, samples were treated with 0.2 µg/ml RNase A (Sigma-Aldrich) for 2 h at room temperature and 0.2 µg/ml Proteinase K (Sigma-Aldrich) was added prior incubation for 2 h at 55 °C. Extraction of DNA was performed using phenol:chloroform:isoamyl alcohol (25:24:1, Sigma-Aldrich) and precipitated with ethanol supplemented with 5 M NaCl and 1 µl LPA (Linear PolyAcrylamide, GenElute-LPA, stock: 25 mg/ml, Sigma-Aldrich, Germany). Precipitated DNA was analysed by quantitative Real-Time PCR using *CLB* promoter-specific oligonucleotides.

**Real-Time PCR**

Total RNA was isolated from yeast cells using the RiboPure Yeast Kit (Applied Biosystems, Ambion, Inc., USA) according to the manufacturer’s instructions. RNA was converted to cDNA using the SuperScript II Double-Stranded cDNA Synthesis Kit (Invitrogen, USA) according to the manufacturer’s instructions. The quantification of PCR products was performed using the fluorescent dye SYBR Green (Applied Biosystems) and a Real-Time PCR machine (Applied Biosystems, 7900 HT Real-Time PCR System). Analysis of ChIP samples was performed amplifying promotor regions of genes *CLB1*: Fwd-clb1_prom, Rev-clb1_prom; *CLB2*: Fwd-clb2_prom, Rev-clb2_prom; *CLB3*: Fwd-clb3_prom, Rev-clb3_prom and *CLB4*: Fwd-clb4_prom, Rev-clb4_prom. Oligonucleotides for open reading frames Fwd-clb1_orf, Rev-clb1_orf; Fwd-clb2_orf, Rev-clb2_orf; Fwd-clb3_orf, Rev-clb3_orf and Fwd-clb4_orf, Rev-clb4_orf were used to analyse transcription of *CLB1*, *CLB2*, *CLB3* and *CLB4* genes. *TSA1* (Fwd-tsa1_orf, Rev-tsa1_orf) and *ACT1* (Fwd-tsa1_orf, Rev-tsa1_orf) were used as a reference. All oligonucleotide sequences are listed in see **Supplementary Table S2**.

**Kinetic modeling and global sensitivity analysis**

The kinetic models of Clb/Cdk1 regulation were generated starting from the network described previously.^5^ After basal, constant production of Clb5,6/Cdk1 (*k*_1_), Sic1 binds to it forming the Clb5,6/Cdk1-Sic1 ternary complex (*k*_2_), which is also dissociated (*k*_3_). When Sic1 is degraded first by Cln1,2/Cdk1 (*k*_5_) and secondarily by all Clb/Cdk1 complexes, Clb5,6/Cdk1, Clb3,4/Cdk1 and Clb1,2/Cdk1, (*k*_5_), Clb5,6/Cdk1 promote Clb3,4/Cdk1 activation (*k*_A_), in addition to its basal, constant production (*k*_7_). Clb5,6 in the Clb5,6/Cdk1-Sic1 ternary complex are also degraded (*k*_4_). The role of Cln1,2/Cdk1 on Sic1 degradation is not included explicitly, but is incorporated as a basal activity in the reaction rate *k*_5_. Subsequently, Clb3,4/Cdk1 promote Clb1,2/Cdk1 activation (*k*_B_) together with Clb5,6/Cdk1 (*k*_C_), in addition to its basal, constant production (*k*_9_). Moreover, Clb1,2/Cdk1 promote their own activation by a positive feedback loop (*k*_D_). The basal degradation of Clb5,6, Clb3,4, Clb1,2 and Sic1 (*k*_6_, *k*_8_, *k*_10_ and *k*_26_, respectively) is also considered. The network incorporates explicitly regulation of Clb/Cdk1 production (*k*_1_, *k*_7_, *k*_9_), lumping together the processes from gene transcription to complex formation into a single step. The variants of the kinetic model were implemented by ordinary differential equations, and all parameters used for the simulations have the same value within all networks (see **Supplementary Text** for details on the full set of equations and parameters^5^). To analyse the influence of parameter values on the time delay between the peaks of Clb cyclins, a global sensitivity analysis was performed with a Monte Carlo approach. Random parameter sampling was employed to estimate the sensitivities of the network with respect to parameters, without knowing their precise values. We randomly selected 10,000 parameter sets and simulated in each case different variants of the network. In the analysis, all parameters of the network may vary between 0.1-fold and 10-fold of their default values. The simulations were run with the software Mathematica^®^ Version 10, Wolfram Research.

**Boolean simulations using GenYsis**

Boolean simulations and *in silico* gene perturbations were performed with GenYsis^12^, a toolbox that uses efficient, reduced ordered binary decision diagrams based algorithms for attractor identification and gene perturbations. Reduced ordered binary decision diagrams (ROBDDs or in short BDDs) are directed acyclic graphs that can represent large Boolean functions in a space efficient manner, and are computationally suitable for complex Boolean operations (e.g., logical AND, OR, etc.) and set operations (e.g., Union, Intersection, etc.). To map gene regulatory networks on BDDs, the regulatory relations of the network have to be transformed into Boolean functions, representing the dynamics of a model. All the operations that can be performed on Boolean functions can also be performed on their corresponding BDD representations.^12^ GenYsis uses this strategy to efficiently compute cyclic attractors of large networks that are not feasible using other existing software. The toolbox can be used for synchronous and asynchronous simulations, as well as single or multiple *in silico* perturbations. The available perturbation experiments comprise deletion, overexpression and fixed initial state experiments. All simulations were performed using the synchronous mode, and several perturbations were tested (i.e., deletion and overexpression of the cyclin-dependent kinase inhibitor Sic1 or overexpression of the mitotic cyclin Clb2). The binaries of the software are available for Linux and Mac OS X in the following address: http://www.vital-it.ch/software/genYsis

**Qualitative dynamic simulations using SQUAD**

Continuous simulations of Clb sequential expression were performed with SQUAD^13^, a software providing dynamical simulations of signaling networks that uses a standardized qualitative dynamical systems approach. SQUAD converts a given network into a discrete dynamical system, and it uses a binary decision diagram algorithm to identify all the steady states of the system. The algorithm used is based on the same principles as GenYsis, therefore providing comparable results. After identification of an attractor or a steady state, SQUAD creates a continuous dynamical system and localizes its steady states that are located near the steady states of the discrete system. The software permits to simulate the continuous system by using a system of standardized ODEs to simulate the behavior of the network in time, allowing for the modification of several parameters such as decay rate of each component. Additionally, SQUAD includes a framework for perturbing networks in a manner similar to GenYsis, thus allowing to observe the effect of gene perturbations in a dynamic fashion.^13^ The software is publicly available at http://www.vital-it.ch/software/SQUAD/

**Boolean model validation using MaBoSS**

In order to cross-verify the results obtained using GenYsis and SQUAD, an independent software called MaBoSS was used.^14^ MaBoSS is a framework for qualitative modeling that runs continuous simulations in time. Similarly to SQUAD, MaBoSS aims to bridge the gap between qualitative and quantitative approaches, and it is based on continuous time Markov processes applied on Boolean state spaces. MaBoSS uses a generalization of Boolean equations, in order to describe the temporal evolution of the modeled biological process. Mathematically, this approach can be translated in a set of ordinary differential equations (ODEs) on probability distributions. The software is able to simulate such a system by applying Kinetic Monte-Carlo algorithm on the Boolean state space, and it can thereafter compute the temporal evolution of probability distributions and estimate stationary distributions.^14^ MaBoSS can be downloaded at https://maboss.curie.fr/

**References for Supplementary Materials and Methods**

1. Longtine, M. S. *et al.* Additional modules for versatile and economical PCR-based gene deletion and modification in Saccharomyces cerevisiae. *Yeast* **14**, 953–961 (1998).
2. Sung, M. K. & Huh, W. K. Bimolecular fluorescence complementation analysis system for in vivo detection of protein-protein interaction in Saccharomyces cerevisiae. *Yeast* **24**, 767–775 (2007).
3. Linke, C., Klipp, E., Lehrach, H., Barberis, M. & Krobitsch, S. Fkh1 and Fkh2 associate with Sir2 to control CLB2 transcription under normal and oxidative stress conditions. *Front. Physiol.* **4**, 173 (2013).
4. Stelzl, U. *et al.* A human protein-protein interaction network: a resource for annotating the proteome. *Cell* **122**, 957–968 (2005).
5. Barberis, M. *et al.* Sic1 plays a role in timing and oscillatory behaviour of B-type cyclins. *Biotechnol. Adv.* **30**, 108–130 (2012).
6. Mumberg, D., Müller, R. & Funk, M. Yeast vectors for the controlled expression of heterologous proteins in different genetic backgrounds. *Gene* **156**, 119–122 (1995).
7. Puig, O. *et al.* The tandem affinity purification (TAP) method: a general procedure of protein complex purification. *Methods* **24**, 218–229 (2001).
8. Ubersax, J. A. *et al.* Targets of the cyclin-dependent kinase Cdk1. *Nature* **425**, 859–864 (2003).
9. Reynard, G. J., Reynolds, W., Verma, R. & Deshaies, R. J. Cks1 is required for G1 cyclin-cyclin-dependent kinase activity in budding yeast. *Mol. Cell. Biol.* **20**, 5858–5864 (2000).
10. Kõivomägi, M. *et al.* Cascades of multisite phosphorylation control Sic1 destruction at the onset of S phase. *Nature* **480**, 128–131 (2011).
11. Ralser, M. *et al.* Ataxin-2 and huntingtin interact with endophilin-A complexes to function in plastin-associated pathways. *Hum. Mol. Genet.* **14**, 2893–2909 (2005).
12. Garg, A., Di Cara, A., Xenarios, I., Mendoza, L. & De Micheli, G. Synchronous versus asynchronous modeling of gene regulatory networks. *Bioinformatics* **24**, 1917–1925 (2008).
13. Di Cara, A., Garg, A., De Micheli, G., Xenarios, I. & Mendoza, L. Dynamic simulation of regulatory networks using SQUAD. *BMC Bioinformatics* **8**, 462 (2007).
14. Stoll, G., Viara, E., Barillot, E. & Calzone, L. Continuous time Boolean modeling for biological signaling: application of Gillespie algorithm. *BMC Syst. Biol.* **6**, 116 (2012).

**Supplementary Tables**

**Table S1. Yeast strains used in this study.**

| Strain Genotype Source |
| --- |

L40ccua *MAT***a** *his3*_200 *trp1*-901 *leu2*-3,112 Ralser et al, 2005

*LYS2*::(lexAop)4-*HIS3 ura3*::(lexAop)8-lacZ

*ADE2*::(lexAop)8-*URA3 gal80* canR cyh2R

BY4741 *MAT***a** *his3Δ1 leu2Δ0 met15Δ0 ura3Δ0* Euroscarf

W303 *MATa ade2-1 ura3-1 his3-11,15 trp1-1 leu2-3,112 can1-100* Euroscarf

YAN49 *CLB3-TAP:HIS3*, *SIC1-TAP:KanMX::Nat* Barberis et al, 2012 *CLB2-18MycKl:KanMx::URA3*, *CLB5-6HA:KanMx*

YAG20 *CLB3-TAP:HIS3*, *SIC1-TAP:KanMX::Nat* This study *CLB2-18MycKl:KanMx::URA3*, *CLB5-6HA:KanMx fkh1::HPH*

YAG21 *CLB3-TAP:HIS3*, *SIC1-TAP:KanMX::Nat* This study *CLB2-18MycKl:KanMx::URA3*, *CLB5-6HA:KanMx fkh2::HPH*

YAG135 *FKH2-6HA-hphNTI, bar1::LEU2* This study

YST44 *FKH2-6HA-hph, bar1::LEU2, clb3::TRP1* This study

YST45 *FKH2-6H-hph, bar1::LEU2, clb4::HIS3* This study

YST46 *FKH2-6HA-hph, bar1::LEU2, clb3::TRP1, clb4::HIS3* This study

YST47 *FKH2-6HA-hph, bar1::LEU2, clb2::URA3* This study

YST50 *FKH2-6HA-hph, bar1::LEU2, clb2::URA3, clb3::TRP1* This study *clb4::HIS3*

Fkh1-Myc *MAT***a** *FKH1-MYC9*::*kanMX6* Linke et al, 2013

Fkh2-Myc *MAT***a** *FKH2-MYC9*::*natNT2* Linke et al, 2013

Ndd1-Myc *MAT***a** *NDD1-MYC9*::*kanMX6* This study

*fkh1*Δ *MAT***a** *fkh1*:: Linke et al, 2013

*fkh2*Δ *MAT***a** *fkh2*:: Linke et al, 2013

*fkh1*Δ *fkh2*Δ *MAT***a** *fkh1*:: *fkh2*:: Linke et al, 2013

Ndd1-VC/VN *MAT***a** *NDD1-VC*::*his3MX6* p426GPDpr-*VN* This study

Ndd1-VC/VN-Fkh1 *MAT***a** *NDD1-VC*::*his3MX6* p426GPDpr-*VN-FKH1* This study

Ndd1-VC/VN-Fkh2 *MAT***a** *NDD1-VC*::*his3MX6* p426GPDpr-*VN-FKH2* This study

Ndd1-VC/VN-Clb1 *MAT***a** *NDD1-VC*::*his3MX6* p426GPDpr-*VN-CLB1* This study

Ndd1-VC/VN-Clb2 *MAT***a** *NDD1-VC*::*his3MX6* p426GPDpr-*VN-CLB2* This study

Ndd1-VC/VN-Clb3 *MAT***a** *NDD1-VC*::*his3MX6* p426GPDpr-*VN-CLB3* This study

Ndd1-VC/VN-Clb4 *MAT***a** *NDD1-VC*::*his3MX6* p426GPDpr-*VN-CLB4* This study

Ndd1-VC/VN-Fkh2 *MAT***a** *NDD1-VC*::*his3MX6 CLB2-CFP*::*kanMX6*

Clb2-CFP p426GPDpr-*VN-FKH2* This study

Ndd1-VC/VN-Fkh2 *MAT***a** *NDD1-VC*::*his3MX6 CLB3-CFP*::*kanMX6*

Clb3-CFP p426GPDpr-*VN-FKH2* This study

*clb3*Δ*clb4*Δ *MATa his3Δ1 leu2Δ0 met15Δ0 ura3Δ0 clb3::LEU2* This study *clb4::KanMX6*

*clb5*Δ*clb6*Δ *MATa his3Δ1 leu2Δ0 met15Δ0 ura3Δ0 clb5::KanMX6* This study *clb6::LEU2*

|  |
| --- |

**Table S2. Oligonucleotides used in this study.**

| Primer Sequence |
| --- |

Fwd-clb1s 5’-GCTTGTCGACTAATCTTCTCATAATG-3’

Rev-clb1n 5’-ATTGCGGCCGCTTCACTCATGCAATG-3’

Fwd-clb2s 5’-CAGTCGACATTGATCTTATAGATGTCC-3’

Rev-clb2n 5’-ATTGCGGCCGCTTCTCATTCATGCAAGG-3’

Fwd-clb3s 5’-CTGAGTCGACAATGCATCATAACTCAC-3’

Rev-clb3n 5’-TATGCGGCCGCTTTAGTTAGATCTTTC-3’

Fwd-clb4s 5’-GATAGTCGACACAGATGATGCTTGAAG-3’

Rev-clb4n 5’-GAAGCGGCCGCAAGATGAGTAAGTTAG-3’

Fwd-clb5s 5’-GTAAGTCGACAACAATGGGAGAGAAC-3’

Rev-clb5n 5’-GTAGCGGCCGCATTACTAGTACTAATC-3’

Fwd-clb6s 5’-GCATGTCGACTAAAATGAATTGTATC-3’

Rev-clb6n 5’-TATGCGGCCGCTGATCTATGTTTCAAC-3’

Fwd-fkh1s 5’-GTCAGTCGACTATGTCTGTTACCAGTAG-3’

Fwd-fkh1_360s 5’-TATTGTCGACCTTCGAGAAGGTGCC-3’

Rev-fkh1n 5’-AATGCGGCCGCTGAATTTCAACTCAG-3’

Fwd-fkh2s 5’-TGAAGTCGACAATGTCCAGCAGCAAT-3’

Fwd-fkh2_387s 5’-TACTGTCGACCATTAGGCATAATTTATC-3’

Rev-fkh2n 5’-ATTGCGGCCGCTTAGTTGTTGATAATAC-3’

Fwd-ndd1s 5’-AGATGTCGACTATGGACAGAGATATAAG-3’

Rev-ndd1n 5’-TAAGCGGCCGCAAGTTTGGTTAATATTAC-3’

Fwd-venus-Nb 5’-TAGGATCCATGGTGAGCAAGGGCG-3’

Rev-venus-Ne 5’-TCGAATTCCTCGATGTTGTGGCGGAT-3’

Fwd-fkh1VNe 5’-AGAATTCTCGACTATGTCTGTTACC-3’

Rev-fkh1VNx 5’-CTCTCGAGTCAACTCAGAGAGGAATTG-3’

Fwd-fkh2VNe 5’-AGAATTCTCGACAATGTCCAGCAGC-3’

Rev-fkh2VNx 5’-GTCTCGAGTTAGTTGTTGATAATACTG-3’

Fwd-clb2VNe/s 5’-AAGAATTCAGGTCGACGATGTCCAACCCAATAG-3’

Rev-clb2VNn/x 5’-TTCTCGAGTGCGGCCGCTTCTCATTCATGC-3’

Fwd-fkh1-Myc 5’-CGTAACAACAAACGCAAACGTGAACAATTCCTCTCTGAGT

GCTAGTGGTGAACAAAAG-3’

Rev-fkh1-Myc 5’-TATTGTTTAATAATACATATGGGTTCGACGACGCTGAATT

TAGTGGATCTGATATCATCG-3’

Fwd-fkh2-Myc 5’-ACTAGATACGGATGGTGCAAAGATCAGTATTATCAACAAC

GCTAGTGGTGAACAAAAG-3’

Rev-fkh2-Myc 5’-TTCATTTCTTTAGTCTTAGTGATTCACCTTGTTTCTTGTC

TAGTGGATCTGATATCATCG-3’

Fwd-ndd1-Myc 5’-CTGTAATTCTAAATCTAATGGAAATTTATTCAATTCACAG

GCTAGTGGTGAACAAAAG-3’

Rev-ndd1-Myc 5’-TTCCATAAAAAAAAAAGGTGAGATGCAAGTTTGGTTAATA

TAGTGGATCTGATATCATCG-3’

Fwd-fkh1Δ 5’-TGTGCGTTCAATTAGCAAAGAAAGGCTTGGAGAGACACAG

GTACGCTGCAGGTCGACAAC-3’

Rev-fkh1Δ 5’-TATTGTTTAATAATACATATGGGTTCGACGACGCTGAATT

CTAGTGGATCTGATATCACC-3’

Fwd-fkh2Δ 5’-GTGCTCCCTCCGTTTCCTTTATTGAAACTTTATCAATGCG

GTACGCTGCAGGTCGACAAC-3’

Rev-fkh2Δ 5’-TTCATTTCTTTAGTCTTAGTGATTCACCTTGTTTCTTGTC

CTAGTGGATCTGATATCACC-3’

Fwd-ndd1-VN 5’-CTGTAATTCTAAATCTAATGGAAATTTATTCAATTCACAG

GGTCGACGGATCCCCGGGTT-3’

Rev-ndd1-VN 5’-TCGATTAAAAAAAAAAGGTGAGATGCAAGTTTGGTTAATA

TCGATGAATTCGAGCTCGTT-3’

Fwd-clb2-CFP 5’-GGTTAGAAAAAACGGCTATGATATAATGACCTTGCATGAA

GGAGCAGGTGCTGGTGCTGG-3’

Rev-clb2-CFP 5’-CGATTATCGTTTTAGATATTTTAAGCATCTGCCCCTCTT

CTAGTGGATCTGATATCATCG-3’

Fwd-clb3-CFP 5’-GAAGTGGATAGCATTAGCTGAACACAGAGTAGAAAGATCTAA

CGGAGCAGGTGCTGGTGCTGG-3’

Rev-clb3-CFP 5’-CTTTTTCCTTTGTTGATGCCATGTCTCGAGCTGAGGCTTT

CTAGTGGATCTGATATCATCG-3’

Fwd-tsa1_orf 5’-ATGGTCGCTCAAGTTCAAAAG-3’

Rev-tsa1_orf 5’-CGTACTTACCCTTGTATTTGTCCAA-3’

Fwd-act1_orf 5’-ATGTGTAAAGCCGGTTTTGC-3’

Rev-act1_orf 5’-TGACCCATACCGACCATGATA-3’

Fwd-clb1_orf 5’-CAGTCTAGGACGTTAGC-3’

Rev-clb1_orf 5’-GTCGTGAATAGTAGATCC-3’

Fwd-clb1_prom 5’-CAGACGCGCTTCAATTAG-3’

Rev-clb1_prom 5’-GTTACCGTTGACGTGAG-3’

Fwd-clb2_orf 5’-GGAATGTACAAGGTTGG-3’

Rev-clb2_orf 5’-CAAATTGCTGACTACTTGG-3’

Fwd-clb2_prom 5’-GTGCAAGTTCAAGGCAC-3’

Rev-clb2_prom 5’-CATGCTATGAGATGCTAG-3’

Fwd-clb3_orf 5’-GGATCGTCCAAGTACATG-3’

Rev-clb3_orf 5’-CAGCAATGAAGAGTGAG-3’

Fwd-clb3_prom 5’-GCAAGAACATGGACAC-3’

Rev-clb3_prom 5’-GTGCAACACTATTCGCATC-3’

Fwd-clb4_orf 5’-CTCTTCTACTGATGACGAAC-3’

Rev-clb4_orf 5’-CTGTCCAGCTCAGTCTG-3’

Fwd-clb4_prom 5’-CTAGAAGATTAGCAAGAT-3’

Rev-clb4_prom 5’-GAGGTTGTACCGTATACC-3’

Fwd-clb3Δ 5’-CGTTATATCAACCATCAAAGGAAGCTTTAATCTTCTCATAGG AACTGTGGGAATACTCAG-3’

Rev-clb3Δ 5’-CTTTTTCCTTTGTTGATGCCATGTCTCGAGCTGAGGCTTTCA GAAGCTTTGGACTTCTTC-3’

Fwd-clb4Δ 5’-CGGATACTAGGCTGCCCTGATCAAACAAGGAAATTGACAGGT ACGCTGCAGGTCGACAAC-3’

Rev-clb4Δ 5’-CGAAACCAAAACTGAAGCAAATGGTGTTAAGATGAGTAAGCT AGTGGATCTGATATCACC-3’

Fwd-clb5Δ 5’-TTTCCCTGTATTTAAAGCCGCTGAACACCTTTACTGAACAGT ACGCTGCAGGTCGACAAC-3’

Rev-clb5Δ 5’-GTAAAGAGTATGCGAATTCATGAGCATTACTAGTACTAATCT AGTGGATCTGATATCACC-3’

Fwd-clb6Δ 5’-TTATTCTCTGATATTCTCTCCCTCCTTTTAAATTTTTAAAGG AACTGTGGGAATACTCAG-3’

Rev-clb6Δ 5’-AGATGCAGGGGGTTAGCTGGCTATAATTTTGATCTATGTT CAGAAGCTTTGGACTTCTTC-3’

|  |
| --- |
