# Supplementary material for: A Clb/Cdk1-mediated regulation of Fkh2 synchronizes CLB expression in the budding yeast cell cycle
Source: NPJ Syst Biol Appl. 2017 Mar 6;3:7. doi: 10.1038/s41540-017-0008-1 (PMC5460246; doi:10.1038/s41540-017-0008-1)

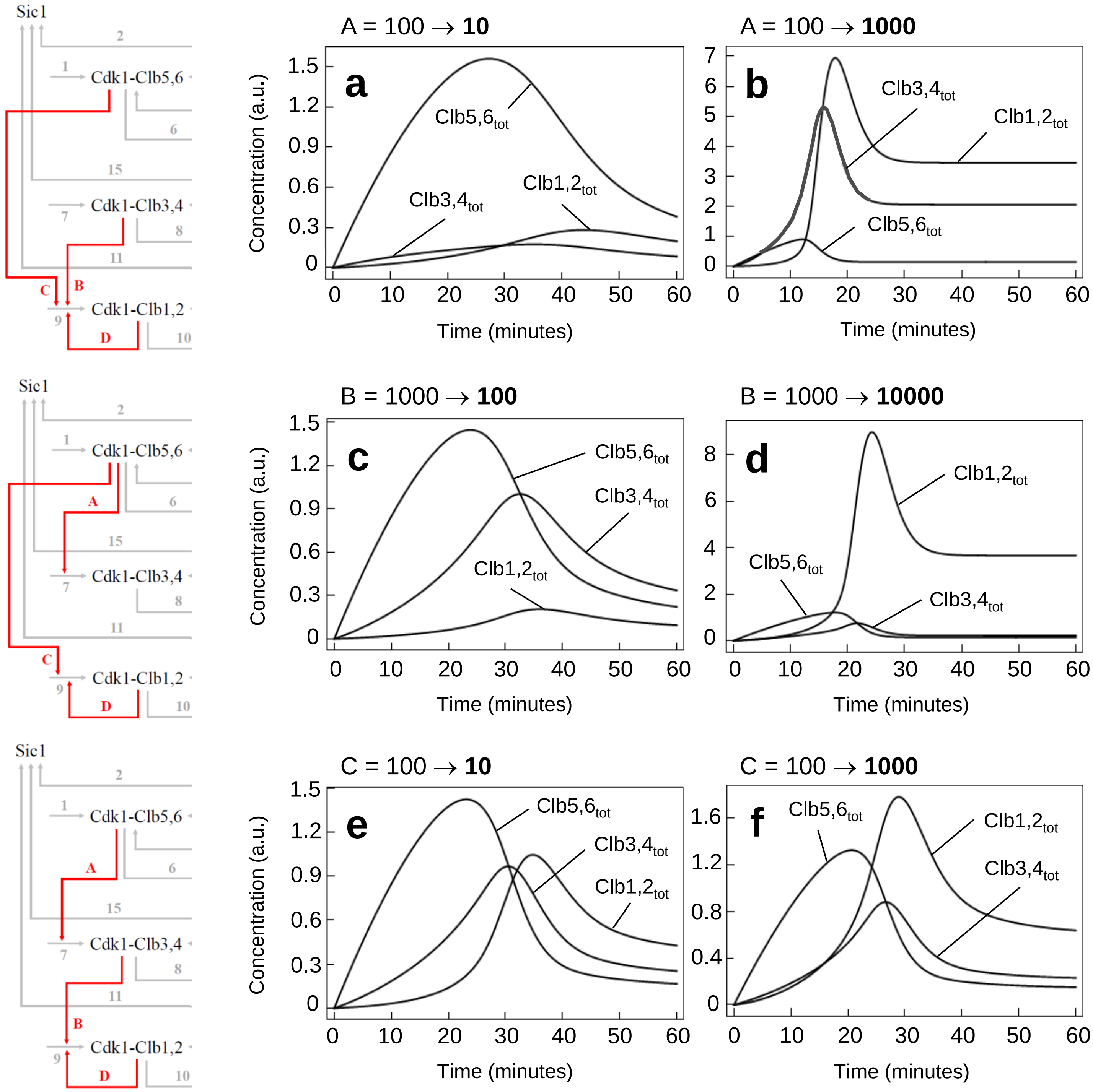

A = 100 → 10
A = 100 → 1000
B = 1000 → 100
B = 1000 → 10000
C = 100 → 10
C = 100 → 1000
a
b
c
d
e
f
Clb5,6tot
Clb3,4tot
Clb1,2tot
Concentration (a.u.)
Time (minutes)
Sic1
Cdk1-Clb5,6
Cdk1-Clb3,4
Cdk1-Clb1,2
A
B
C
D

Figure S1

Figure S1

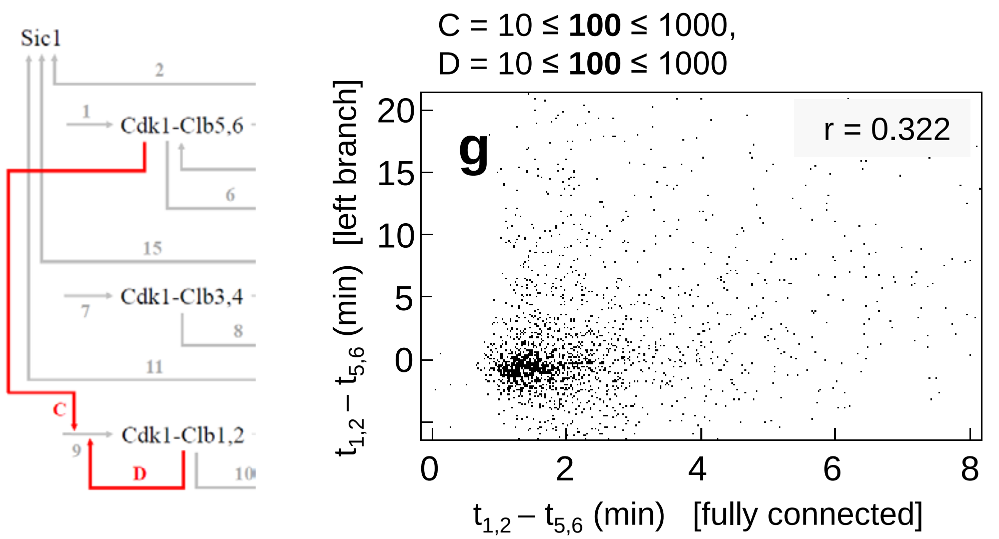

C = 10 ≤ **100** ≤ 1000,
D = 10 ≤ **100** ≤ 1000
Sic1
Cdk1-Clb5,6
Cdk1-Clb3,4
Cdk1-Clb1,2
C
D
g
r = 0.322
$t_{1,2} - t_{5,6}$ (min) [left branch]
$t_{1,2} - t_{5,6}$ (min) [fully connected]

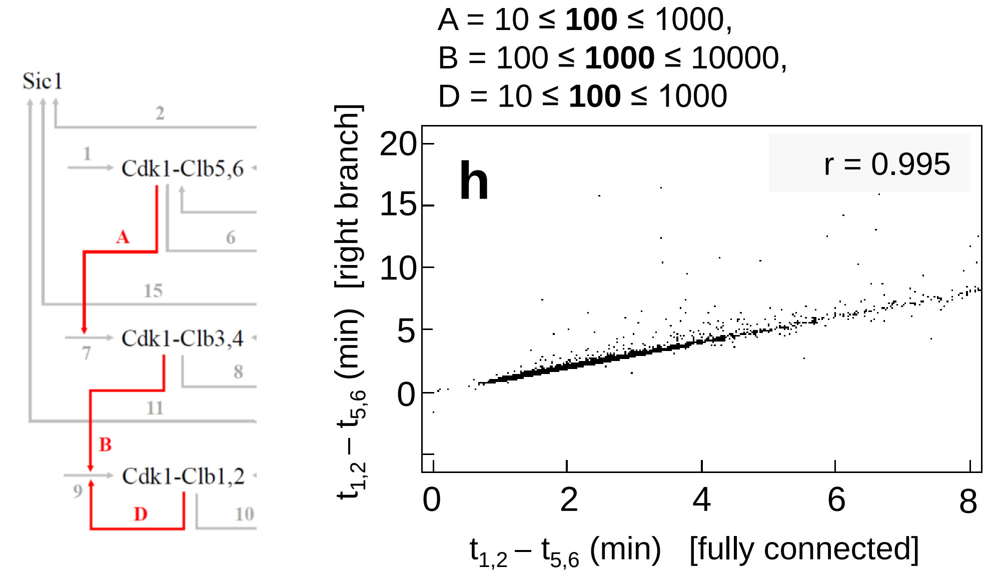

A = 10 ≤ **100** ≤ 1000,
B = 100 ≤ **1000** ≤ 10000,
D = 10 ≤ **100** ≤ 1000
Sic1
Cdk1-Clb5,6
Cdk1-Clb3,4
Cdk1-Clb1,2
A
B
D
h
r = 0.995
$t_{1,2} - t_{5,6}$ (min) [right branch]
$t_{1,2} - t_{5,6}$ (min) [fully connected]

Figure S2

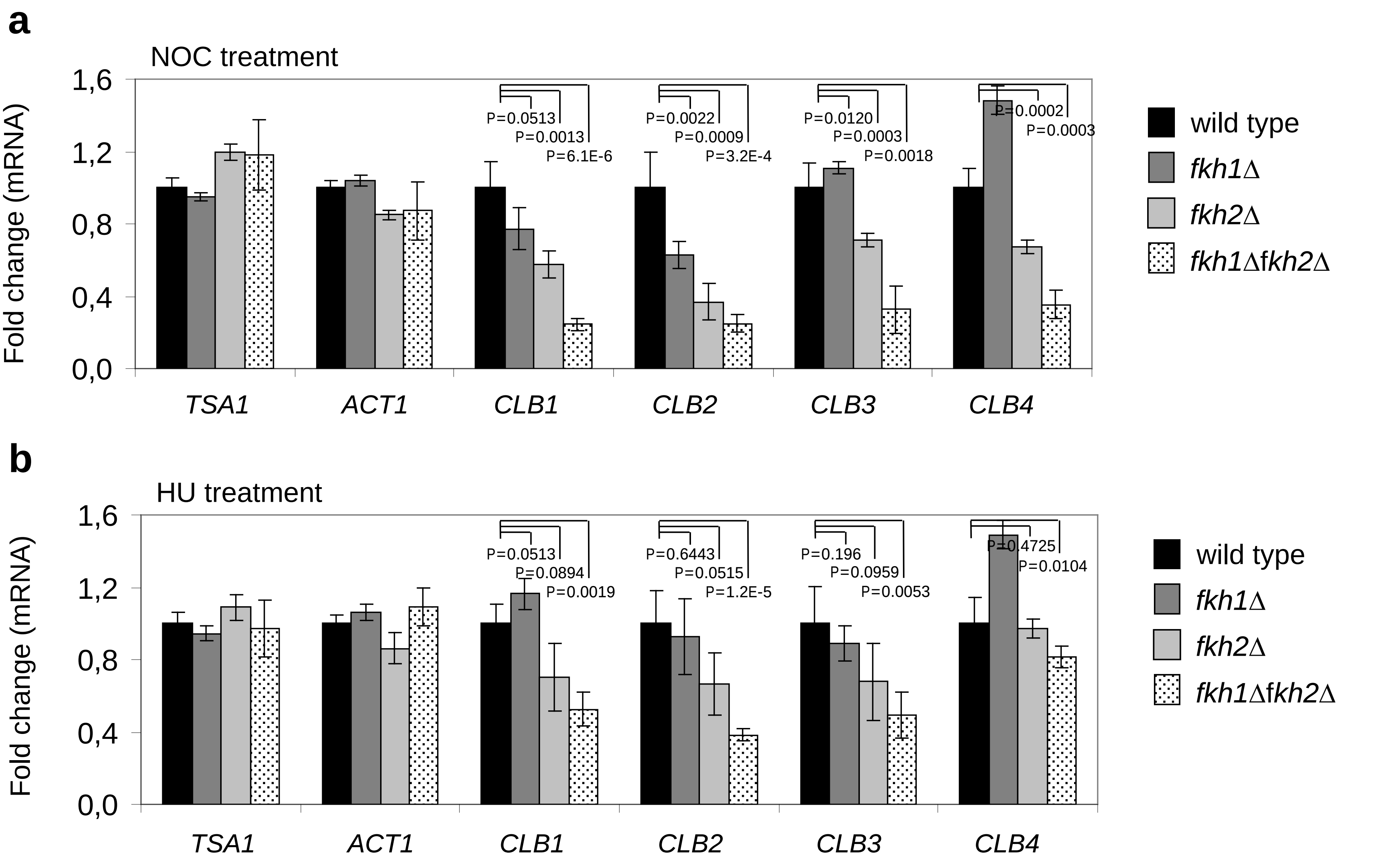
a
NOC treatment
Fold change (mRNA)
1,6
1,2
0,8
0,4
0,0
TSA1
ACT1
CLB1
CLB2
CLB3
CLB4
P=0.0513
P=0.0013
P=6.1E-6
P=0.0022
P=0.0009
P=3.2E-4
P=0.0120
P=0.0003
P=0.0018
P=0.0002
P=0.0003
wild type
fkh1Δ
fkh2Δ
fkh1Δfkh2Δ
b
HU treatment
Fold change (mRNA)
1,6
1,2
0,8
0,4
0,0
TSA1
ACT1
CLB1
CLB2
CLB3
CLB4
P=0.0513
P=0.0894
P=0.0019
P=0.6443
P=0.0515
P=1.2E-5
P=0.196
P=0.0959
P=0.0053
P=0.4725
P=0.0104
wild type
fkh1Δ
fkh2Δ
fkh1Δfkh2Δ

Figure S2

c

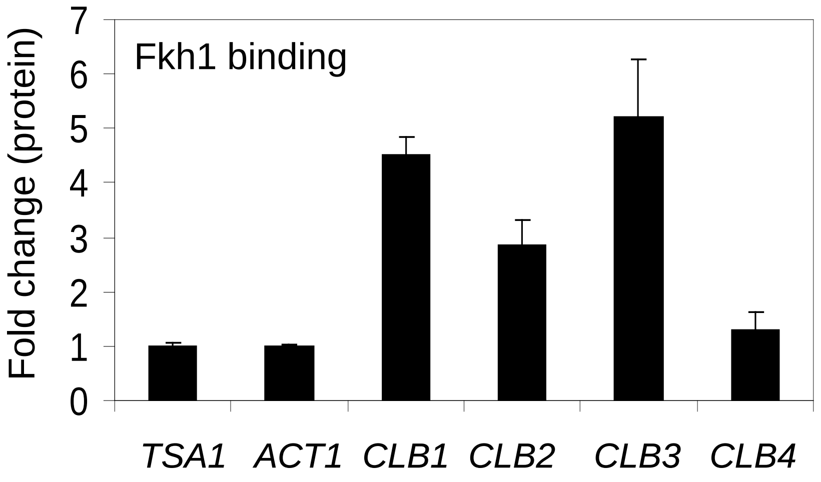
Fkh1 binding
Fold change (protein)
0
1
2
3
4
5
6
7
TSA1
ACT1
CLB1
CLB2
CLB3
CLB4

d

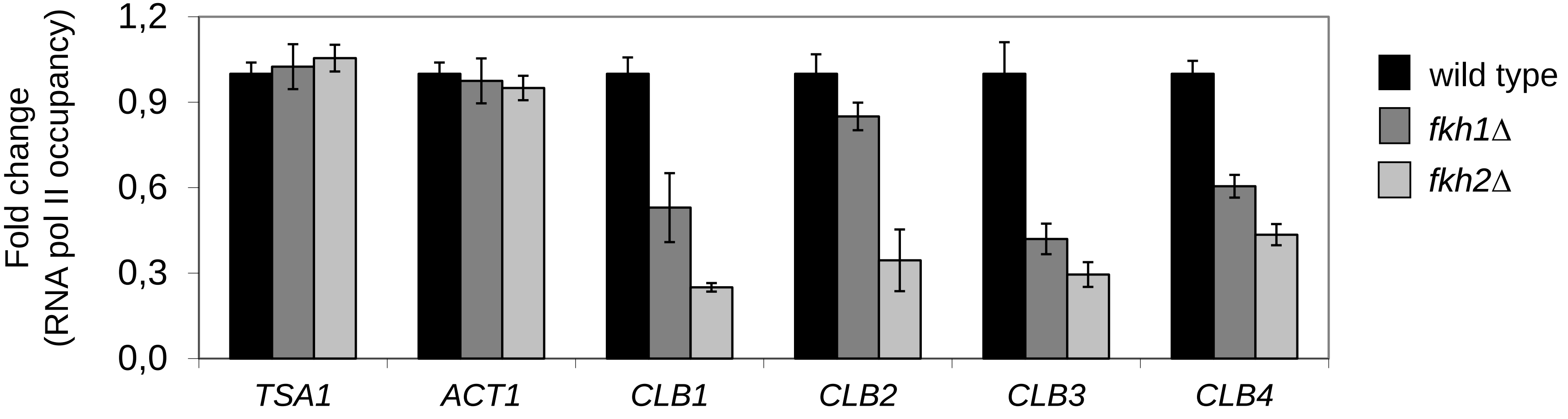
Fold change
(RNA pol II occupancy)
0,0
0,3
0,6
0,9
1,2
TSA1
ACT1
CLB1
CLB2
CLB3
CLB4
wild type
fkh1Δ
fkh2Δ

Figure S2

e

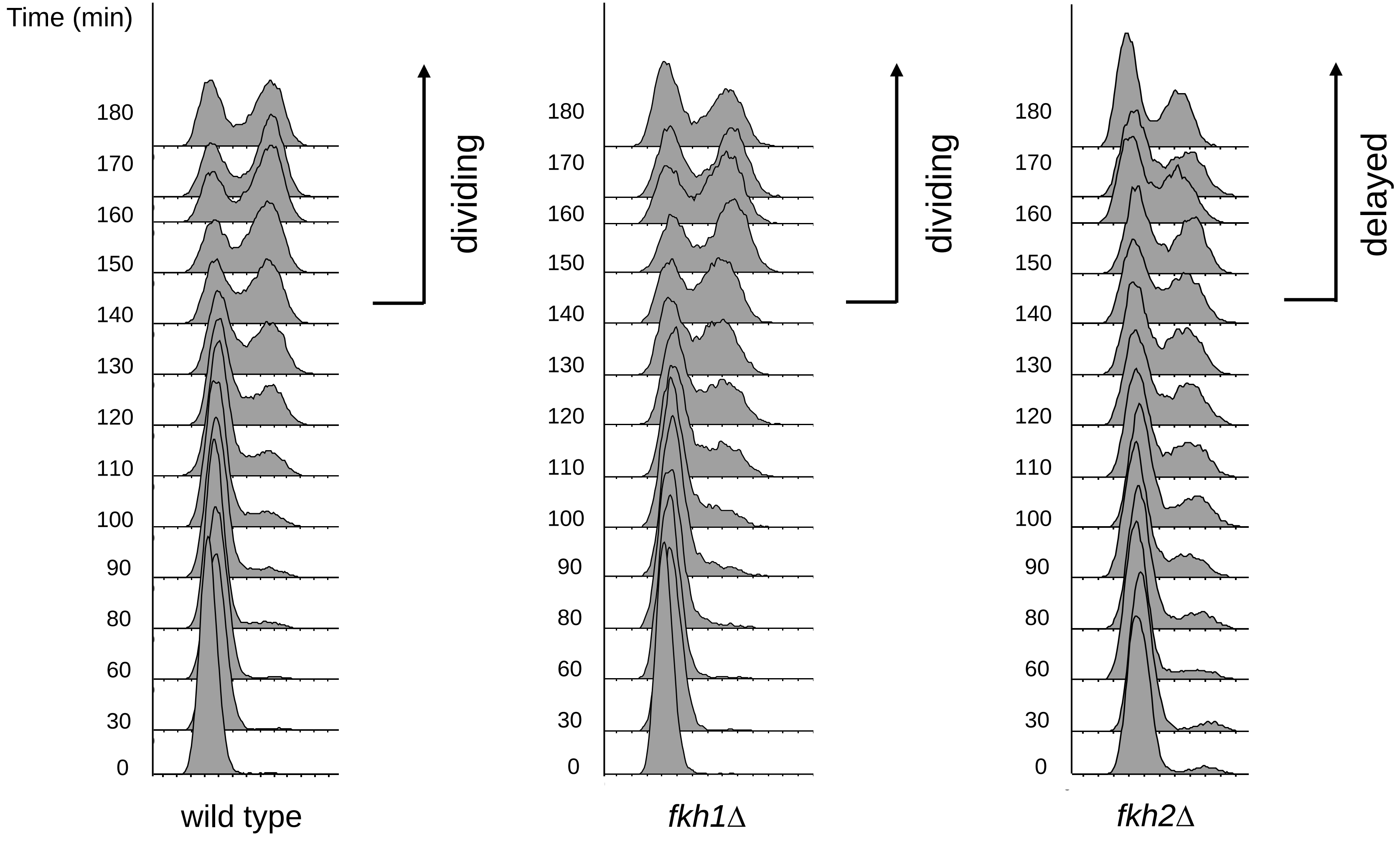

Time (min)
180
170
160
150
140
130
120
110
100
90
80
60
30
0
dividing
dividing
delayed
wild type
fkh1Δ
fkh2Δ

Figure S2

f

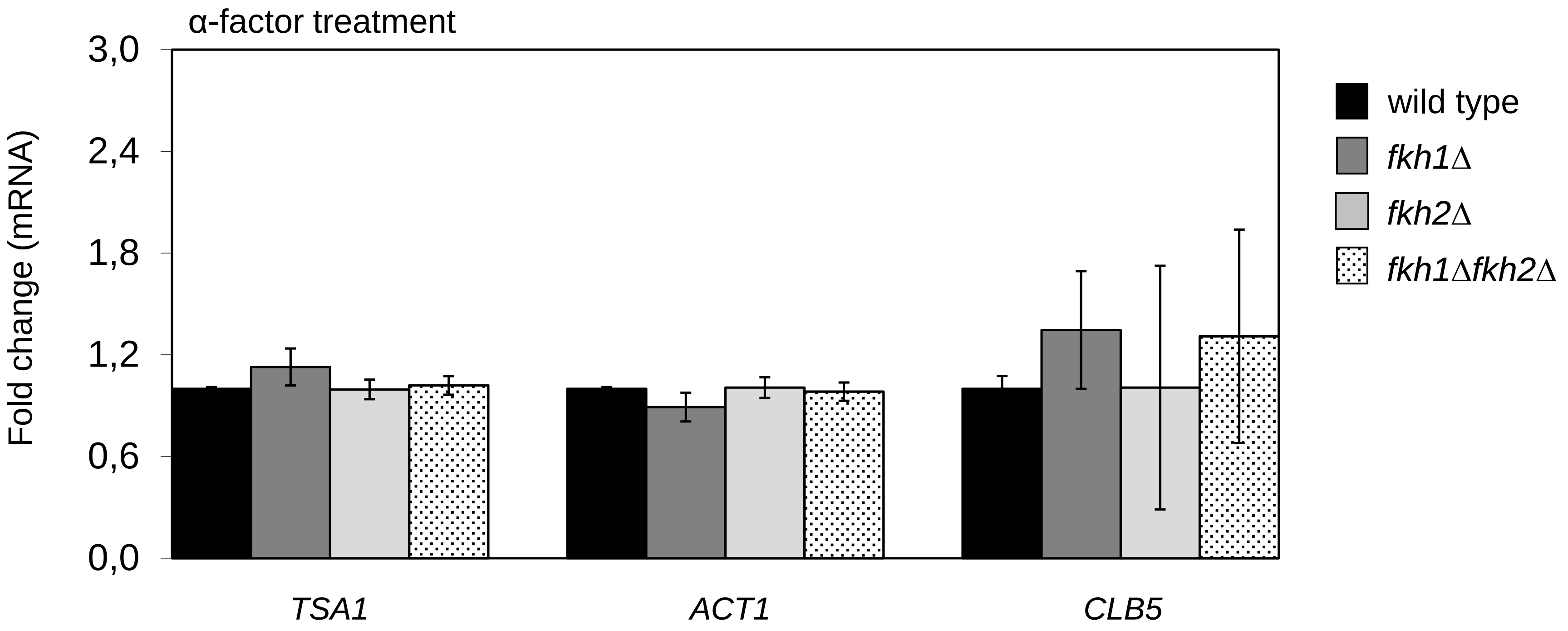
α-factor treatment
Fold change (mRNA)
3,0
2,4
1,8
1,2
0,6
0,0
TSA1
ACT1
CLB5
wild type
fkh1Δ
fkh2Δ
fkh1Δfkh2Δ

Figure S3

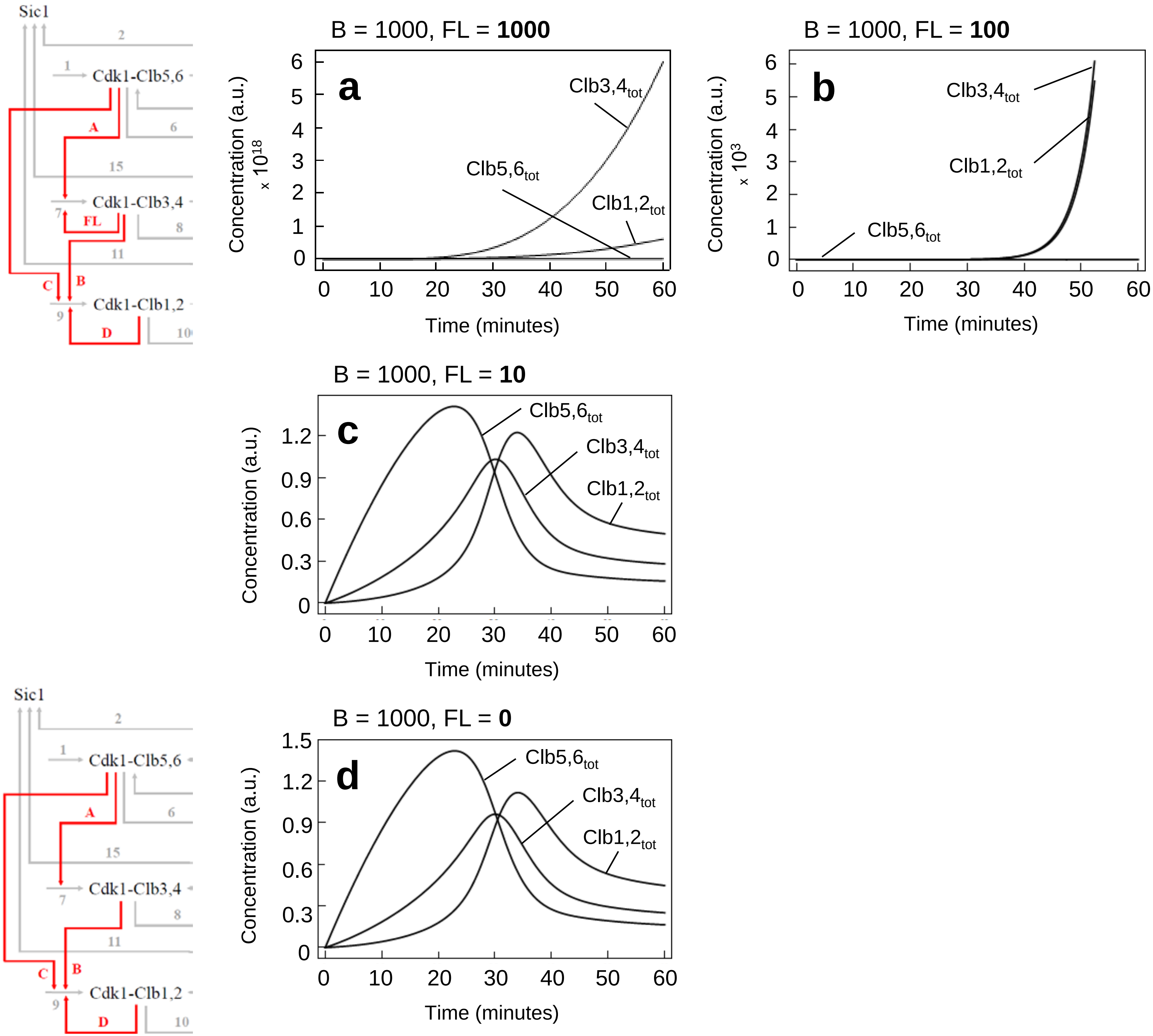

Sic1
Cdk1-Clb5,6
Cdk1-Clb3,4
Cdk1-Clb1,2
A
B
C
D
FL
B = 1000, FL = 1000
a
Clb3,4tot
Clb5,6tot
Clb1,2tot
Concentration (a.u.)
x 10^18
Time (minutes)
B = 1000, FL = 100
b
x 10^3
B = 1000, FL = 10
c
B = 1000, FL = 0
d

Figure S3

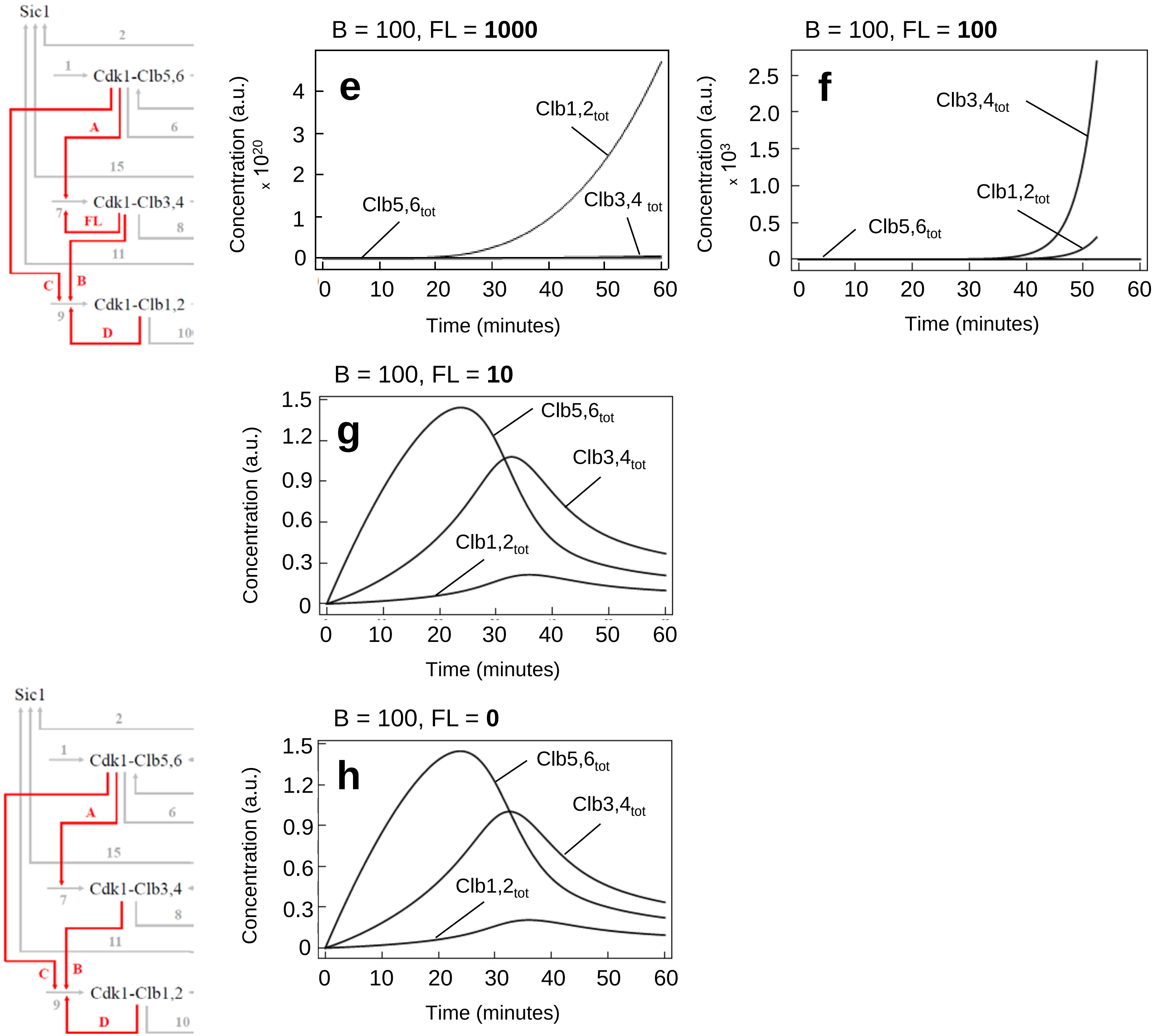

Sic1
Cdk1-Clb5,6
Cdk1-Clb3,4
Cdk1-Clb1,2
A
B
C
D
FL
B = 100, FL = 1000
e
Clb1,2tot
Clb5,6tot
Clb3,4 tot
Concentration (a.u.) x 10^20
Time (minutes)
B = 100, FL = 100
f
Clb3,4tot
Clb1,2tot
Clb5,6tot
Concentration (a.u.) x 10^3
Time (minutes)
B = 100, FL = 10
g
Clb5,6tot
Clb3,4tot
Clb1,2tot
Concentration (a.u.)
Time (minutes)
B = 100, FL = 0
h
Clb5,6tot
Clb3,4tot
Clb1,2tot
Concentration (a.u.)
Time (minutes)

Figure S3

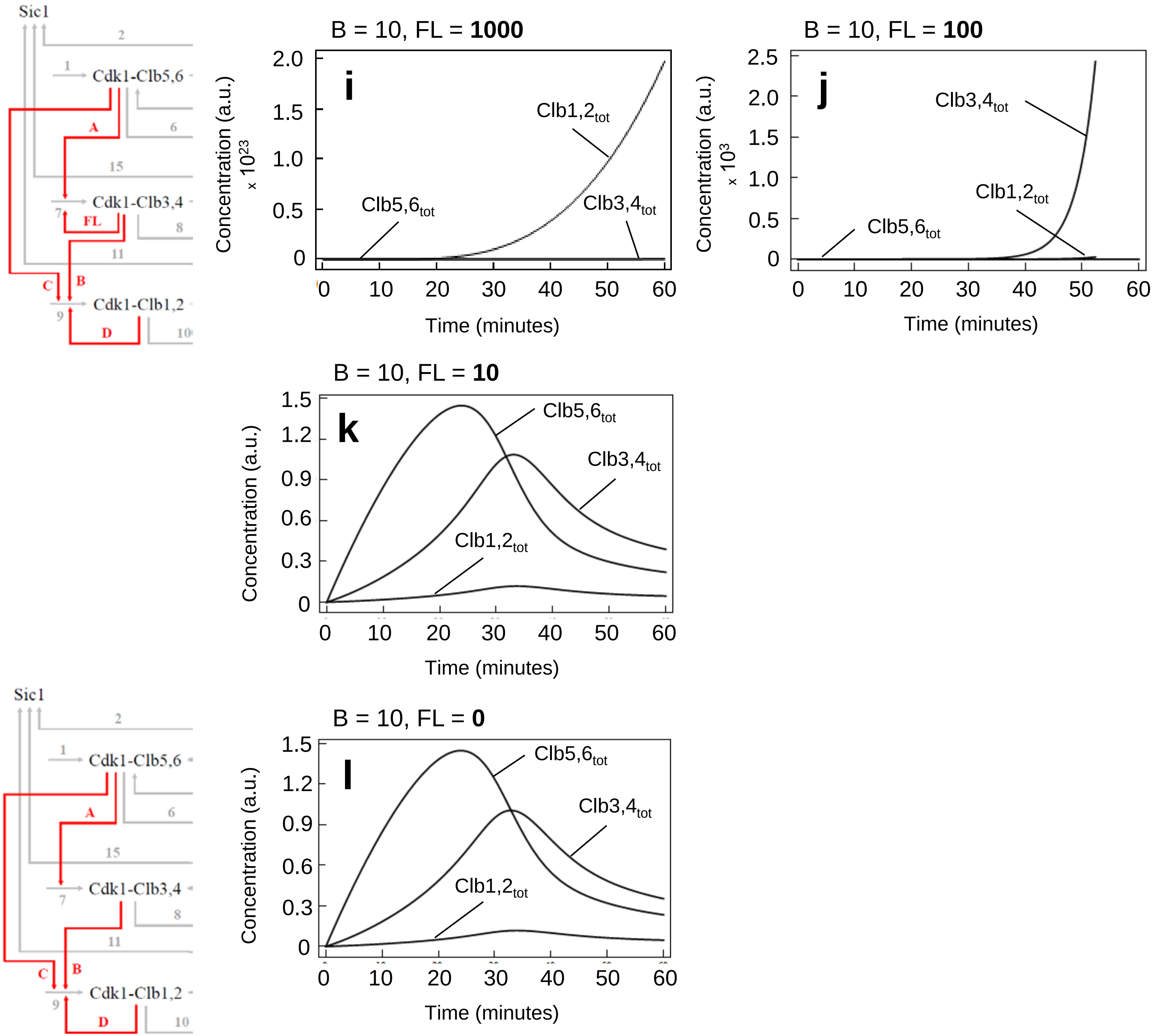

Sic1
Cdk1-Clb5,6
Cdk1-Clb3,4
Cdk1-Clb1,2
A
B
C
D
FL
1
2
6
7
8
9
10
11
15
B = 10, FL = 1000
i
Concentration (a.u.) x 10^23
Clb1,2tot
Clb5,6tot
Clb3,4tot
Time (minutes)
B = 10, FL = 100
j
Concentration (a.u.) x 10^3
Clb3,4tot
Clb1,2tot
Clb5,6tot
Time (minutes)
B = 10, FL = 10
k
Concentration (a.u.)
Clb5,6tot
Clb3,4tot
Clb1,2tot
Time (minutes)
B = 10, FL = 0
l
Concentration (a.u.)
Clb5,6tot
Clb3,4tot
Clb1,2tot
Time (minutes)

Figure S3

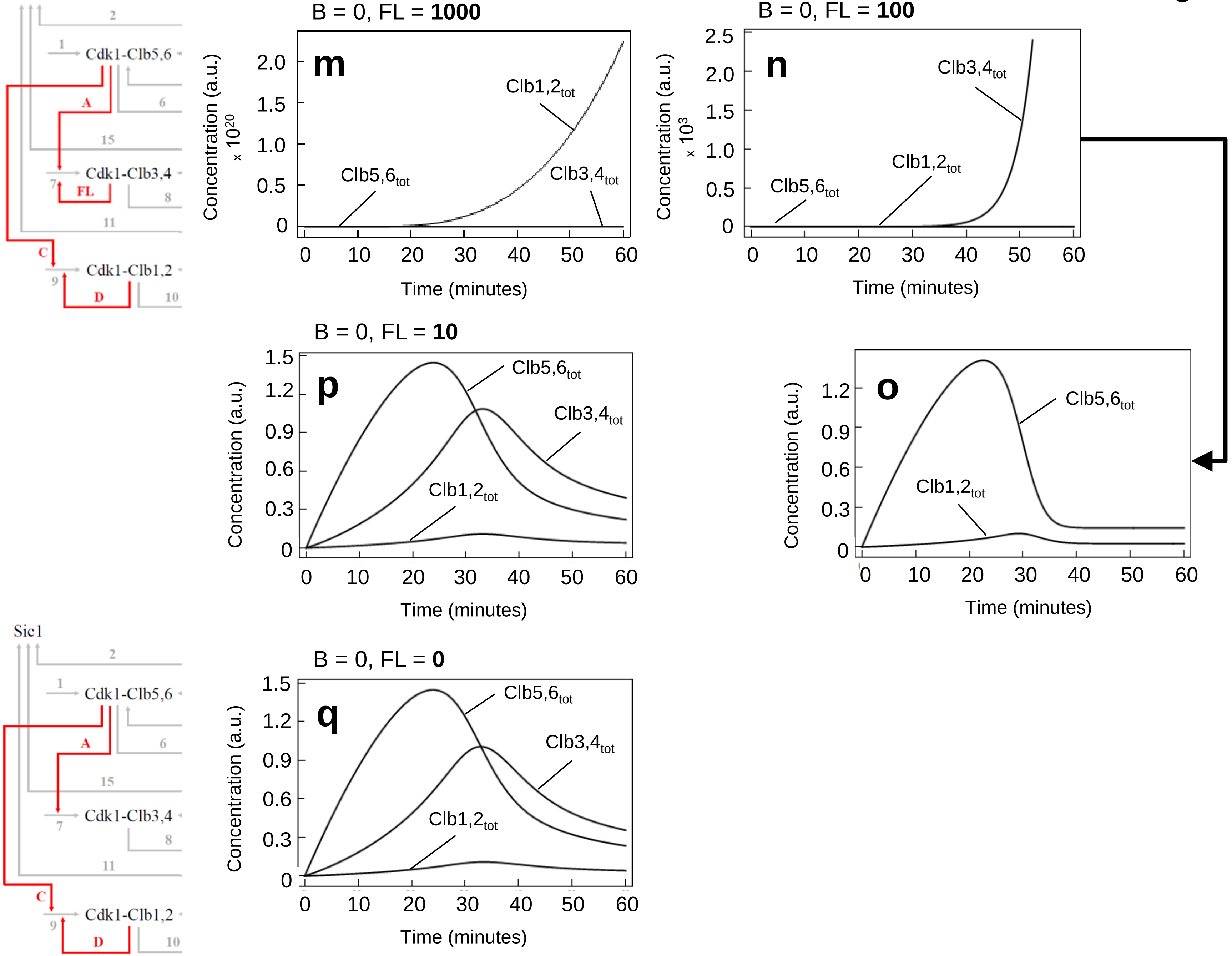

Sic1
Cdk1-Clb5,6
Cdk1-Clb3,4
Cdk1-Clb1,2
A
C
D
FL
1
2
6
7
8
9
10
11
15
B = 0, FL = 1000
m
Concentration (a.u.) x 10^20
Clb1,2tot
Clb5,6tot
Clb3,4tot
Time (minutes)
B = 0, FL = 100
n
Concentration (a.u.) x 10^3
Clb3,4tot
Clb1,2tot
Clb5,6tot
Time (minutes)
B = 0, FL = 10
p
Concentration (a.u.)
Clb5,6tot
Clb3,4tot
Clb1,2tot
Time (minutes)
o
Concentration (a.u.)
Clb5,6tot
Clb1,2tot
Time (minutes)
B = 0, FL = 0
q
Concentration (a.u.)
Clb5,6tot
Clb3,4tot
Clb1,2tot
Time (minutes)

Figure S4

a

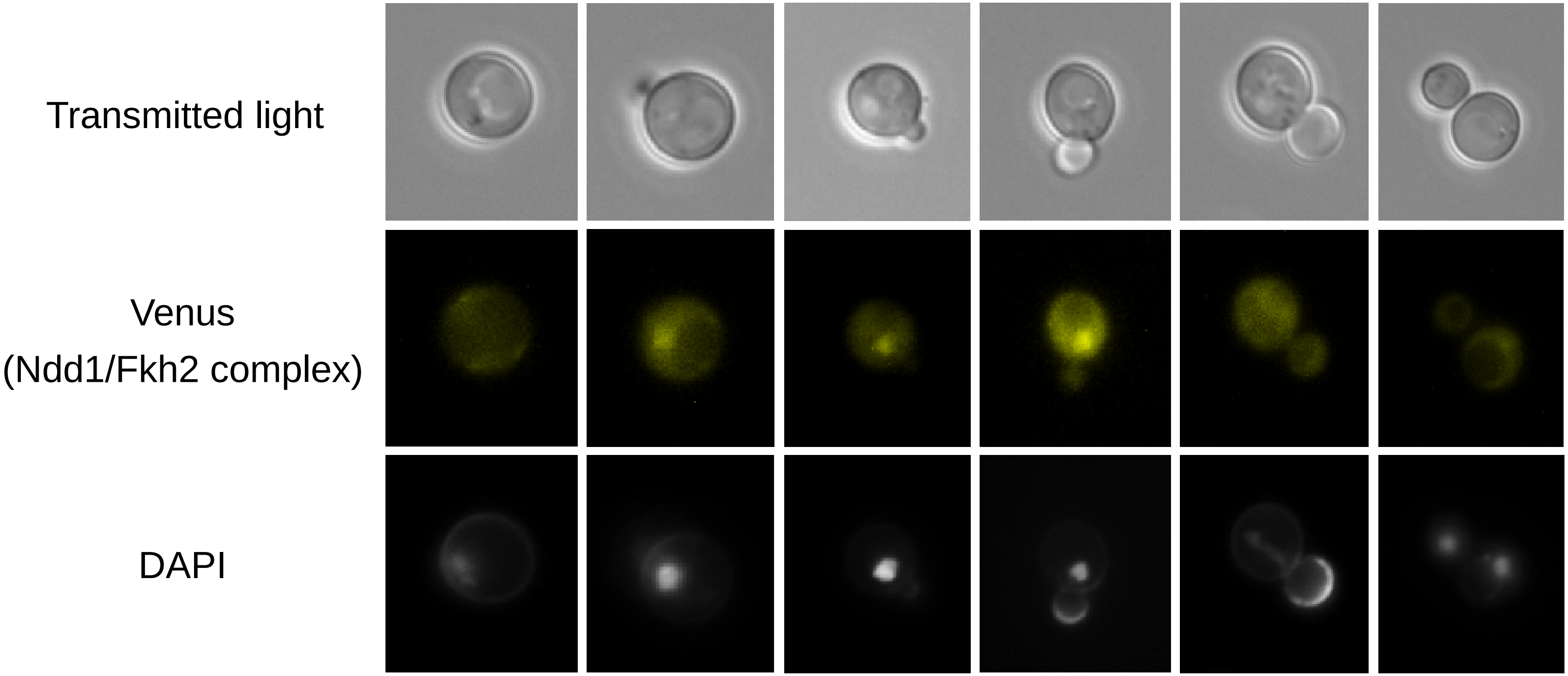

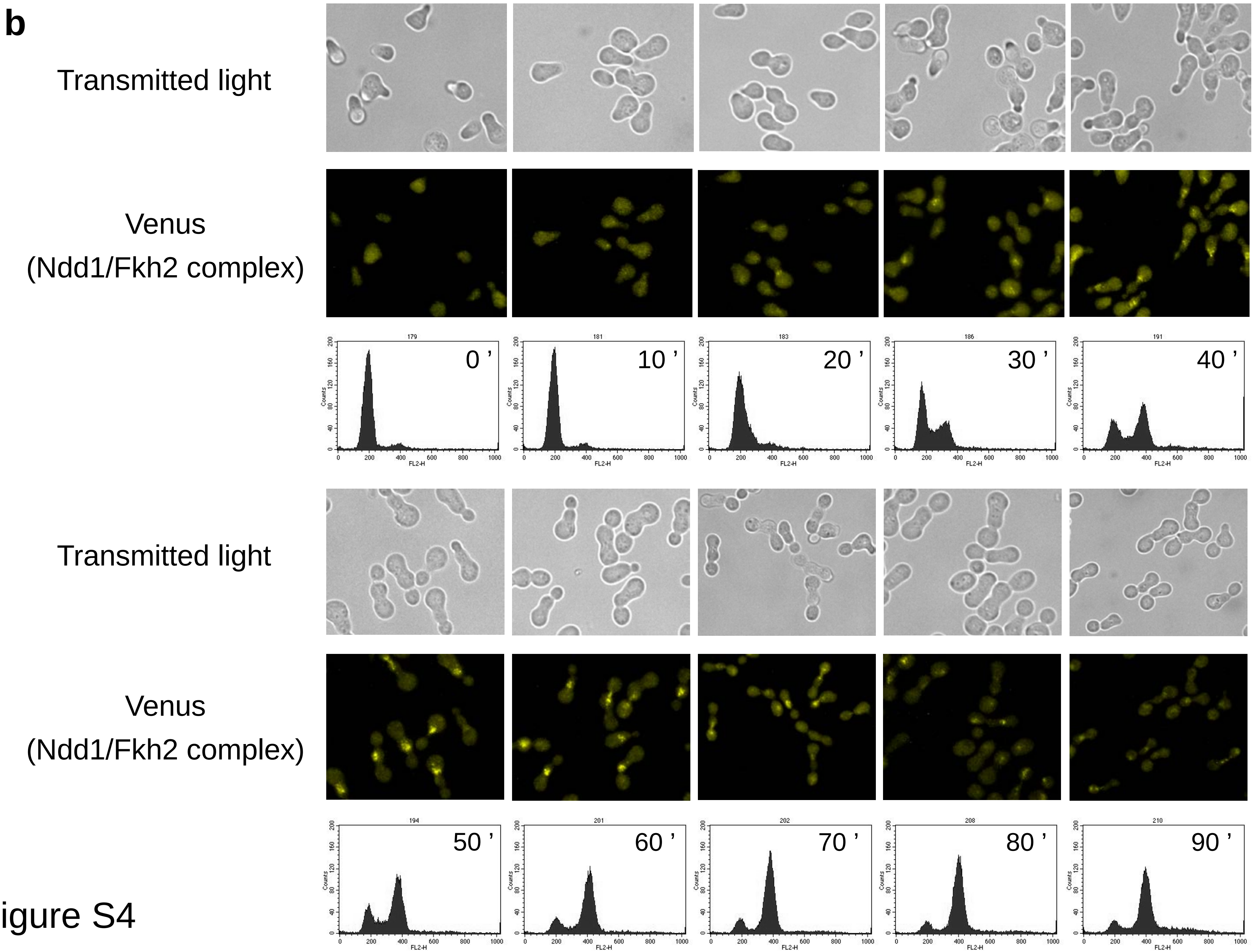
b
Transmitted light
Venus
(Ndd1/Fkh2 complex)
0 '
10 '
20 '
30 '
40 '
Transmitted light
Venus
(Ndd1/Fkh2 complex)
50 '
60 '
70 '
80 '
90 '
Counts
FL2-H

Figure S4

Figure S5

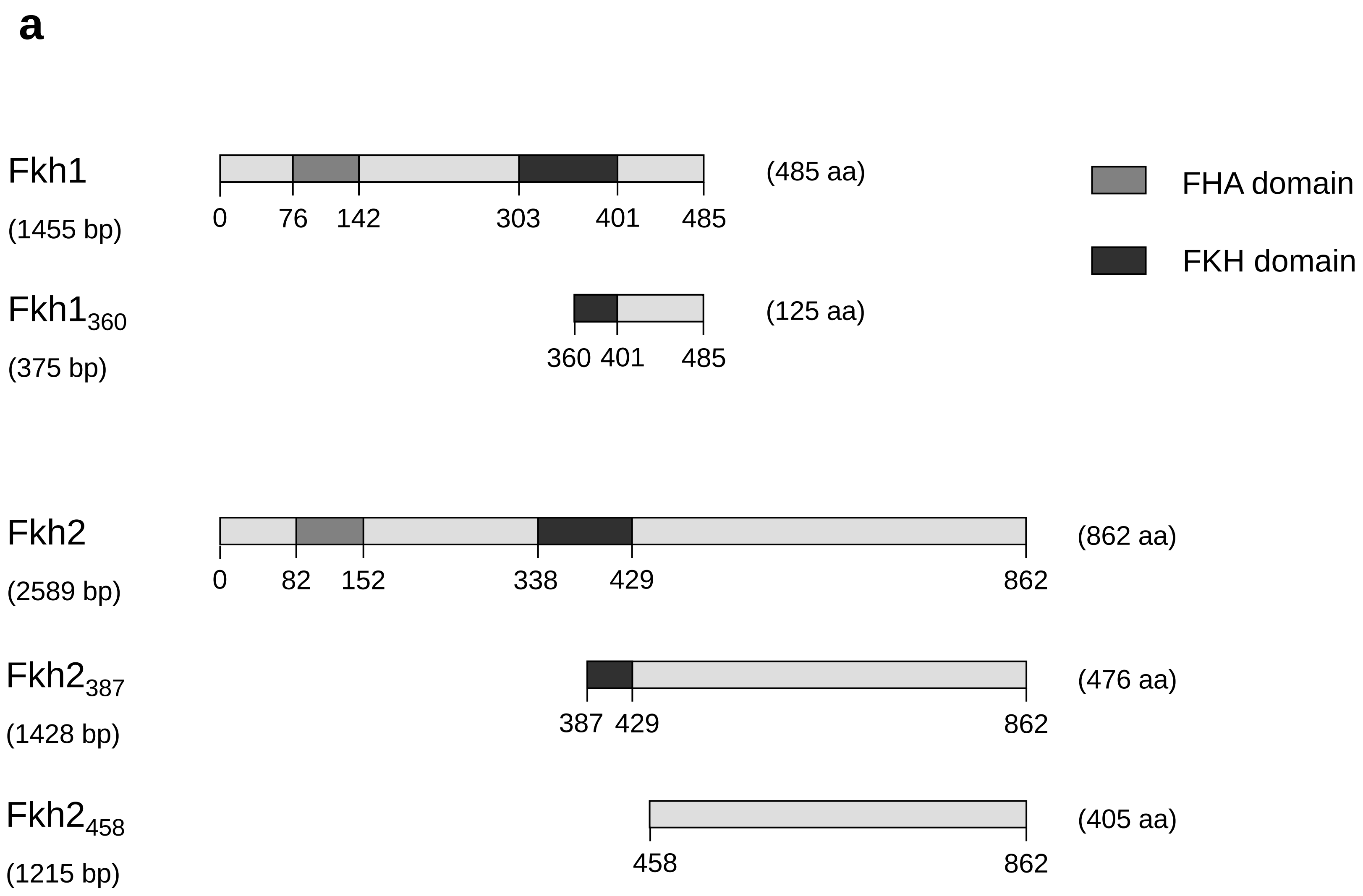
a
Fkh1
(1455 bp)
0
76
142
303
401
485
(485 aa)
FHA domain
FKH domain
Fkh1360
(375 bp)
360
401
485
(125 aa)
Fkh2
(2589 bp)
0
82
152
338
429
862
(862 aa)
Fkh2387
(1428 bp)
387
429
862
(476 aa)
Fkh2458
(1215 bp)
458
862
(405 aa)

b

Figure S5

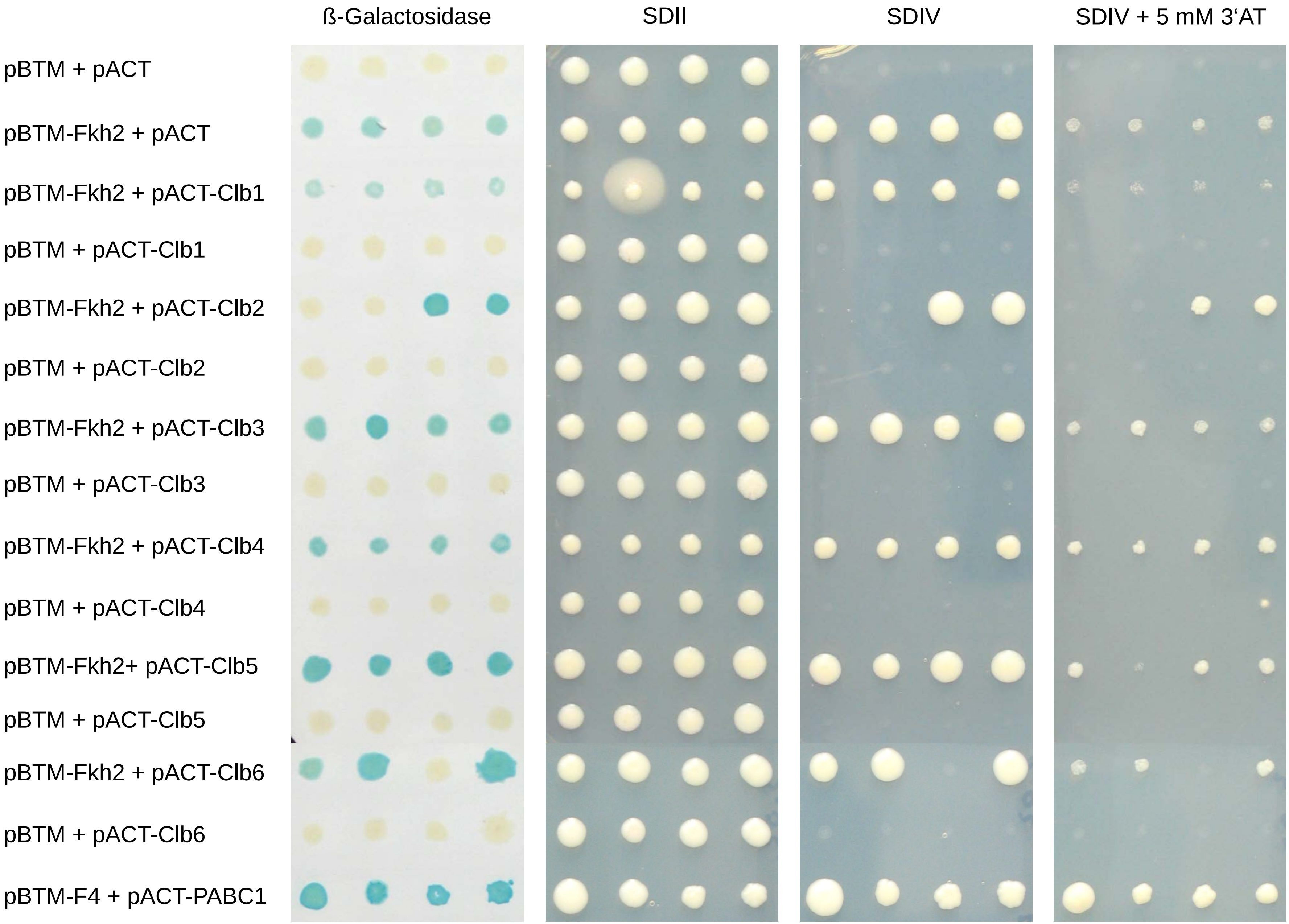
ß-Galactosidase
SDII
SDIV
SDIV + 5 mM 3‘AT
pBTM + pACT
pBTM-Fkh2 + pACT
pBTM-Fkh2 + pACT-Clb1
pBTM + pACT-Clb1
pBTM-Fkh2 + pACT-Clb2
pBTM + pACT-Clb2
pBTM-Fkh2 + pACT-Clb3
pBTM + pACT-Clb3
pBTM-Fkh2 + pACT-Clb4
pBTM + pACT-Clb4
pBTM-Fkh2+ pACT-Clb5
pBTM + pACT-Clb5
pBTM-Fkh2 + pACT-Clb6
pBTM + pACT-Clb6
pBTM-F4 + pACT-PABC1

Figure S5

c

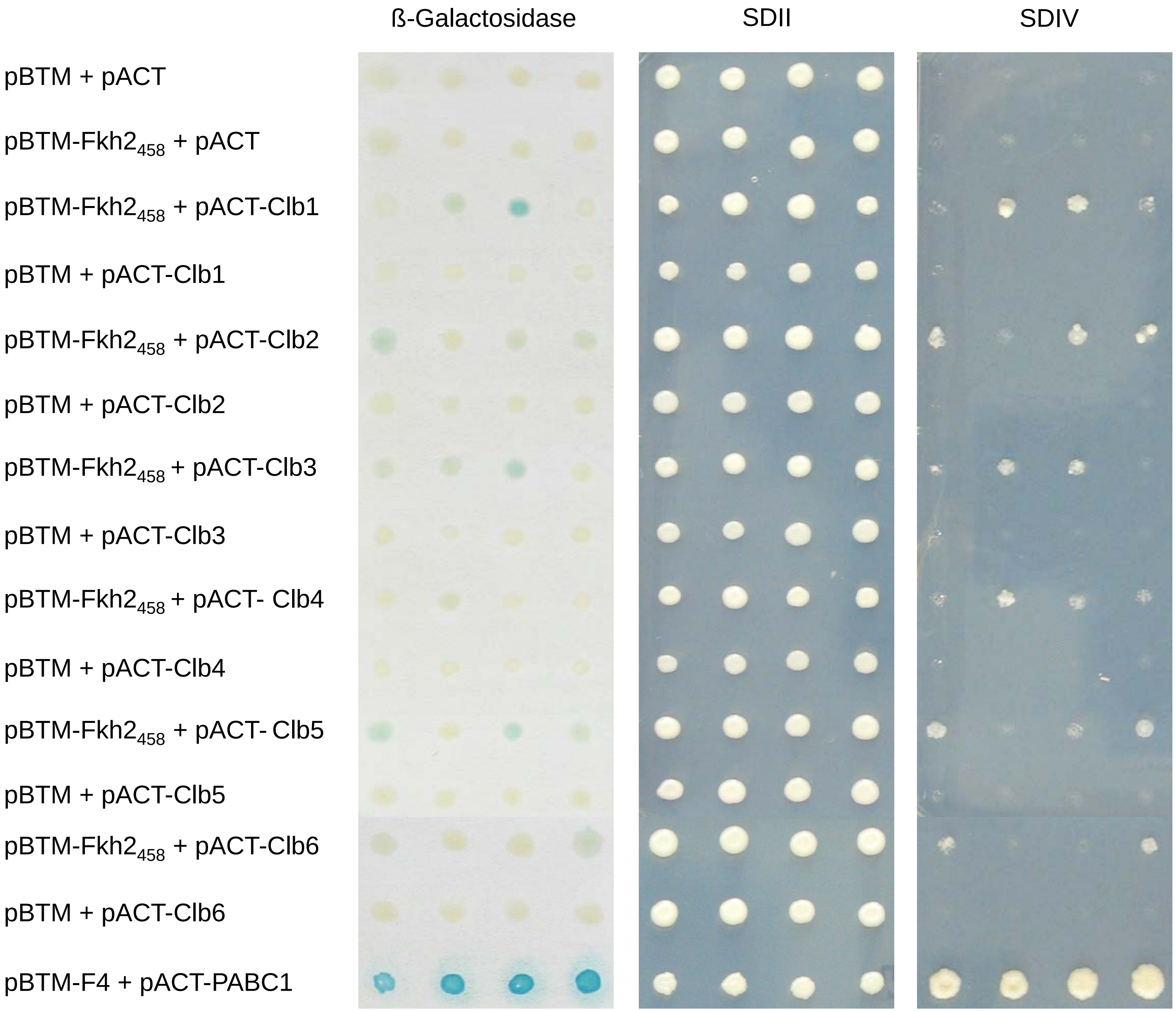
ß-Galactosidase
SDII
SDIV
pBTM + pACT
pBTM-Fkh2458 + pACT
pBTM-Fkh2458 + pACT-Clb1
pBTM + pACT-Clb1
pBTM-Fkh2458 + pACT-Clb2
pBTM + pACT-Clb2
pBTM-Fkh2458 + pACT-Clb3
pBTM + pACT-Clb3
pBTM-Fkh2458 + pACT- Clb4
pBTM + pACT-Clb4
pBTM-Fkh2458 + pACT- Clb5
pBTM + pACT-Clb5
pBTM-Fkh2458 + pACT-Clb6
pBTM + pACT-Clb6
pBTM-F4 + pACT-PABC1

Figure S5

d

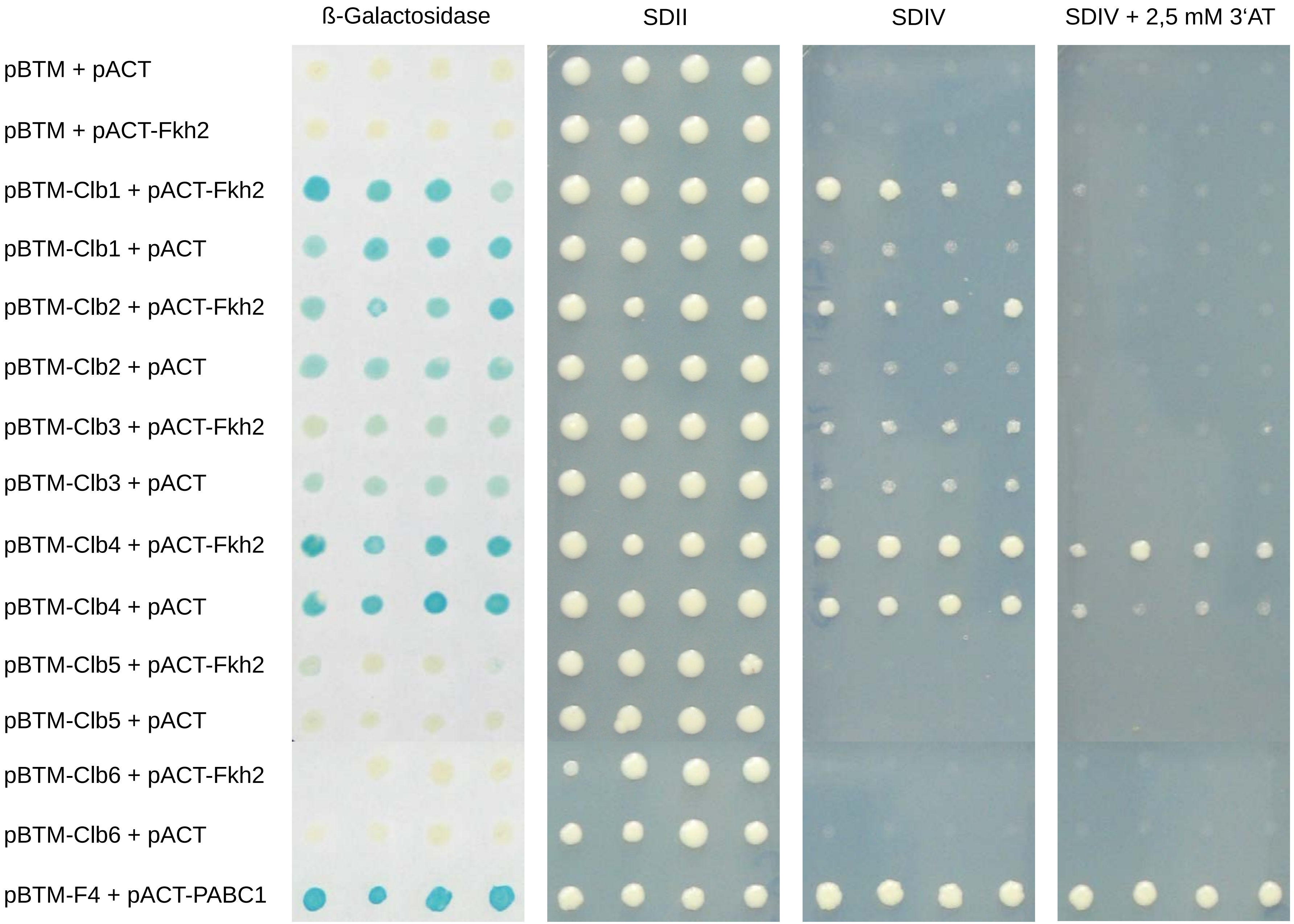

ß-Galactosidase
SDII
SDIV
SDIV + 2,5 mM 3‘AT
pBTM + pACT
pBTM + pACT-Fkh2
pBTM-Clb1 + pACT-Fkh2
pBTM-Clb1 + pACT
pBTM-Clb2 + pACT-Fkh2
pBTM-Clb2 + pACT
pBTM-Clb3 + pACT-Fkh2
pBTM-Clb3 + pACT
pBTM-Clb4 + pACT-Fkh2
pBTM-Clb4 + pACT
pBTM-Clb5 + pACT-Fkh2
pBTM-Clb5 + pACT
pBTM-Clb6 + pACT-Fkh2
pBTM-Clb6 + pACT
pBTM-F4 + pACT-PABC1

Figure S5

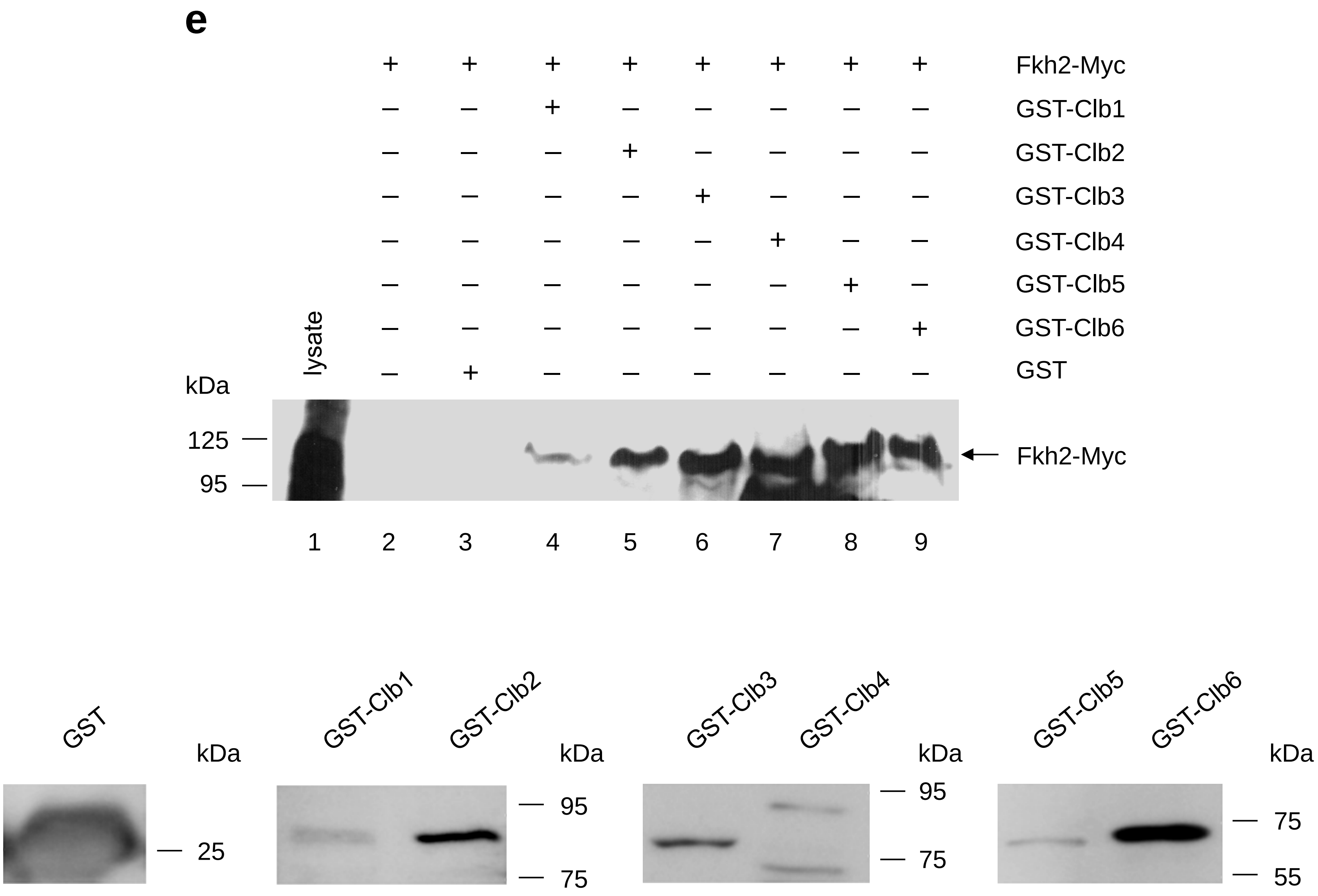

e
Fkh2-Myc
GST-Clb1
GST-Clb2
GST-Clb3
GST-Clb4
GST-Clb5
GST-Clb6
GST
lysate
kDa
125
95
Fkh2-Myc
1 2 3 4 5 6 7 8 9
GST
kDa
25
GST-Clb1
GST-Clb2
kDa
95
75
GST-Clb3
GST-Clb4
kDa
95
75
GST-Clb5
GST-Clb6
kDa
75
55

f

Figure S5

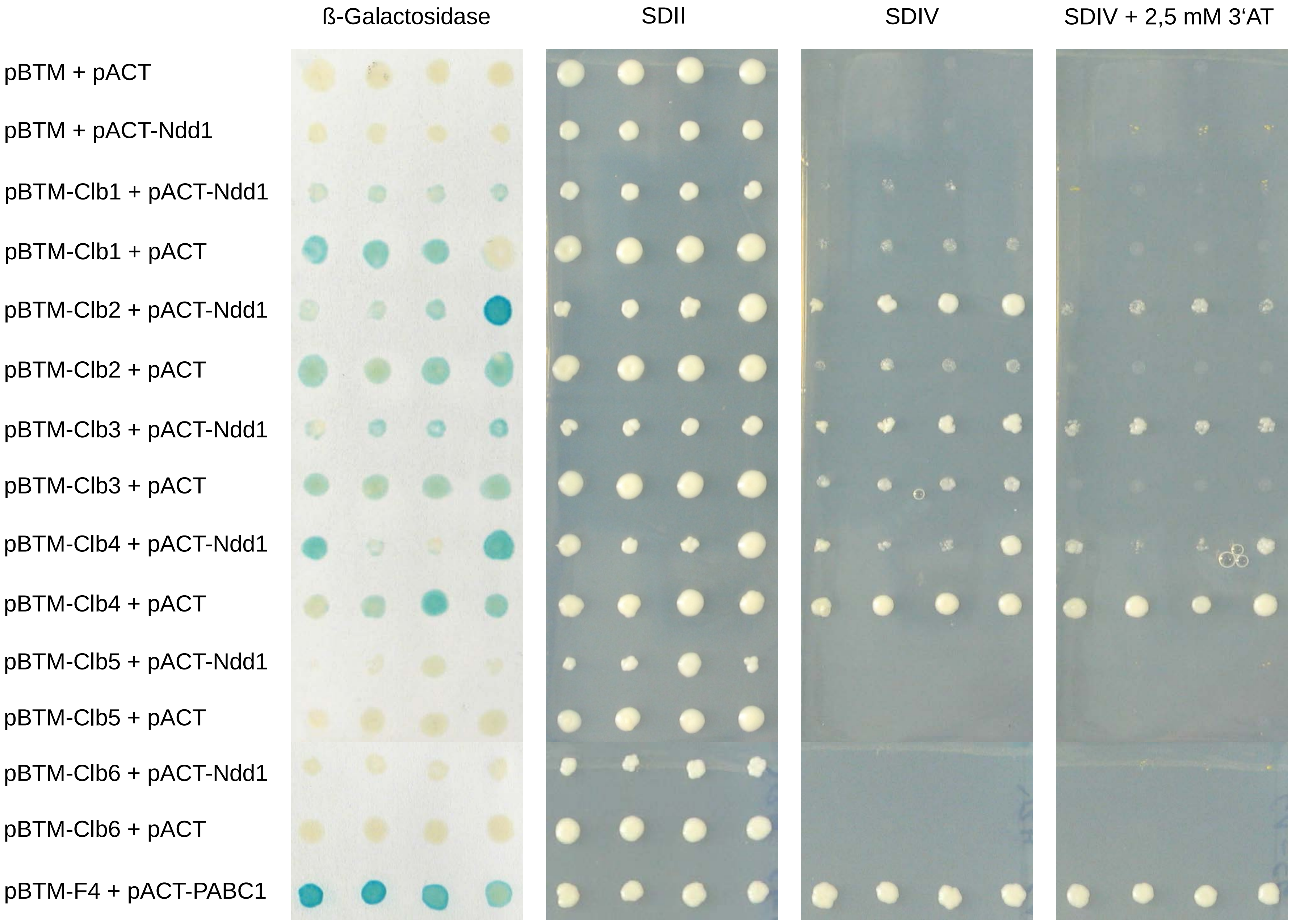
ß-Galactosidase
SDII
SDIV
SDIV + 2,5 mM 3‘AT
pBTM + pACT
pBTM + pACT-Ndd1
pBTM-Clb1 + pACT-Ndd1
pBTM-Clb1 + pACT
pBTM-Clb2 + pACT-Ndd1
pBTM-Clb2 + pACT
pBTM-Clb3 + pACT-Ndd1
pBTM-Clb3 + pACT
pBTM-Clb4 + pACT-Ndd1
pBTM-Clb4 + pACT
pBTM-Clb5 + pACT-Ndd1
pBTM-Clb5 + pACT
pBTM-Clb6 + pACT-Ndd1
pBTM-Clb6 + pACT
pBTM-F4 + pACT-PABC1

Figure S5

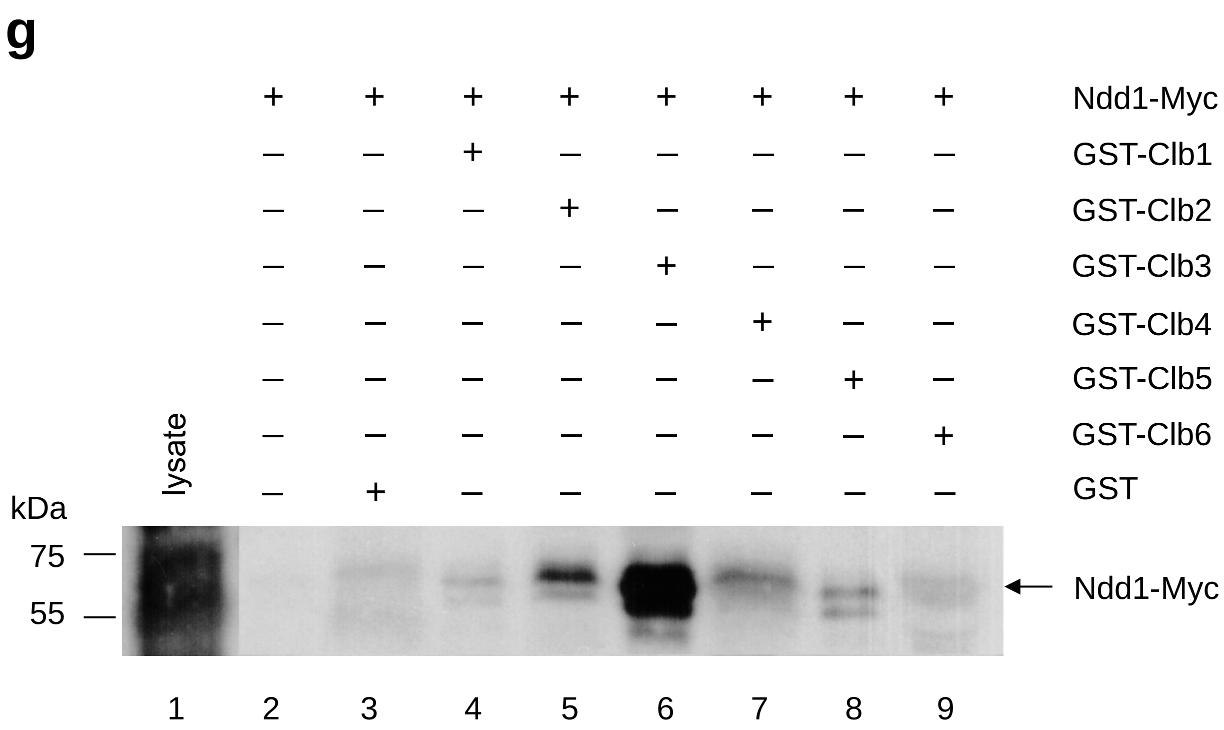
g
+ + + + + + + + Ndd1-Myc
– – + – – – – – GST-Clb1
– – – + – – – – GST-Clb2
– – – – + – – – GST-Clb3
– – – – – + – – GST-Clb4
– – – – – – + – GST-Clb5
– – – – – – – + GST-Clb6
– + – – – – – – GST
lysate
kDa
75
55
Ndd1-Myc
1 2 3 4 5 6 7 8 9

Figure S5

h

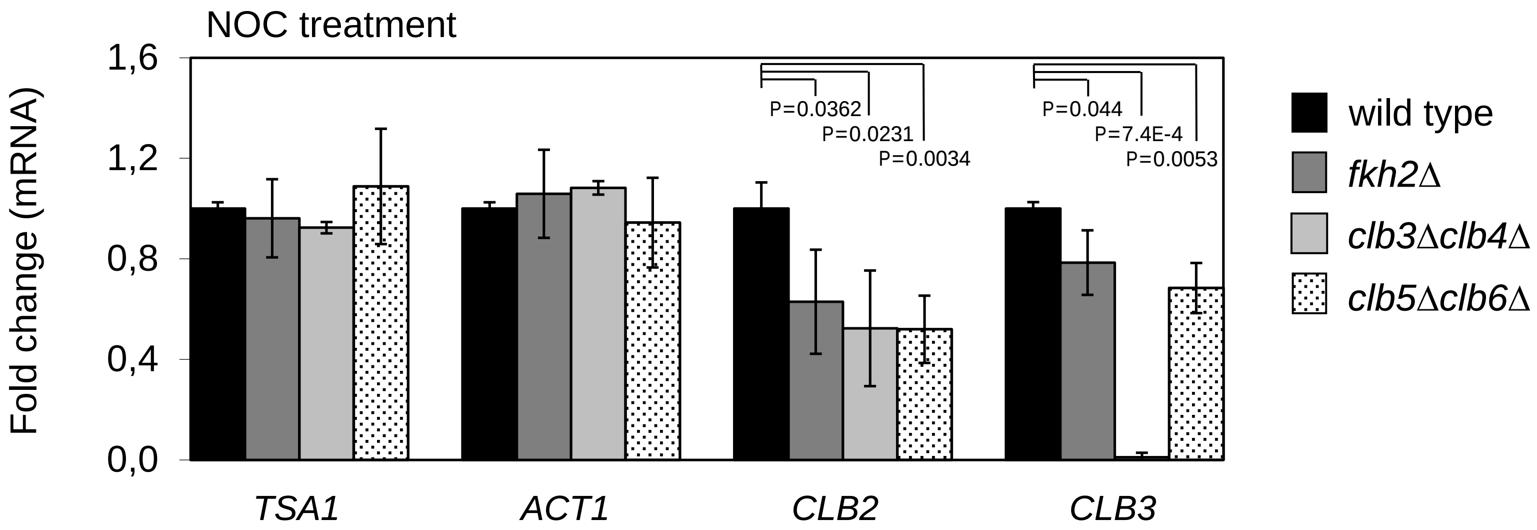

NOC treatment
Fold change (mRNA)
1,6
1,2
0,8
0,4
0,0
P=0.0362
P=0.0231
P=0.0034
P=0.044
P=7.4E-4
P=0.0053
TSA1
ACT1
CLB2
CLB3
wild type
fkh2Δ
clb3Δclb4Δ
clb5Δclb6Δ

Figure S6

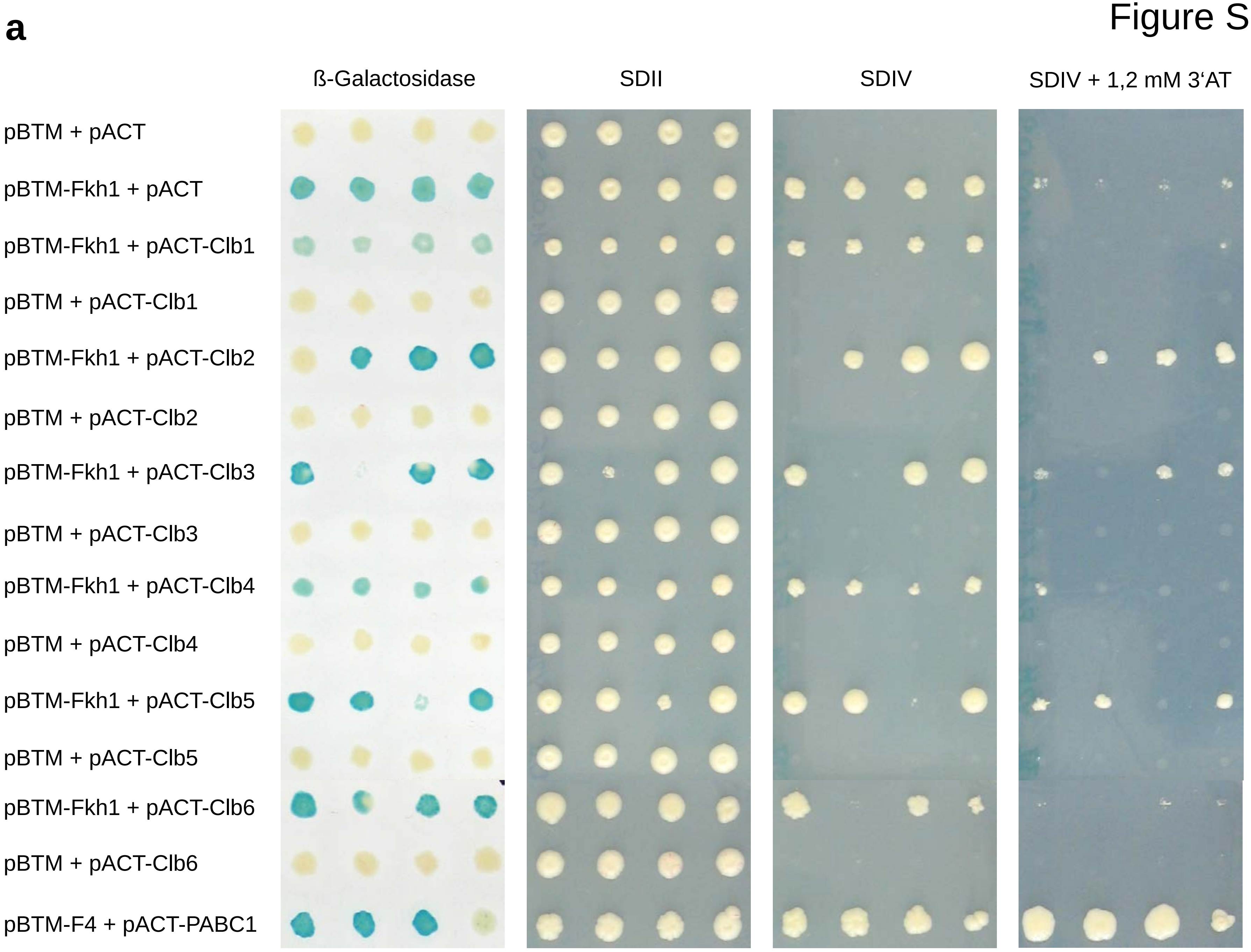
a
ß-Galactosidase
SDII
SDIV
SDIV + 1,2 mM 3‘AT
pBTM + pACT
pBTM-Fkh1 + pACT
pBTM-Fkh1 + pACT-Clb1
pBTM + pACT-Clb1
pBTM-Fkh1 + pACT-Clb2
pBTM + pACT-Clb2
pBTM-Fkh1 + pACT-Clb3
pBTM + pACT-Clb3
pBTM-Fkh1 + pACT-Clb4
pBTM + pACT-Clb4
pBTM-Fkh1 + pACT-Clb5
pBTM + pACT-Clb5
pBTM-Fkh1 + pACT-Clb6
pBTM + pACT-Clb6
pBTM-F4 + pACT-PABC1

Figure S6

b

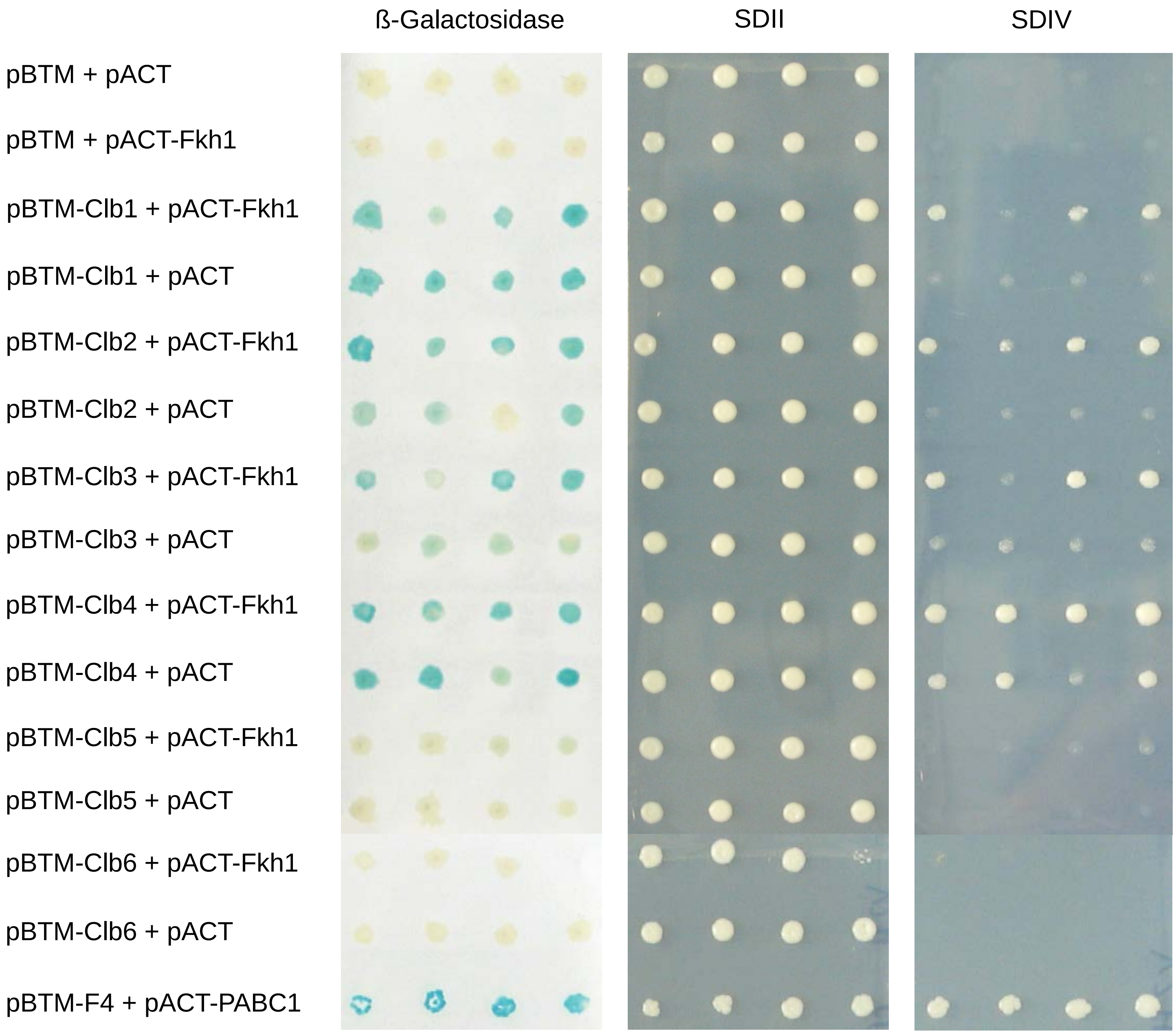
ß-Galactosidase
SDII
SDIV
pBTM + pACT
pBTM + pACT-Fkh1
pBTM-Clb1 + pACT-Fkh1
pBTM-Clb1 + pACT
pBTM-Clb2 + pACT-Fkh1
pBTM-Clb2 + pACT
pBTM-Clb3 + pACT-Fkh1
pBTM-Clb3 + pACT
pBTM-Clb4 + pACT-Fkh1
pBTM-Clb4 + pACT
pBTM-Clb5 + pACT-Fkh1
pBTM-Clb5 + pACT
pBTM-Clb6 + pACT-Fkh1
pBTM-Clb6 + pACT
pBTM-F4 + pACT-PABC1

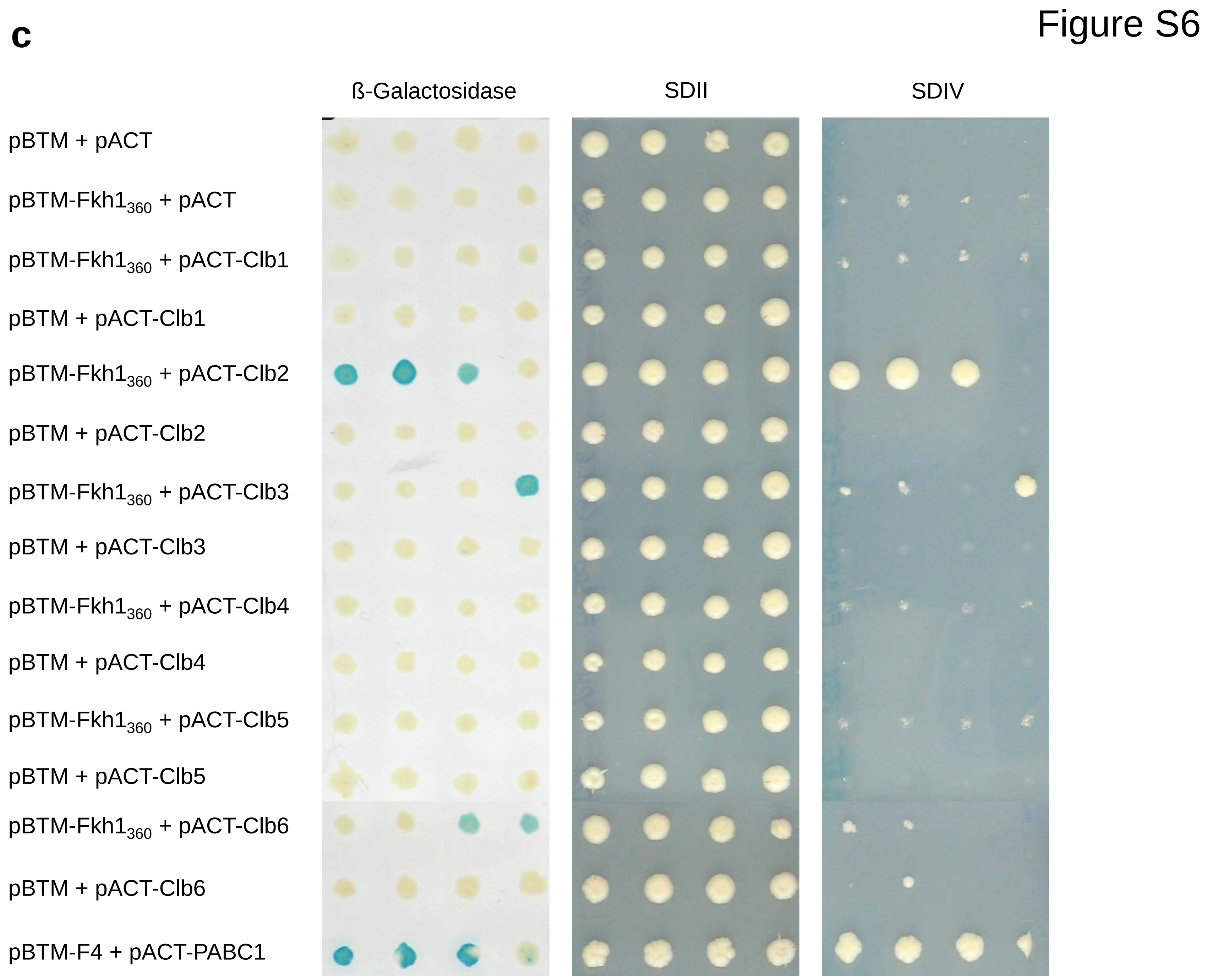

c
Figure S6
ß-Galactosidase
SDII
SDIV
pBTM + pACT
pBTM-Fkh1360 + pACT
pBTM-Fkh1360 + pACT-Clb1
pBTM + pACT-Clb1
pBTM-Fkh1360 + pACT-Clb2
pBTM + pACT-Clb2
pBTM-Fkh1360 + pACT-Clb3
pBTM + pACT-Clb3
pBTM-Fkh1360 + pACT-Clb4
pBTM + pACT-Clb4
pBTM-Fkh1360 + pACT-Clb5
pBTM + pACT-Clb5
pBTM-Fkh1360 + pACT-Clb6
pBTM + pACT-Clb6
pBTM-F4 + pACT-PABC1

Figure S6

d

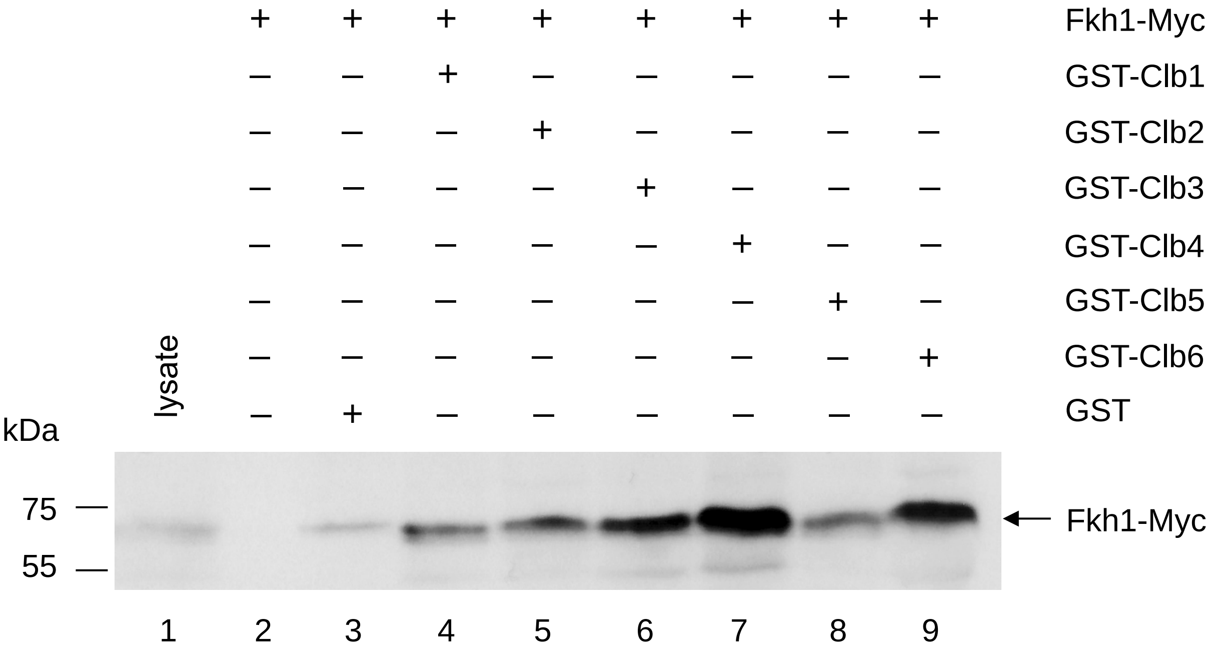
Fkh1-Myc
GST-Clb1
GST-Clb2
GST-Clb3
GST-Clb4
GST-Clb5
GST-Clb6
GST
lysate
kDa
75
55
Fkh1-Myc
1 2 3 4 5 6 7 8 9

Figure S7

a

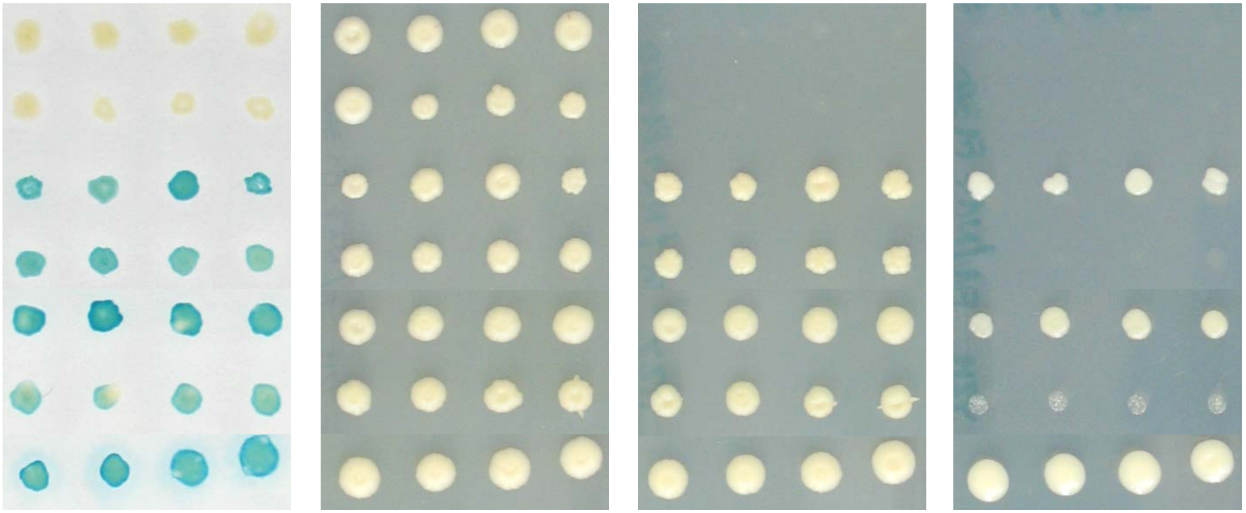
ß-Galactosidase
SDII
SDIV
SDIV + 10 mM 3‘AT
pBTM + pACT
pBTM + pACT-Ndd1
pBTM-Fkh1 + pACT-Ndd1
pBTM-Fkh1 + pACT
pBTM-Fkh2 + pACT-Ndd1
pBTM-Fkh2 + pACT
pBTM-F4 + pACT-PABC1

b

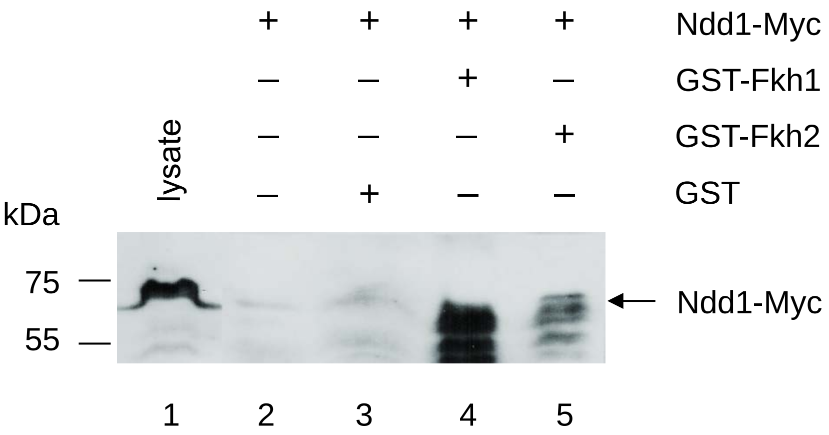

+ + + + Ndd1-Myc
– – + – GST-Fkh1
– – – + GST-Fkh2
lysate
– + – – GST
kDa
75
55
Ndd1-Myc
1 2 3 4 5

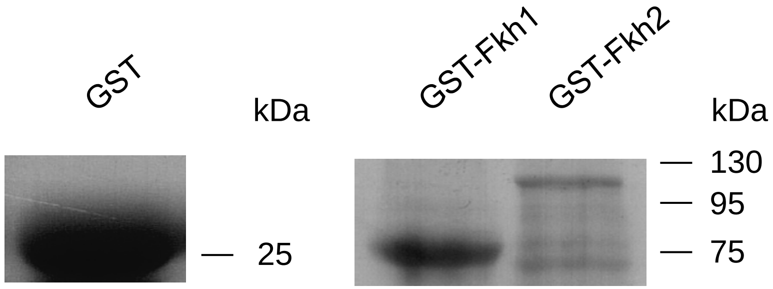

GST
kDa
25
GST-Fkh1
GST-Fkh2
kDa
130
95
75

c

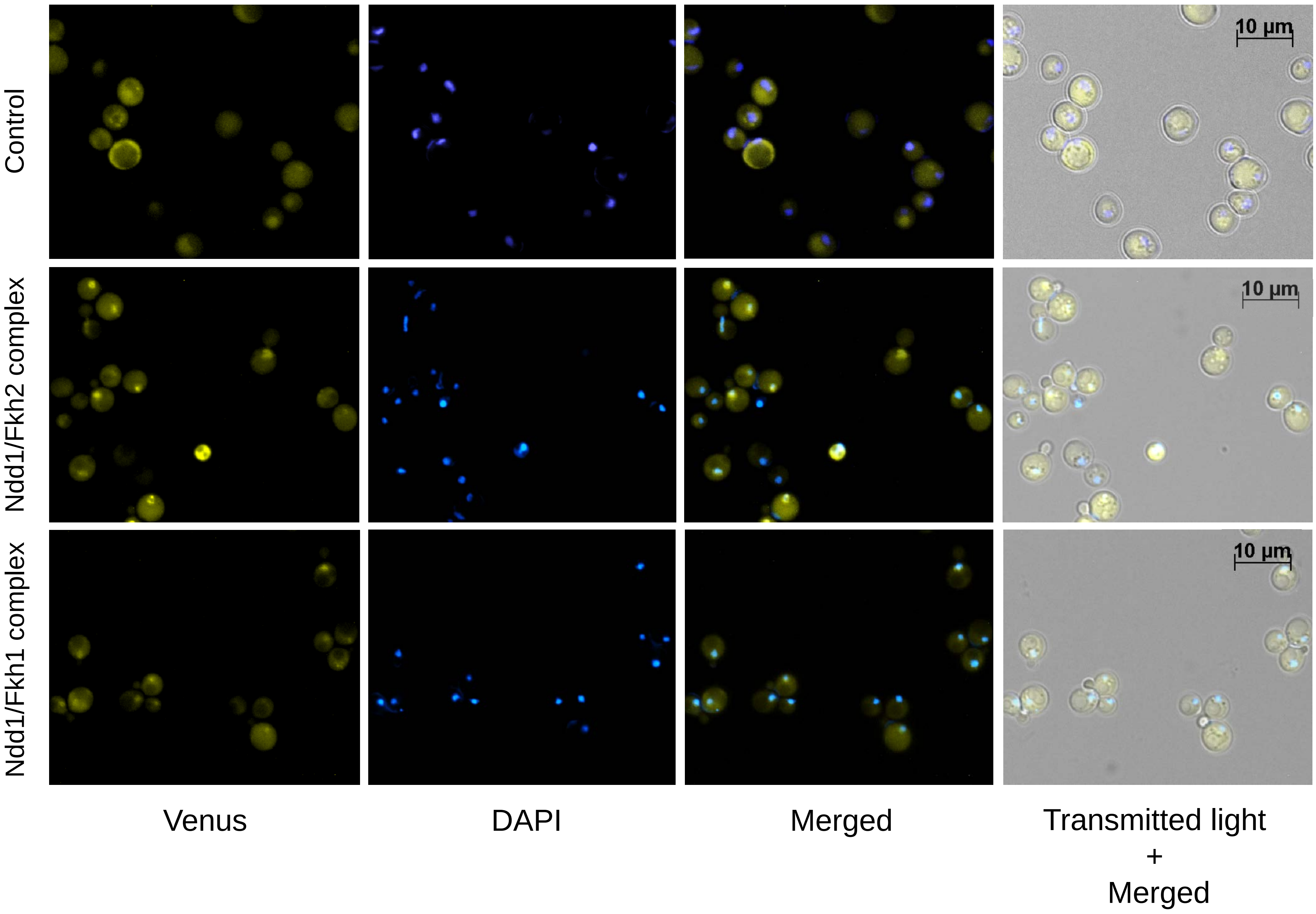
Control
Ndd1/Fkh2 complex
Ndd1/Fkh1 complex
10 µm
10 µm
10 µm
Venus
DAPI
Merged
Transmitted light
+
Merged

Figure S7

d

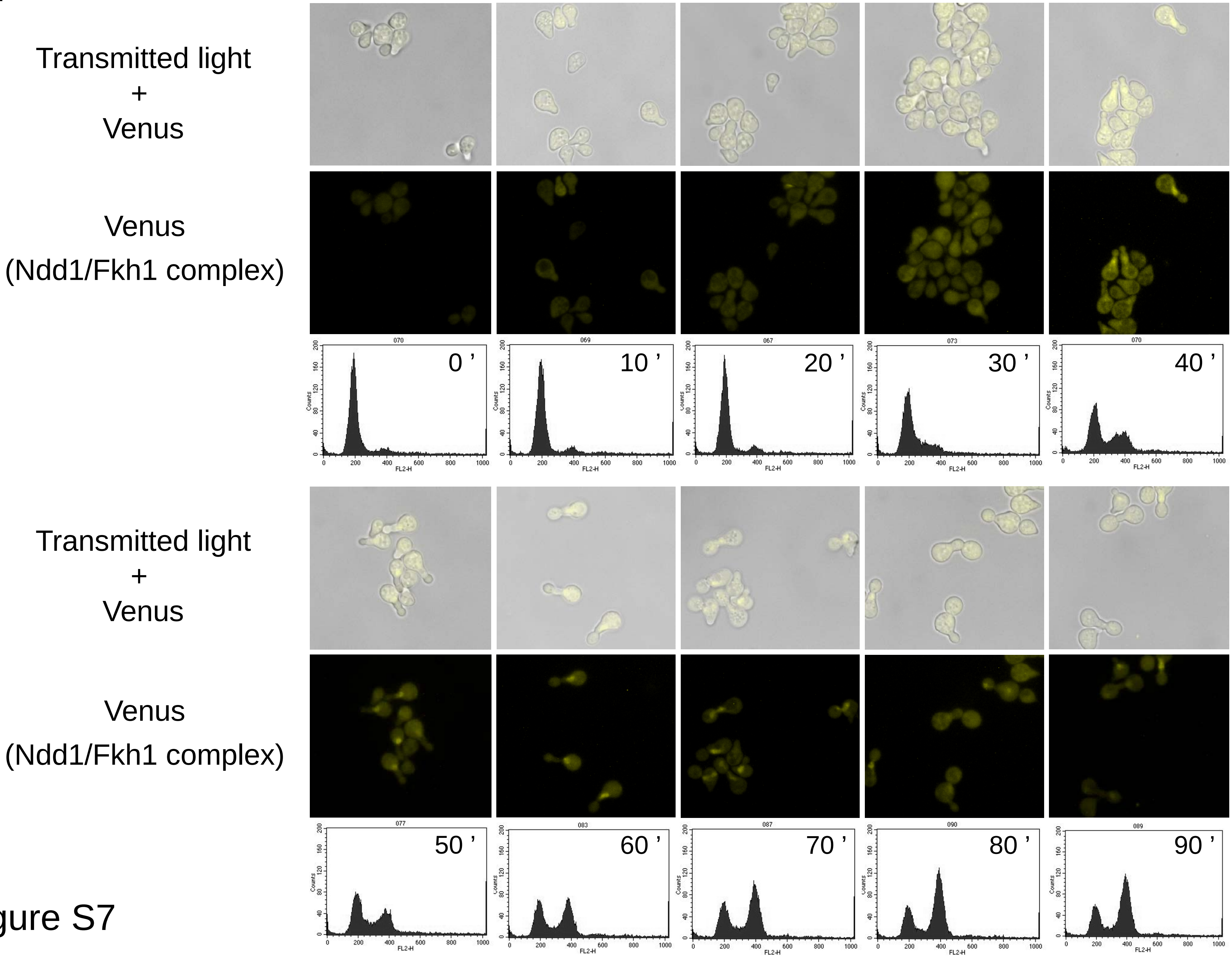

Transmitted light
+
Venus
Venus
(Ndd1/Fkh1 complex)
0 '
10 '
20 '
30 '
40 '
Transmitted light
+
Venus
Venus
(Ndd1/Fkh1 complex)
50 '
60 '
70 '
80 '
90 '
Counts
FL2-H

Figure S7

Figure S8

a

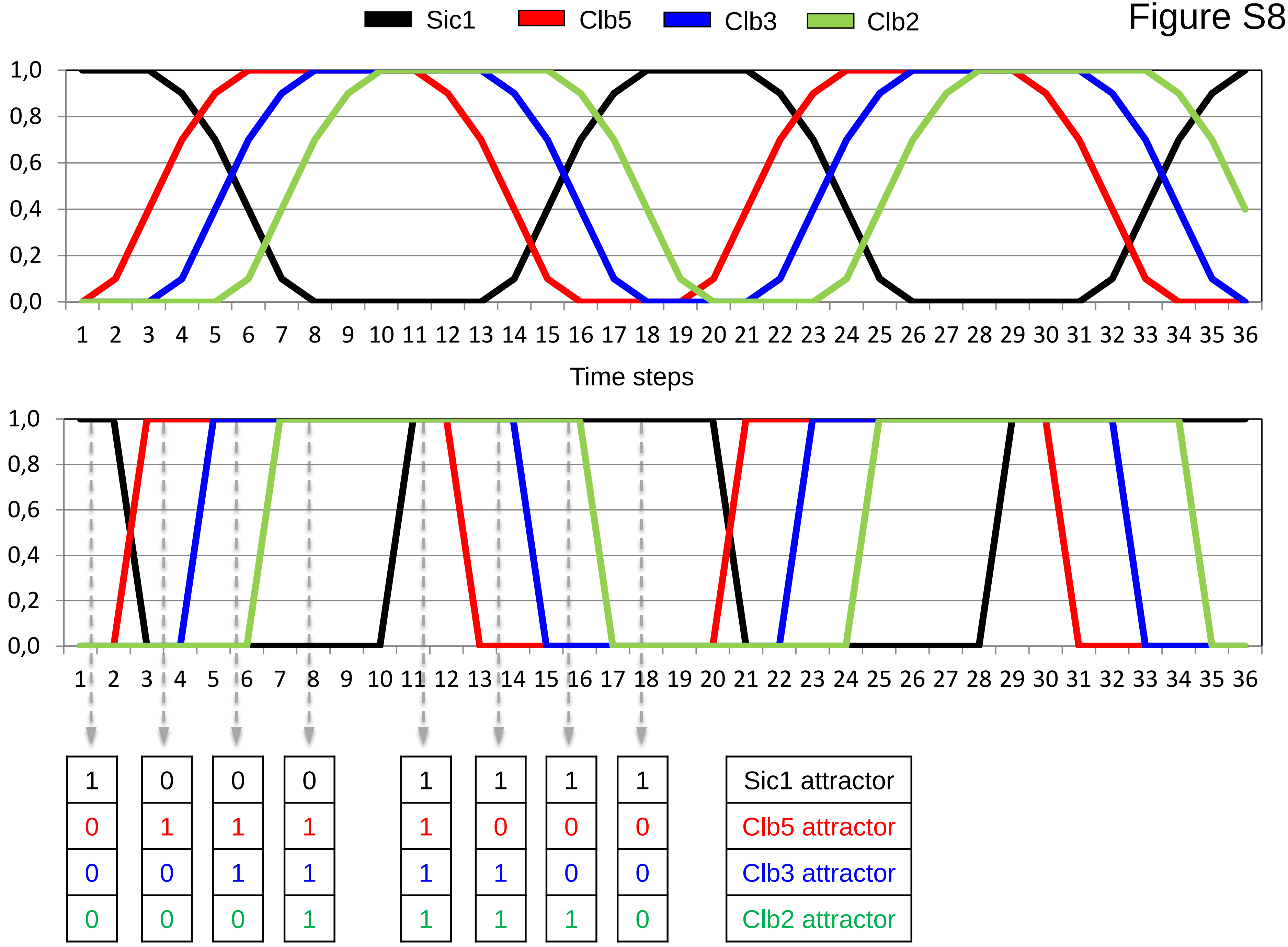
Sic1
Clb5
Clb3
Clb2
1,0
0,8
0,6
0,4
0,2
0,0
1 2 3 4 5 6 7 8 9 10 11 12 13 14 15 16 17 18 19 20 21 22 23 24 25 26 27 28 29 30 31 32 33 34 35 36
Time steps
1,0
0,8
0,6
0,4
0,2
0,0
1 2 3 4 5 6 7 8 9 10 11 12 13 14 15 16 17 18 19 20 21 22 23 24 25 26 27 28 29 30 31 32 33 34 35 36
1 0 0 0 1 1 1 1
0 1 1 1 1 0 0 0
0 0 1 1 1 1 0 0
0 0 0 1 1 1 1 0
Sic1 attractor
Clb5 attractor
Clb3 attractor
Clb2 attractor

Figure S8

b

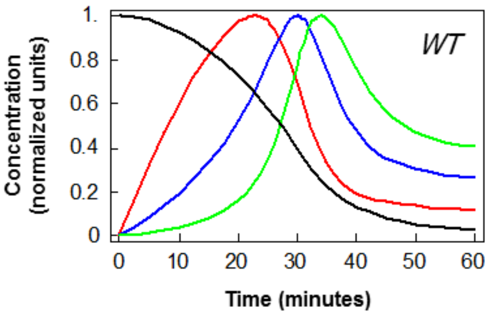

attractor A

Sic1	1	1	0	0	0	1	1
Clb5	0	1	1	1	0	0	0
Clb3	0	0	1	1	1	0	0
Clb2	0	0	0	1	1	1	0

attractor B

Sic1	1	0	0	0	1	1	1	1
Clb5	0	1	1	1	1	0	0	0
Clb3	0	0	1	1	1	1	0	0
Clb2	0	0	0	1	1	1	1	0

attractor C

Sic1	1	1	1	0	0	1	1	1
Clb5	0	1	1	1	0	0	0	0
Clb3	0	0	1	1	1	1	0	0
Clb2	0	0	0	1	1	1	1	0

Figure S8

c

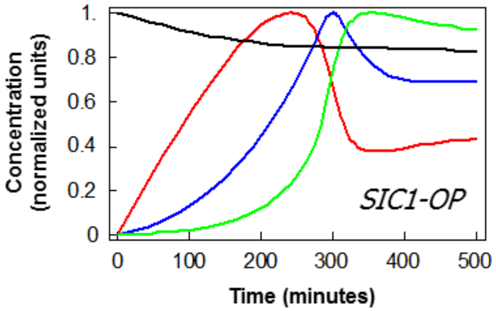

attractor A

Sic1	1	1	1	1	1	1	1
Clb5	0	1	1	1	0	0	0
Clb3	0	0	1	1	1	0	0
Clb2	0	0	0	1	1	1	0

attractor B

Sic1	1	1	1	1	1	1	1	1
Clb5	0	1	1	1	1	0	0	0
Clb3	0	0	1	1	1	1	0	0
Clb2	0	0	0	1	1	1	1	0

attractor C

Sic1	1	1	1	1	1	1	1	1
Clb5	0	1	1	1	0	0	0	0
Clb3	0	0	1	1	1	1	0	0
Clb2	0	0	0	1	1	1	1	0

Figure S8

d

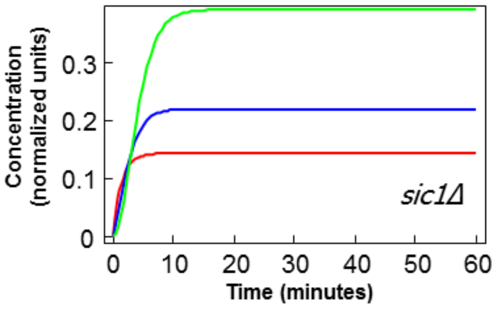

attractor A

Sic1	0
Clb5	1
Clb3	1
Clb2	1

Figure S9

a

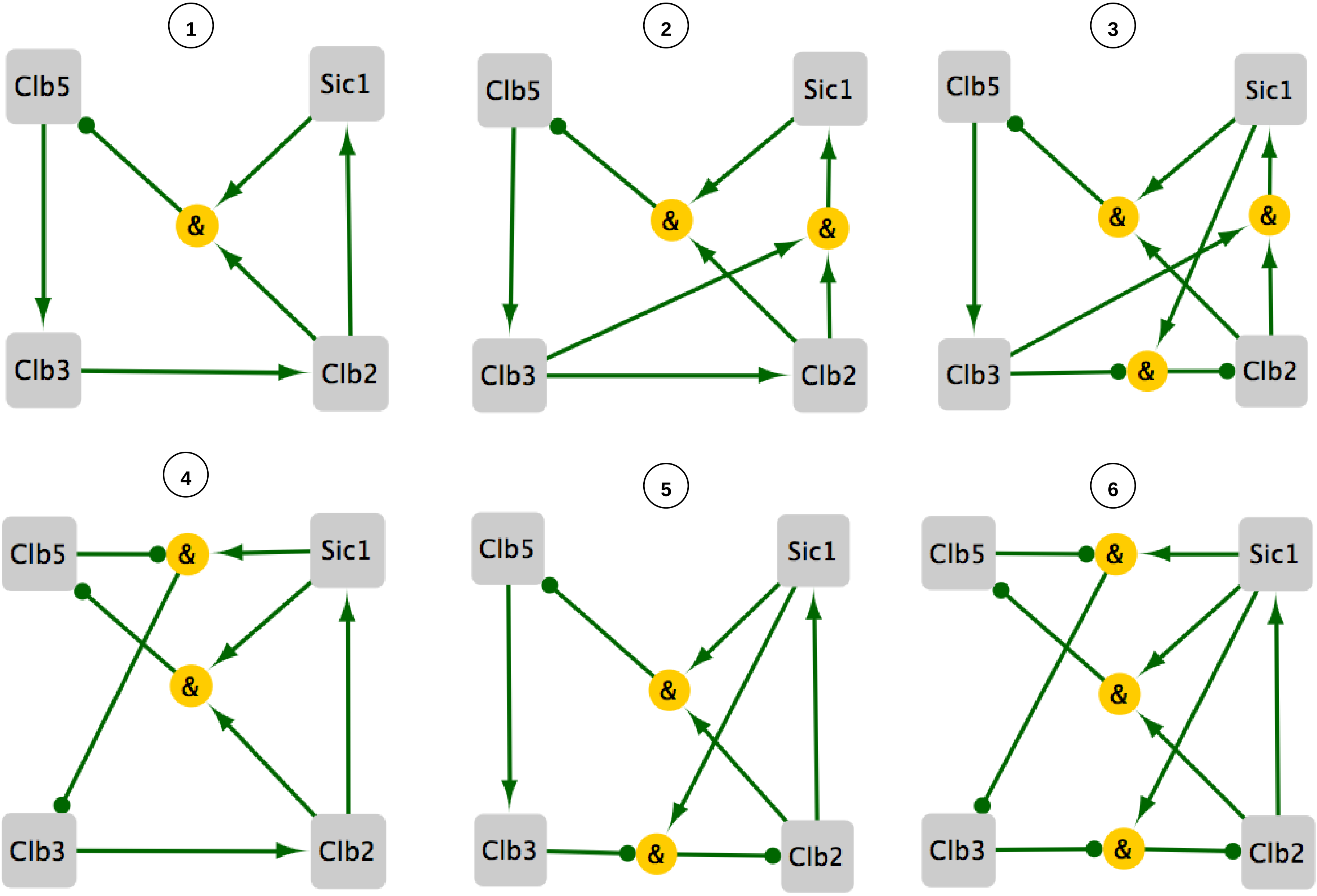
1
Clb5
Sic1
&
Clb3
Clb2
2
Clb5
Sic1
&
&
Clb3
Clb2
3
Clb5
Sic1
&
&
Clb3
&
Clb2
4
Clb5
&
Sic1
&
Clb3
Clb2
5
Clb5
Sic1
&
Clb3
&
Clb2
6
Clb5
&
Sic1
&
Clb3
&
Clb2

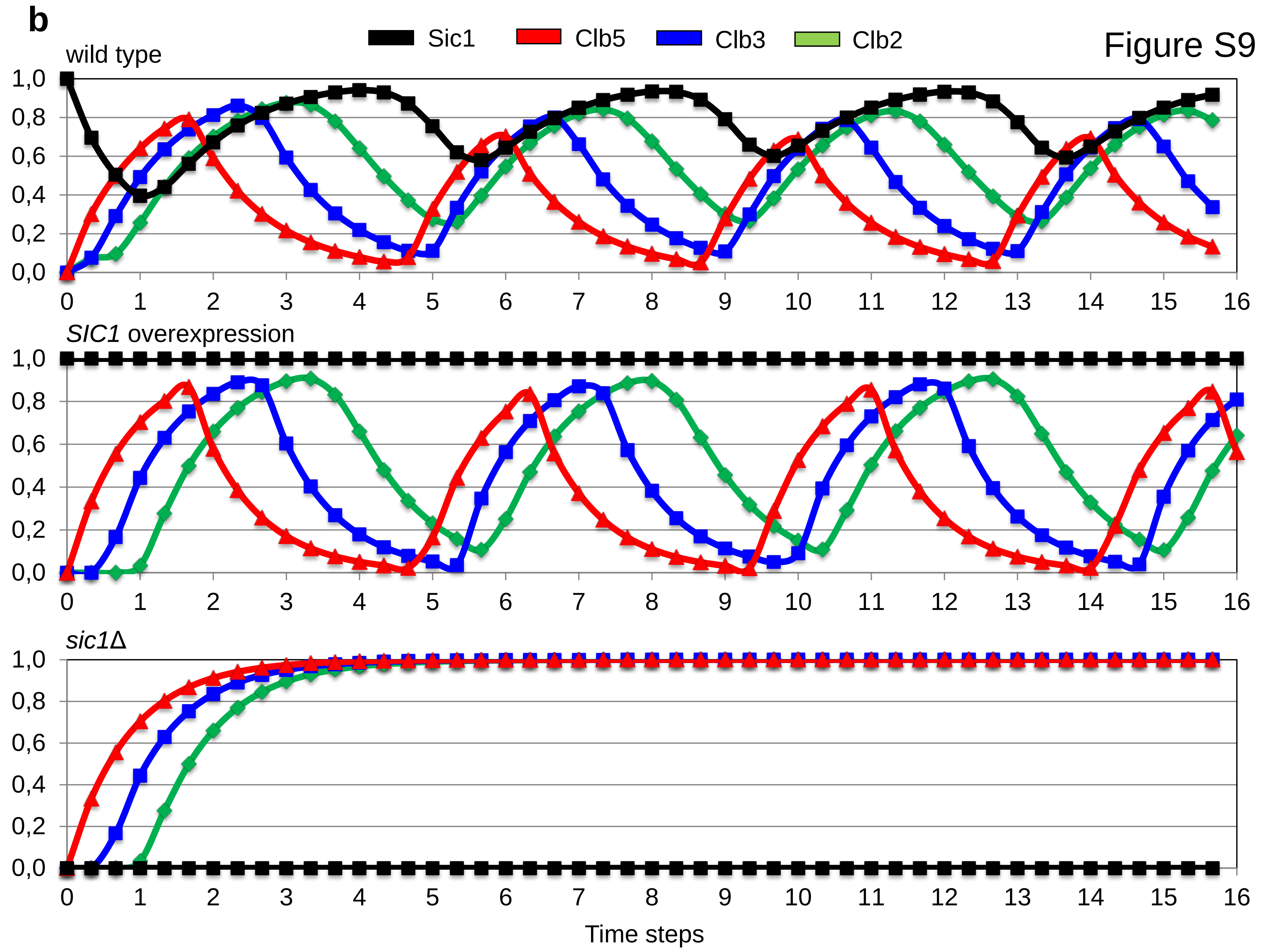
b
Figure S9
Sic1
Clb5
Clb3
Clb2
wild type
SIC1 overexpression
sic1Δ
1,0
0,8
0,6
0,4
0,2
0,0
0 1 2 3 4 5 6 7 8 9 10 11 12 13 14 15 16
Time steps

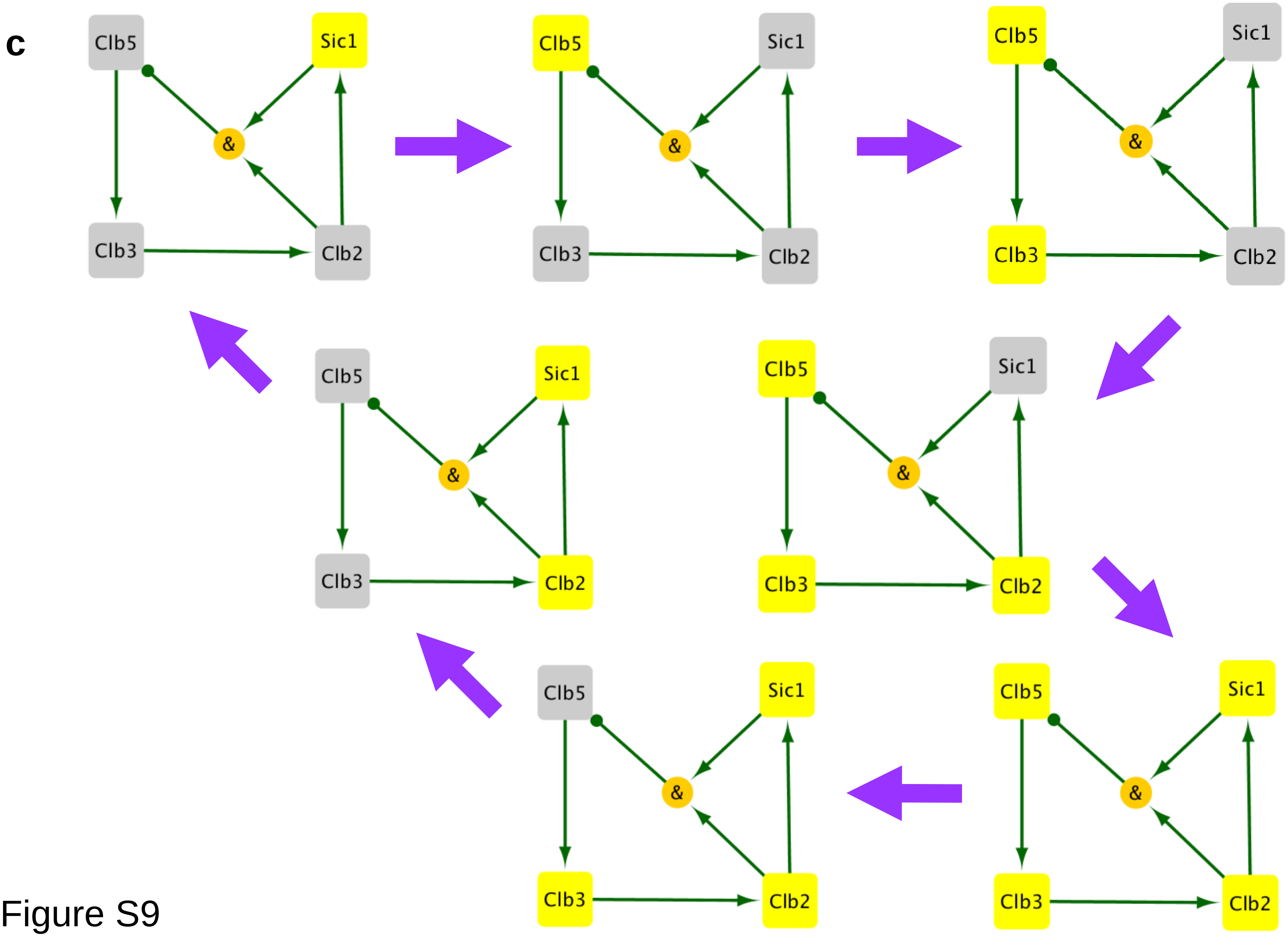
c
Clb5
Sic1
&
Clb3
Clb2
Clb5
Sic1
&
Clb3
Clb2
Clb5
Sic1
&
Clb3
Clb2
Clb5
Sic1
&
Clb3
Clb2
Clb5
Sic1
&
Clb3
Clb2
Clb5
Sic1
&
Clb3
Clb2
Clb5
Sic1
&
Clb3
Clb2

Figure S9

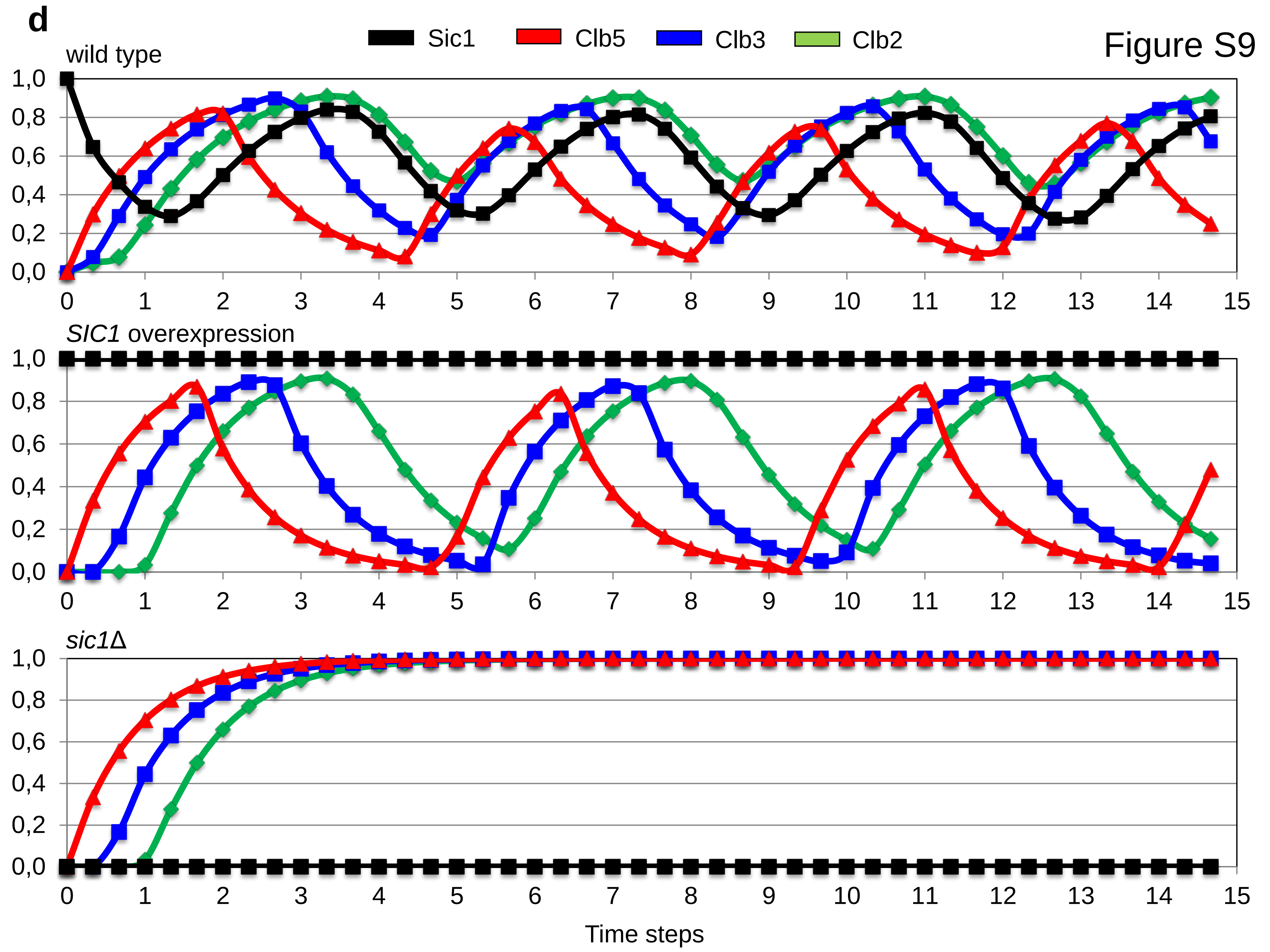

d
Figure S9
Sic1
Clb5
Clb3
Clb2
wild type
SIC1 overexpression
sic1Δ
1,0
0,8
0,6
0,4
0,2
0,0
0 1 2 3 4 5 6 7 8 9 10 11 12 13 14 15
Time steps

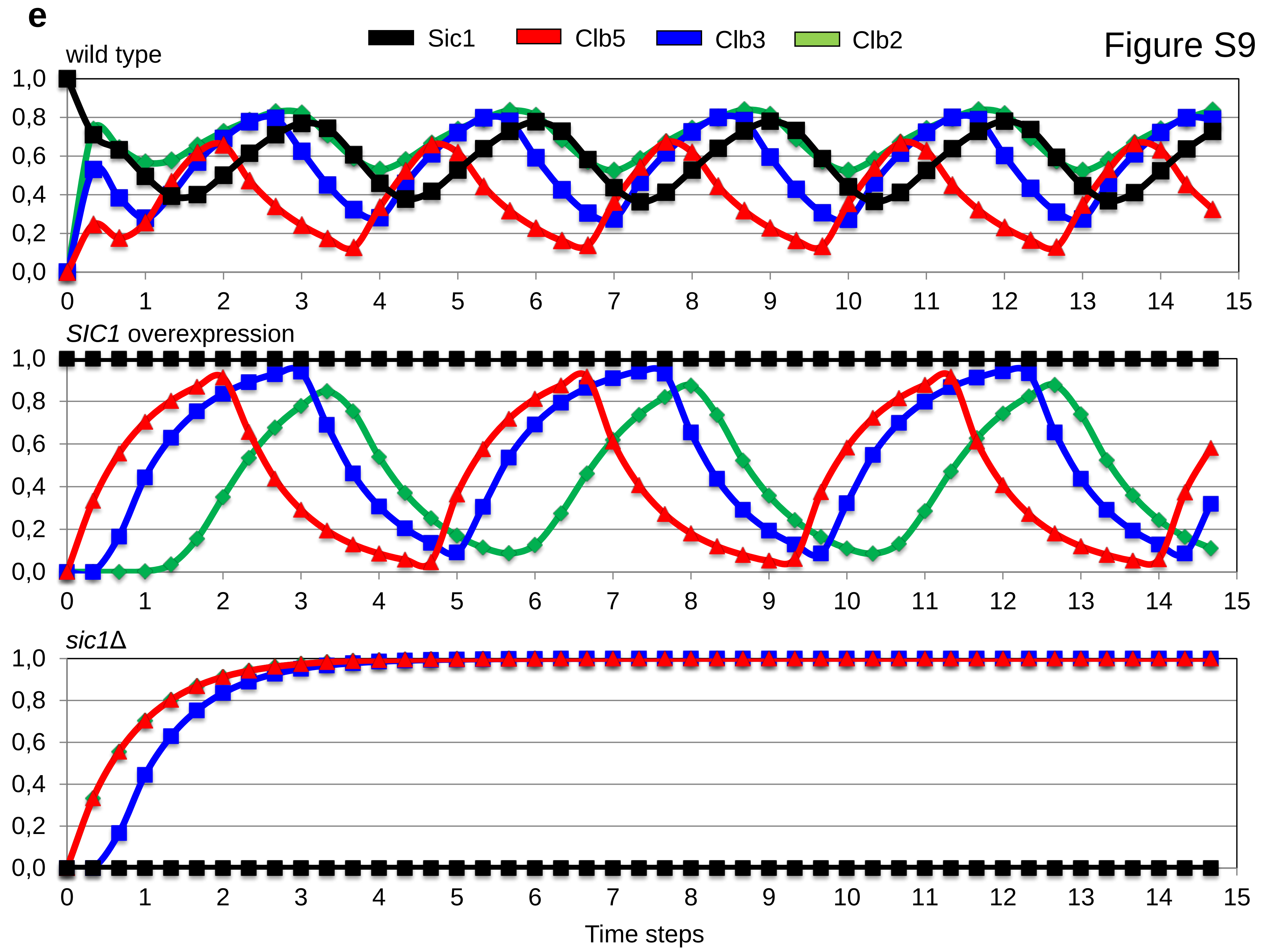
e
Figure S9
Sic1
Clb5
Clb3
Clb2
wild type
SIC1 overexpression
sic1Δ
1,0
0,8
0,6
0,4
0,2
0,0
0 1 2 3 4 5 6 7 8 9 10 11 12 13 14 15
Time steps

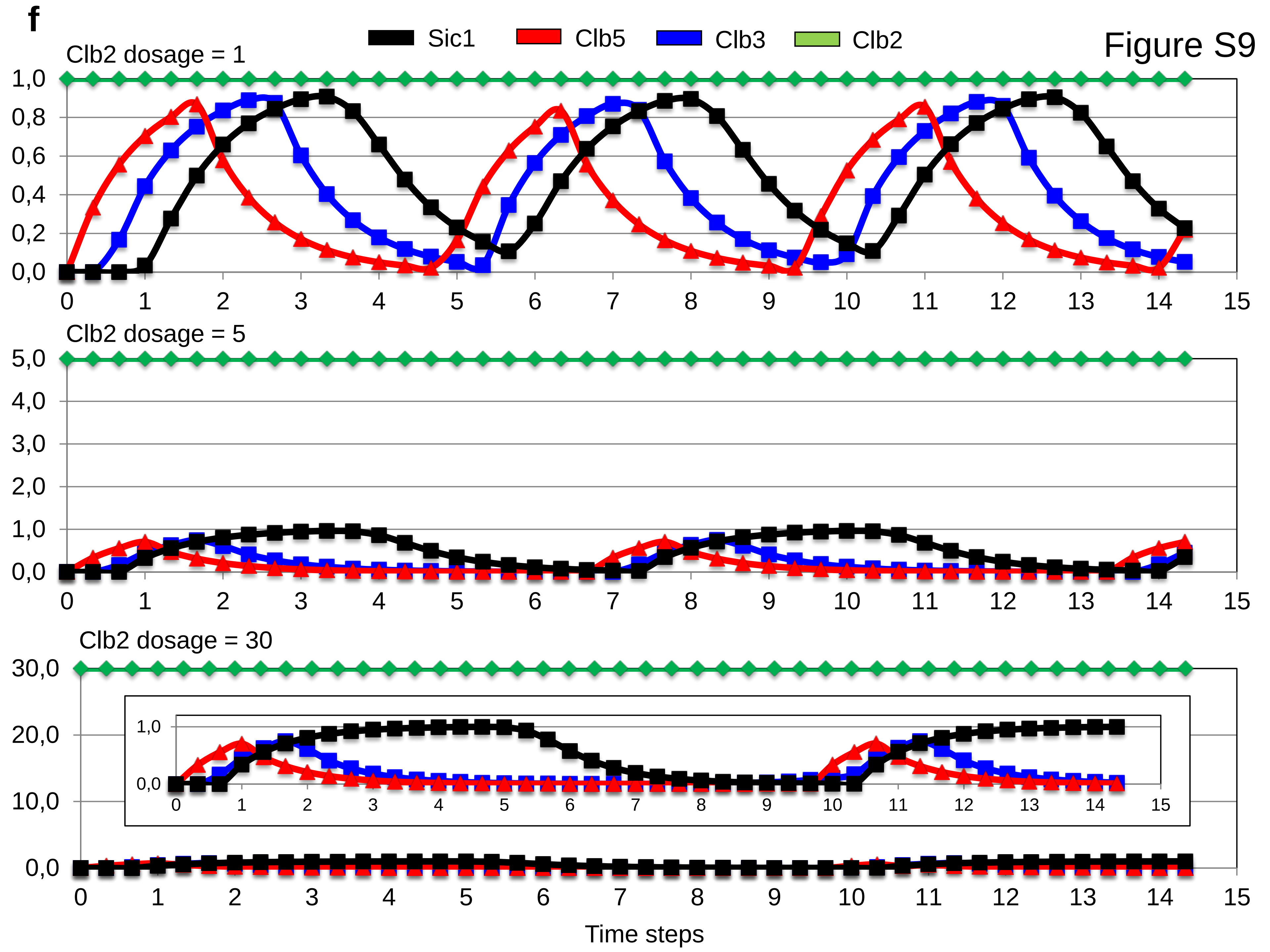
f
Figure S9
Sic1
Clb5
Clb3
Clb2
Clb2 dosage = 1
Clb2 dosage = 5
Clb2 dosage = 30
Time steps

Figure S10

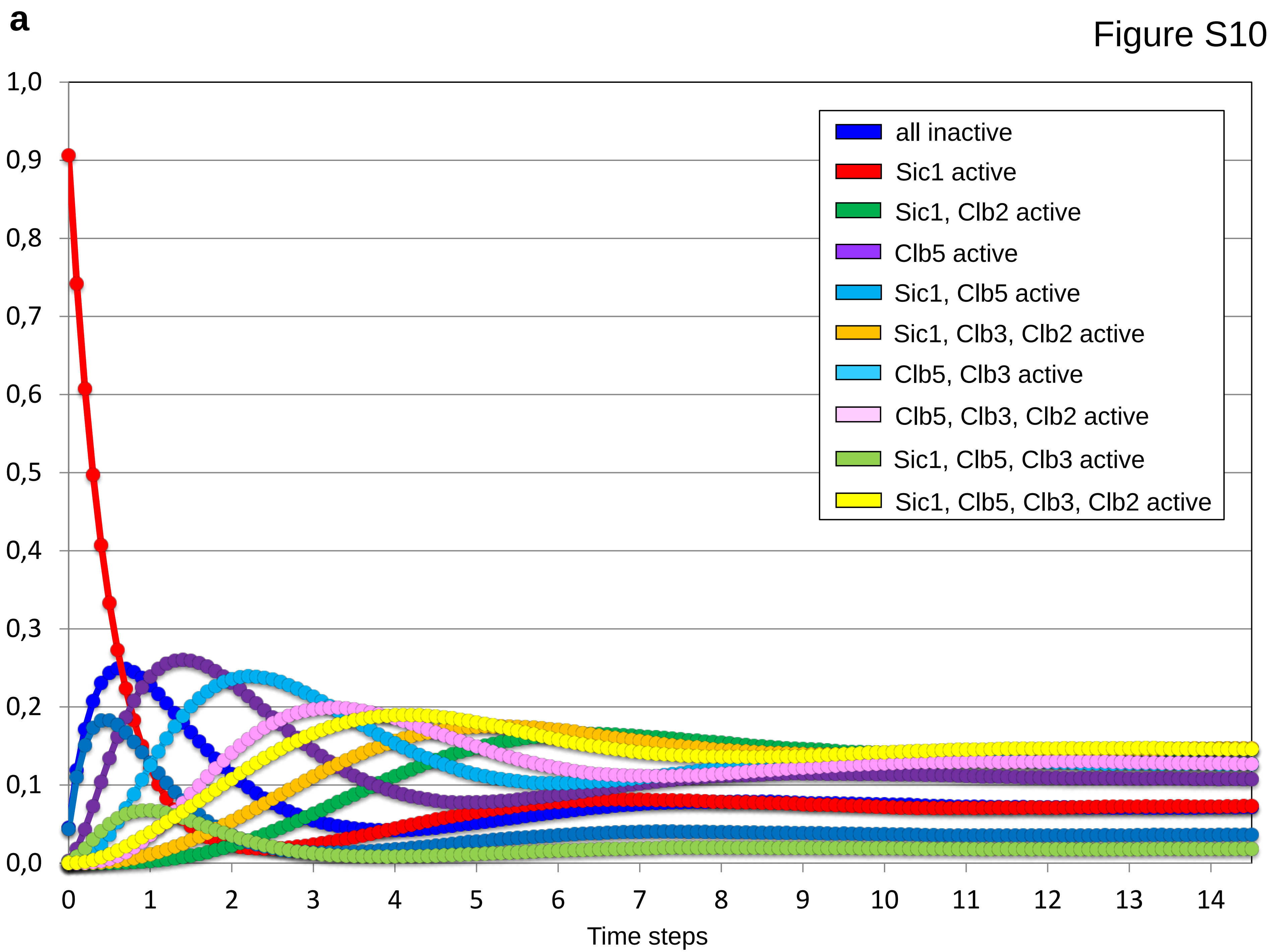
a
1,0
0,9
0,8
0,7
0,6
0,5
0,4
0,3
0,2
0,1
0,0
0
1
2
3
4
5
6
7
8
9
10
11
12
13
14
Time steps
all inactive
Sic1 active
Sic1, Clb2 active
Clb5 active
Sic1, Clb5 active
Sic1, Clb3, Clb2 active
Clb5, Clb3 active
Clb5, Clb3, Clb2 active
Sic1, Clb5, Clb3 active
Sic1, Clb5, Clb3, Clb2 active

Figure S10

b

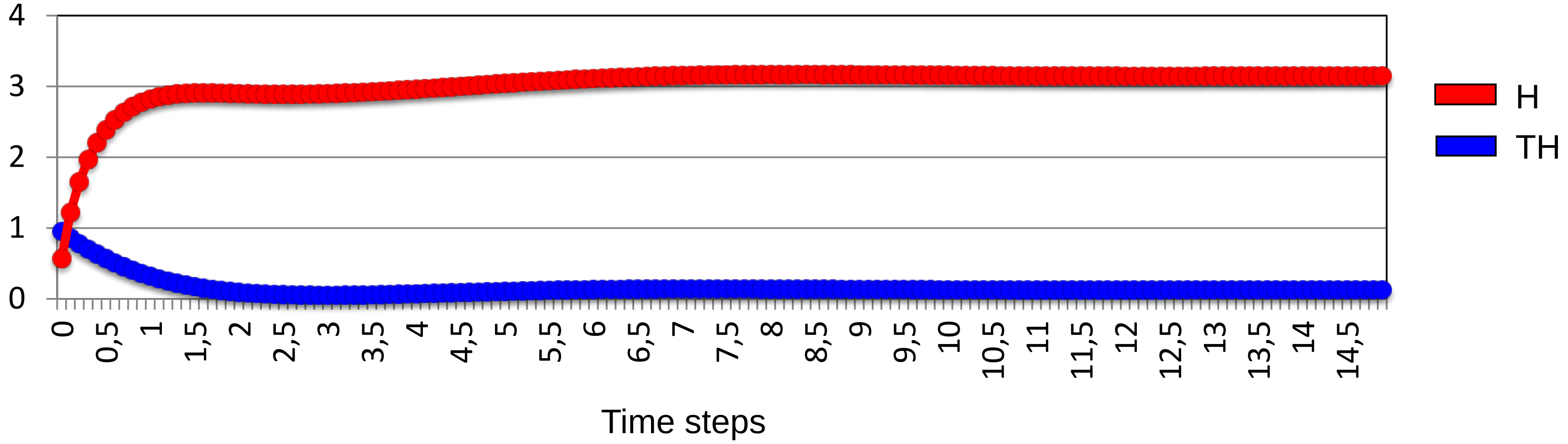

c

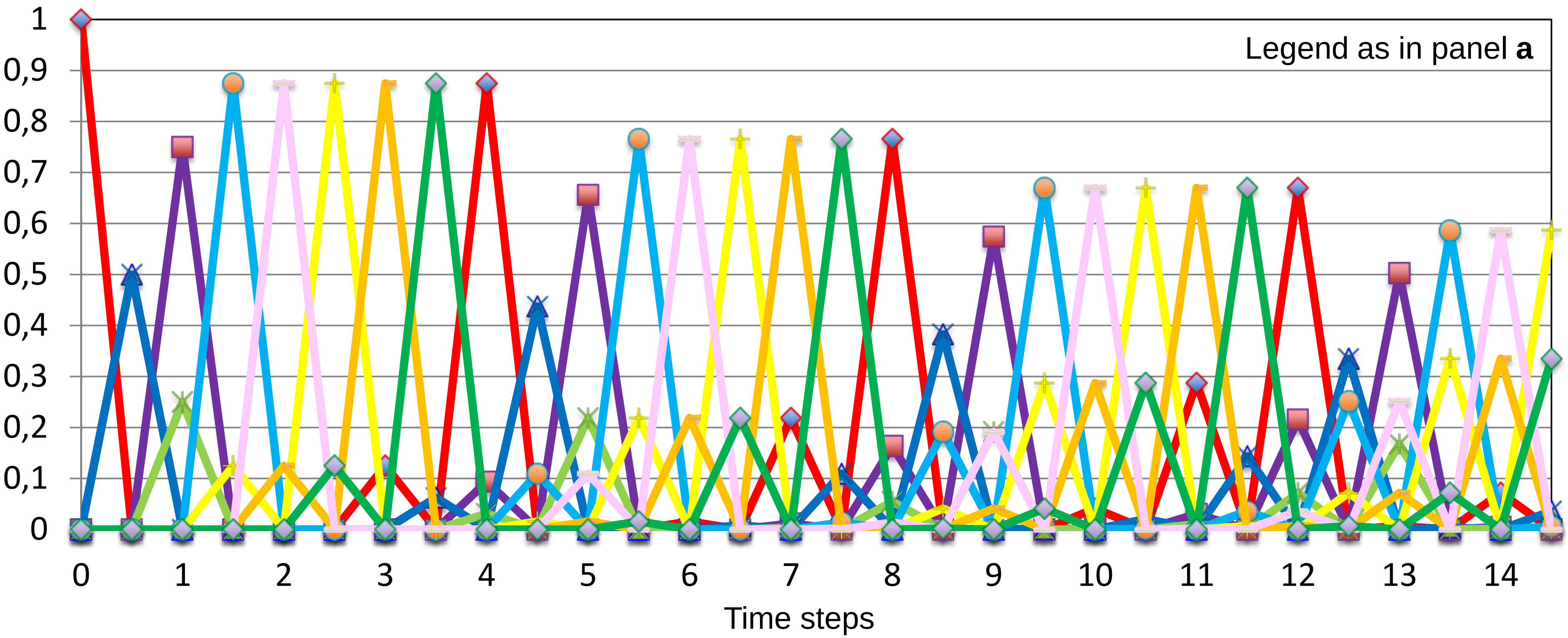

Supplement: Supplementary file 2 — Supplementary Figures [file 41540_2017_8_MOESM2_ESM.pdf]
